# Supplementary material for: BCL-XL blockage in TNBC models confers vulnerability to inhibition of specific cell cycle regulators
Source: Theranostics. 2021 Sep 3;11(19):9180–97. doi: 10.7150/thno.60503 (PMC8490507; doi:10.7150/thno.60503)

Supplementary data

BCL-XL blockage in TNBC models confers vulnerability to inhibition of specific cell cycle regulators

Olivier Castellanet[1,6], Fahmida Ahmad[1,6], Yaron Vinik[2], Gordon B. Mills[3], Bianca Habermann[1], Jean-Paul Borg[4,5], Sima Lev[2], Fabienne Lamballe[1,7], Flavio Maina[1,7]

[1] Aix Marseille Univ, CNRS, Developmental Biology Institute of Marseille (IBDM), Turing Center for Living Systems, Parc Scientifique de Luminy, Marseille (France). [2] Weizmann Institute of Science, Department of Molecular Cell Biology, Rehovot (Israel). [3] Knight Cancer Institute, Portland, OR 97201, USA. [4] Aix Marseille Univ, Centre de Recherche en Cancérologie de Marseille (CRCM), Equipe labellisée Ligue 'Cell polarity, cell signaling and cancer', Inserm, CNRS, Institut Paoli-Calmettes, Marseille (France). [5] Institut Universitaire de France (IUF).

[6] shared first authors

[7] shared senior and corresponding authors: fabienne.lamballe@univ-amu.fr; flavio.maina@univ-amu.fr

Supplementary Figure Legends

Figure S1. Human breast tumours are enriched in cell cycle regulators. High magnification of the STRING network reported in Figure 1A.

Figure S2. High expression levels of *BCL2*, *MCL-1*, alone or combined with *CDK1*, *CDK6*, and *WEE1*, correlate with lower survival rate of TNBC patients. Analysis of microarray data from 580 TNBC patients (GSE31519 Affymetrix Human Genome U133A Array). Kaplan-Meier curves report the probability of the overall survival of TNBC patients according to the expression levels of *BCL-2* **(A)** or *MCL-1* **(B)**, alone or in combination with specific cell cycle regulators (*CDK1*, *CDK6*, or *WEE1*). In the combinations, the expression levels indicated correspond to those of the cell cycle regulators and either *BCL-2* **(A)** *or MCL-1* **(B)**, respectively. The median of each gene expression levels was used as a threshold to segregate high versus low expressers. P values were computed using the Logrank (Mantel Cox) test.

Figure S3. *MMTV-R26^{Met}* TNBC cells are vulnerable to combined targeting of BCL-XL and CDK1/2/4. (A) RNA-seq data from *MMTV-R26^{Met}* tumours (n = 4) versus control mammary gland tissues (n = 3). Enrichment pathway analysis, using WikiPathways 2015, ordered according to the combined score. The 10-top ranked enriched pathways are shown and highlight enrichment in cell cycle/DNA replication (red arrowhead) and DNA damage (green arrowhead) regulatory pathways. The cut-off applied to identify deregulated genes was P value < 0.05. **(B)** GSEA enrichment performed, using the WikiPathways database with the DNA damage response geneset, on *MMTV-R26^{Met}* tumours versus controls. The barcode plot indicates the position of a member of this geneset in the ranked list. Red and blue colours represent up- or downregulated genes in the *MMTV-R26^{Met}* tumours versus the controls, respectively. NES: normalized enrichment score; FDR: false discovery rate. **(C)** Cell viability outcomes of combined drug effects (see detailed matrix, Figure 3G) were used by the Compusyn software to simulate combination index (Y-axis) for each affected fraction (X-axis, from 0 to 1). Black dots correspond to tested concentrations. Based on the combination index scores, co-targeting of BCL-

XL (with A1155463) and CDK1/2/4 (with R547) resulted in synergistic interactions for MGT4, MGT9, and MGT11 cells. **(D)** Cell viability assay performed on the non-tumorigenic *MMTV-R26^{Met}* MGT2 cell line as well as on mitomycin C-treated mouse embryonic fibroblasts (MEFs). **(E)** Heatmap reporting expression or phosphorylation levels of proteins in the four tumorigenic *MMTV-R26^{Met}* cell lines as determined by RPPA. **(F)** Comparable protein levels of Cyclin B1, D1, and E1 in the four tumorigenic *MMTV-R26^{Met}* cell lines untreated or treated with the indicated drugs (based on the RPPA analysis; Table S9). **(G)** Representative graphs showing cell cycle analysis as measured by flow cytometry using PI and Ki67 staining of MGT11 cells untreated (no) or treated with the indicated drugs: A1155463 (A11, 1 µM); R547 (3 µM).

Figure S4. Dose-response curves of R547 + A1155463 or Adavosertib + A1155463 drug combinations. The indicated cell lines were treated with varying concentrations of R547 and A1155463 (A11, 1 µM) (green line) or with varying concentrations of Adavosertib (Adav) and A1155463 (1 µM) (red line) for 48 h. The IC50 corresponding to each drug combination is indicated on the right.

Figure S5. Signalling perturbations in *MMTV-R26^{Met}* TNBC cell lines by blocking BCL-XL and CDK1/2/4. MGT13 cells were untreated (-) or treated for 12 h with either A1155463 (A11: 1 µM), R547 (3 µM) or Adavosertib (Adav: 3 µM), alone or in combination, then subjected to western blot analysis. Molecular weight markers (in kDa) are indicated on the right in all western blots. ACTIN and Ponceau (see also not edited gels) were used as loading controls.

Figure S6. Combined targeting of BCL-XL and CDK1/2/4 in *MMTV-R26^{Met}* MGT cells leads to downregulation of RTK and AKT signalling as well as of signals involved in cell cycle regulation and DNA damage/repair. High magnification of the STRING network reported in Figure 7B.

Figure S7. The A1155463 + R547 drug combination induces downregulation of both the endogenous MET and the MET transgene. Western blot analysis of endogenous mouse MET (mMET) and the MET

transgene (MET^{tg}) expression levels in the four *MMTV-R26^{Met}* MGT tumorigenic cell lines (MGT4, MGT9, MGT11, MGT13) after a 12 h treatment with the indicated drugs.

Supplementary Table Legends

Table S1 - List of drugs used in this study.

Table S2 - List of antibodies used in this study.

Table S3 - Quantification of western blot analyses.

Table S4 - List of oligonucleotides (Figure 8I).

Table S5 - Enrichr analysis on breast cancer patients (GSE42568, GSE45827 and GSE54002) (Figure 1B).

Table S6 - Microarray analysis of GSE31519 (TNBC patients) (Figure 2A-C).

Table S7 - Enrichr analysis on *MMTV-R26^{Met}* tumours (Figure 3B).

Table S8 - RNA-seq data on cell cycle regulators in *MMTV-R26^{Met}* tumours versus controls (Figure 3C).

Table S9 - All deregulated signals in treated MGT cells.

Table S10 - Antibodies used for RPPA analysis of *MMTV-R26^{Met}* treated cells.

Table S11 - Statistics of cell cycle distribution of MGT11 treated cells (Figure 5D).

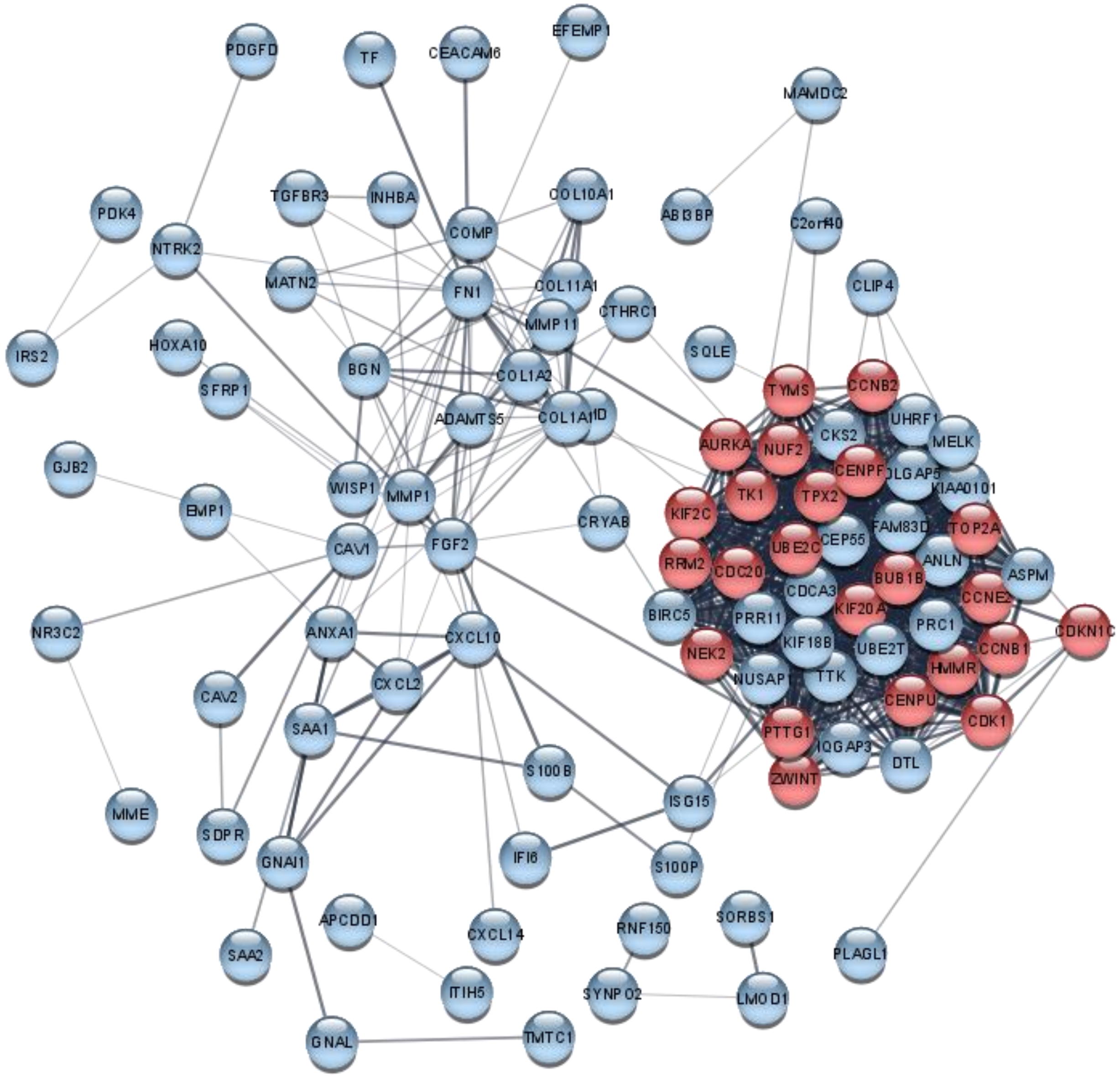
PDGFD
TF
CEACAM6
EFEMP1
MAMDC2
TGFBR3
INHBA
COL10A1
ABI3BP
C2orf40
PDK4
NTRK2
COMP
MATN2
FN1
COL11A1
CLIP4
IRS2
HOXA10
MMP11
CTHRC1
SQLE
SFRP1
BGN
COL1A2
TYMS
CCNB2
ADAMTS5
COL1A1
UHRF1
MELK
CKS2
AURKA
NUF2
CENPF
GJB2
WISP1
MMP1
TK1
TPX2
KIF2C
EMP1
CRYAB
FAM83D
TOP2A
CAV1
FGF2
UBE2C
CEP55
ANLN
RRM2
CDC20
BUB1B
ASPM
CDCA3
CCNE2
BIRC5
PRR11
KIF20A
PRC1
CDKN1C
NR3C2
ANXA1
CXCL10
KIF18B
UBE2T
CCNB1
NEK2
NUSAP1
TTK
HMMR
CAV2
CXCL2
CENPU
SAA1
PTTG1
CDK1
IQGAP3
DTL
S100B
ZWINT
ISG15
MME
SDPR
GNAI1
IFI6
S100P
APCDD1
CXCL14
RNF150
SORBS1
PLAGL1
SAA2
ITIH5
SYNPO2
LMOD1
GNAL
TMTC1

Figure S1

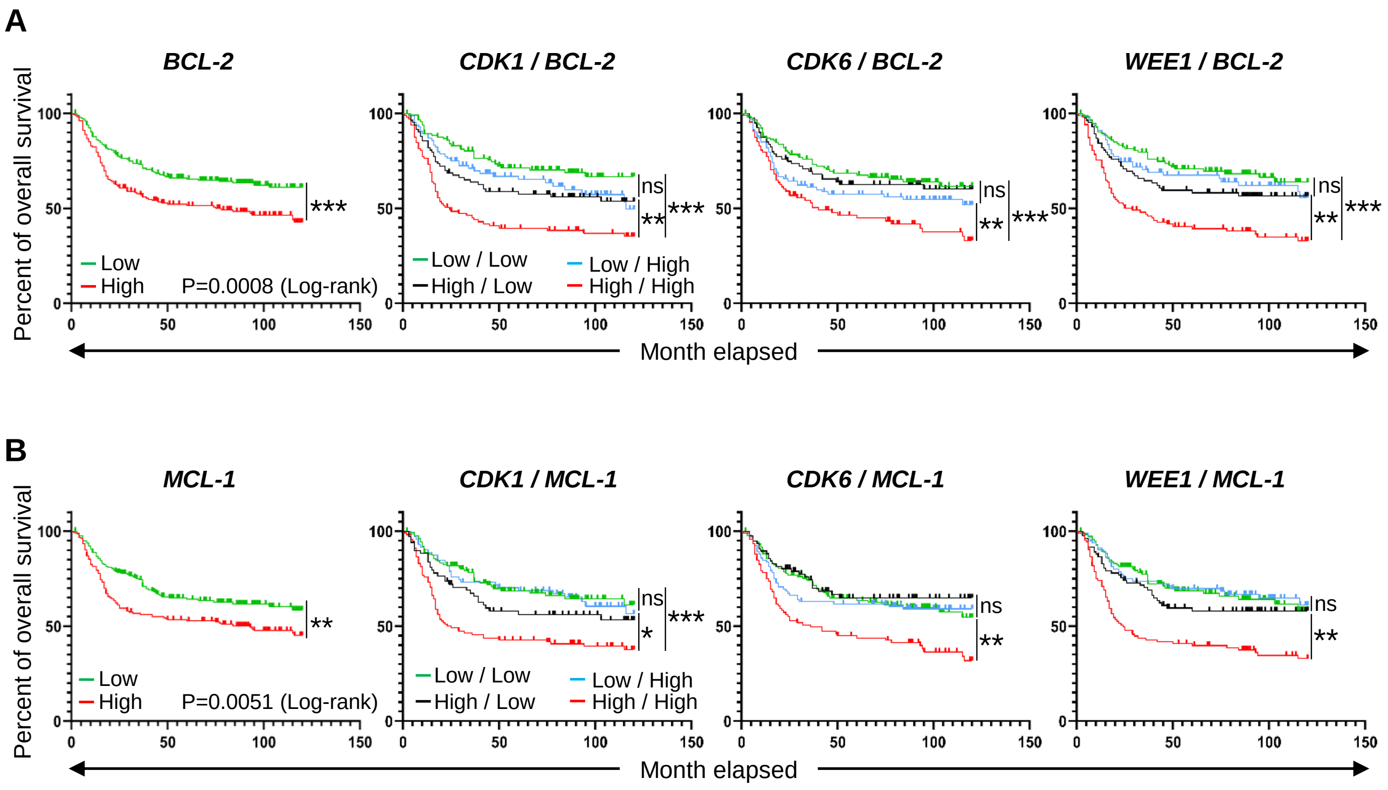
A
BCL-2
CDK1 / BCL-2
CDK6 / BCL-2
WEE1 / BCL-2
Percent of overall survival
Low
High
P=0.0008 (Log-rank)
Low / Low
High / Low
Low / High
High / High
ns
**
***
Month elapsed
B
MCL-1
CDK1 / MCL-1
CDK6 / MCL-1
WEE1 / MCL-1
Percent of overall survival
Low
High
P=0.0051 (Log-rank)
Low / Low
High / Low
Low / High
High / High
ns
*
**
***
Month elapsed

Figure S2

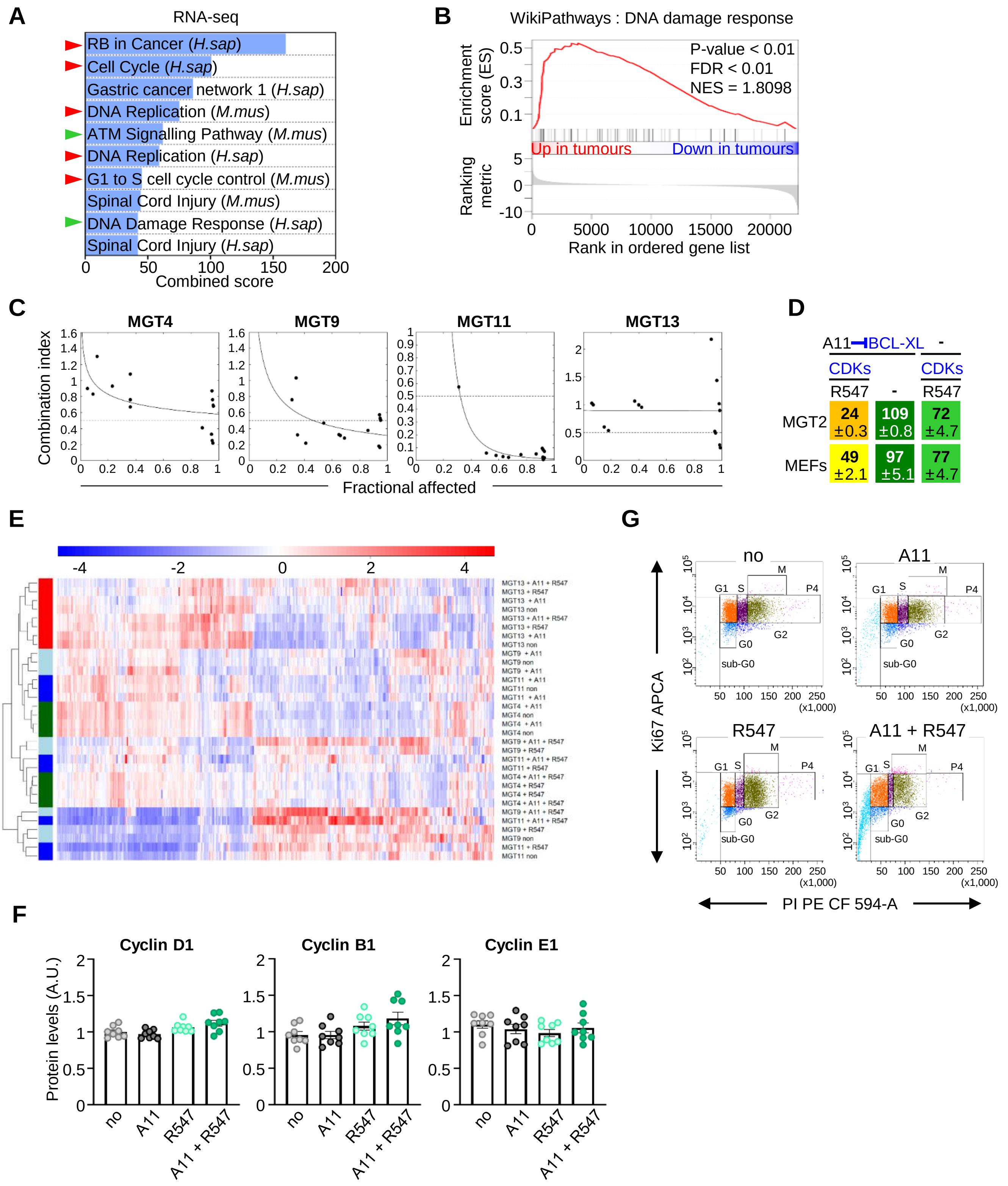
A
RNA-seq
RB in Cancer (H.sap)
Cell Cycle (H.sap)
Gastric cancer network 1 (H.sap)
DNA Replication (M.mus)
ATM Signalling Pathway (M.mus)
DNA Replication (H.sap)
G1 to S cell cycle control (M.mus)
Spinal Cord Injury (M.mus)
DNA Damage Response (H.sap)
Spinal Cord Injury (H.sap)
Combined score
B
WikiPathways : DNA damage response
P-value < 0.01
FDR < 0.01
NES = 1.8098
Enrichment score (ES)
Up in tumours
Down in tumours
Ranking metric
Rank in ordered gene list
C
MGT4
MGT9
MGT11
MGT13
Combination index
Fractional affected
D
A11 BCL-XL
CDKs
R547
MGT2
MEFs
24 ±0.3
109 ±0.8
72 ±4.7
49 ±2.1
97 ±5.1
77 ±4.7
E
G
no
A11
R547
A11 + R547
Ki67 APCA
PI PE CF 594-A
G1
S
M
G2
G0
P4
sub-G0
F
Cyclin D1
Cyclin B1
Cyclin E1
Protein levels (A.U.)
no
A11
R547
A11 + R547

Figure S3

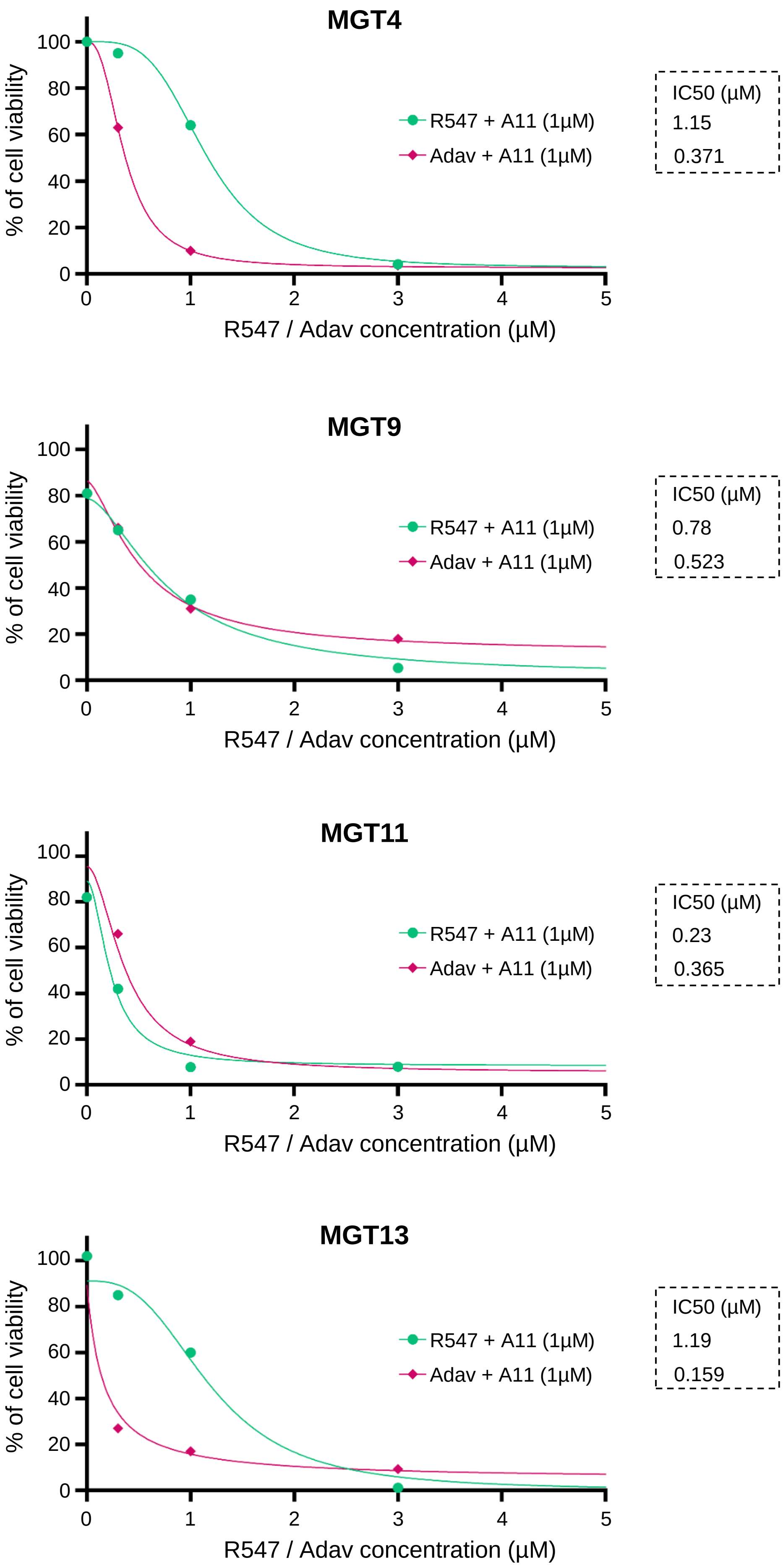
MGT4
% of cell viability
100
80
60
40
20
0
0
1
2
3
4
5
R547 / Adav concentration (µM)
R547 + A11 (1µM)
Adav + A11 (1µM)
IC50 (µM)
1.15
0.371
MGT9
% of cell viability
100
80
60
40
20
0
0
1
2
3
4
5
R547 / Adav concentration (µM)
R547 + A11 (1µM)
Adav + A11 (1µM)
IC50 (µM)
0.78
0.523
MGT11
% of cell viability
100
80
60
40
20
0
0
1
2
3
4
5
R547 / Adav concentration (µM)
R547 + A11 (1µM)
Adav + A11 (1µM)
IC50 (µM)
0.23
0.365
MGT13
% of cell viability
100
80
60
40
20
0
0
1
2
3
4
5
R547 / Adav concentration (µM)
R547 + A11 (1µM)
Adav + A11 (1µM)
IC50 (µM)
1.19
0.159

Figure S4

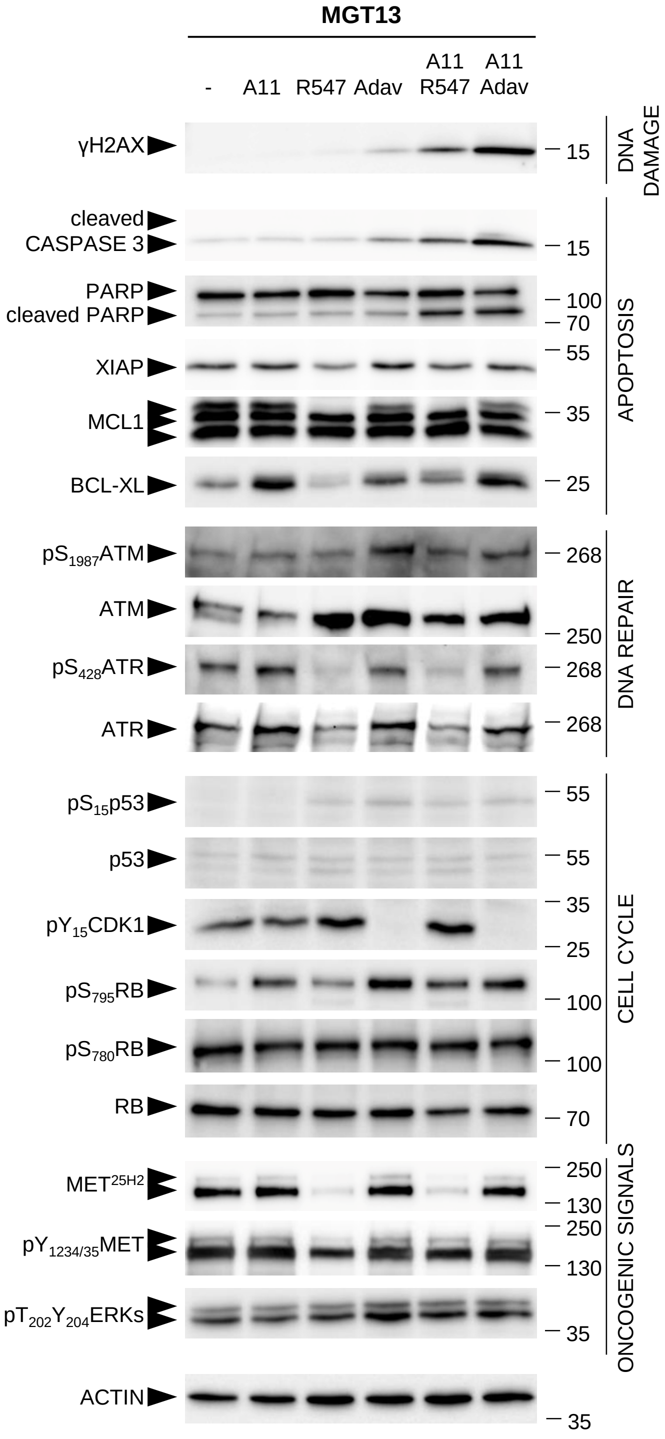
MGT13
-
A11
R547
Adav
A11 R547
A11 Adav
γH2AX
cleaved CASPASE 3
PARP
cleaved PARP
XIAP
MCL1
BCL-XL
pS1987ATM
ATM
pS428ATR
ATR
pS15p53
p53
pY15CDK1
pS795RB
pS780RB
RB
MET25H2
pY1234/35MET
pT202Y204ERKs
ACTIN
15
15
100
70
55
35
25
268
250
268
268
55
55
35
25
100
100
70
250
130
250
130
35
35
DNA DAMAGE
APOPTOSIS
DNA REPAIR
CELL CYCLE
ONCOGENIC SIGNALS

Figure S5

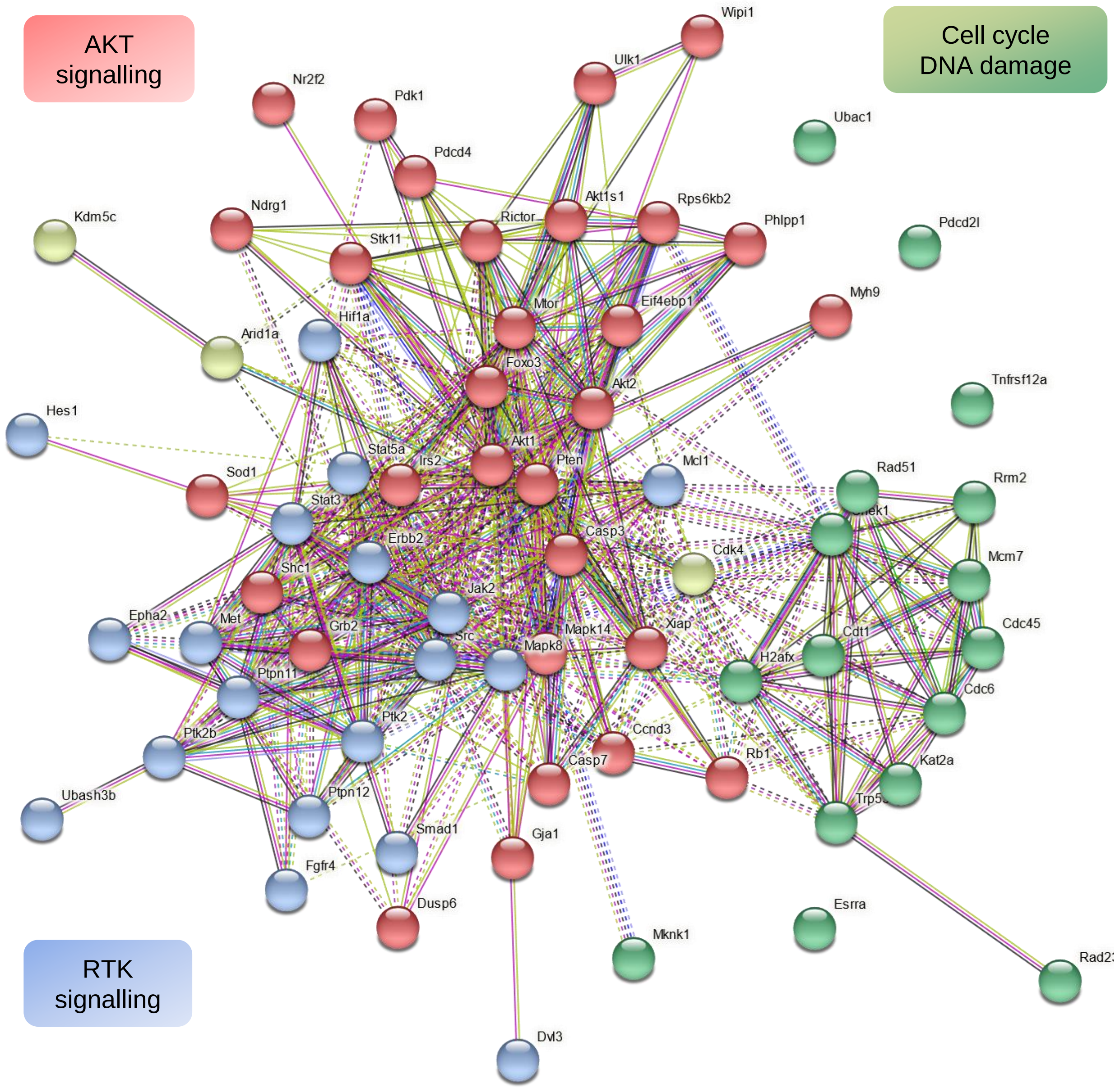

AKT signalling
Cell cycle DNA damage
RTK signalling
Wipi1
Ulk1
Nr2f2
Pdk1
Ubac1
Pdcd4
Akt1s1
Rps6kb2
Kdm5c
Ndrg1
Rictor
Phlpp1
Pdcd2l
Stk11
Myh9
Mtor
Eif4ebp1
Arid1a
Hif1a
Foxo3
Tnfrsf12a
Akt2
Hes1
Akt1
Stat5a
Irs2
Pten
Mcl1
Rad51
Rrm2
Sod1
Stat3
Casp3
Erbb2
Cdk4
Mcm7
Shc1
Jak2
Epha2
Met
Grb2
Xiap
Cdt1
Cdc45
Mapk14
Src
Mapk8
H2afx
Ptpn11
Cdc6
Ptk2
Ccnd3
Ptk2b
Rb1
Casp7
Kat2a
Ubash3b
Ptpn12
Smad1
Gja1
Fgfr4
Dusp6
Esrra
Mknk1
Dvl3

Figure S6

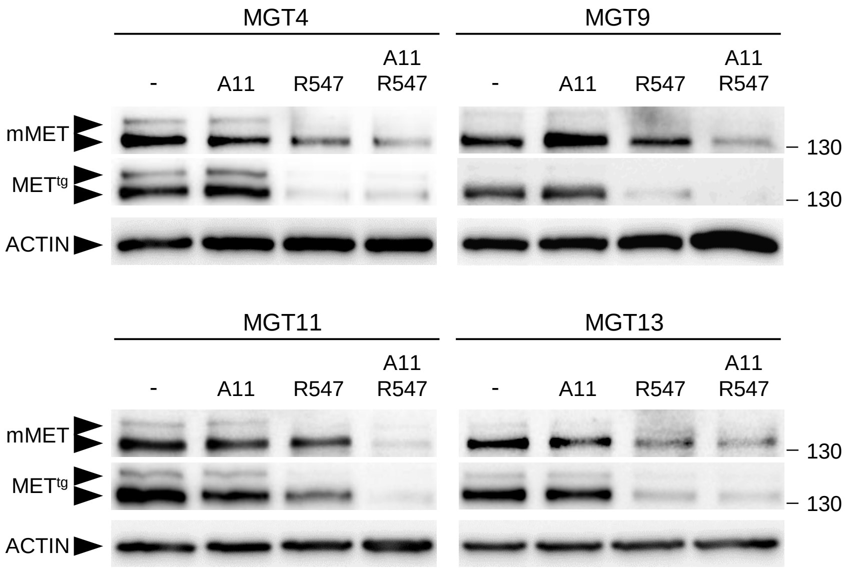
MGT4
MGT9
-
A11
R547
A11
R547
mMET
MET^tg
ACTIN
130
130
MGT11
MGT13

Figure S7

Table S1: Drugs used in this study, with the indicated targets and the concentrations used.

Drug	Company	Target	Concentration (µM)
A-1155463	Selleckchem/Targetmol	Bcl-xL	0.3, 1, 3, 10
Adavosertib	Selleckchem	Wee1	1, 3, 10
Adavosertib(MK-1775)	Targetmol	Wee1	1, 3, 10
Alisertib	Selleckchem	Aurora A	1, 3, 10
Barasertib	Selleckchem	Aurora B	1, 3, 10
FDI-6	Axon Medchem	FOXM1	0.3, 1, 3
R547	Selleckchem/Sigma	Cdk1/2/4	1, 3, 10

Table S2: List of antibodies used in the study.

Antibody	Company	Reference	Dilution	Used for	TritonX-100 (%) for IF
FOXO3a	CliniSciences	40937	1 :500	IF	0.5%
pS_{139}H2AX (γH2AX)	Cell Signaling	9718	1:400	IF	0.2%
Alexa488-conjugated Phalloidin	ThermoFisher Scientific	A12379	1:20	IF	0.5%
pH3 (S10)	Millipore	06-570	1 :500	IF	0.3%
alpha-TUBULIN	Sigma	T5168	1 :5000	IF	0.3%
ACTIN	Sigma	A3853	1:6000	WB	
AKT	Cell Signaling	9272	1:2000	WB	
pS_{473}AKT	Cell Signaling	9271	1:2000	WB	
ATM	Cell Signaling	2873	1:1000	WB	
pS_{1987}ATM	Invitrogen	PA5-37346	1:1000	WB	
ATR	Cell Signaling	13934	1:1000	WB	
pS_{428}ATR	Cell Signaling	2853	1:1000	WB	
BCL-2	Cell Signaling	3498	1:1000	WB	
BCL-XL	Transduction Laboratory	B22620	1:500	WB	
BIM	Santa Cruz	sc-11425	1:1000	WB	
Cleaved CASPASE 3	Cell Signaling	9661	1:1000	WB	
CDC2 (CDK1)	Cell Signaling	28493	1:20000	WB	
pY_{15}CDC2(CDK1)	Cell Signaling	4539	1:1000	WB	
$pT_{202/}Y_{204}$ERKs	Cell Signaling	9106	1:10000	WB	
ERKs	Cell Signaling	9102	1:10000	WB	
FOXM1	CliniSciences	32671	1:1000	WB	
pT_{600}FOXM1	CliniSciences	13207	1:1000	WB	
FOXO3a	CliniSciences	40937	1 :1000	WB	
pY_{627}GAB1	Cell Signaling	3231	1:2000	WB	
GAB1	Upstate	6579	1:1000	WB	
pS_{139}H2AX (γH2AX)	Cell Signaling	9718	1:1000	WB	
MCL-1	Santa Cruz	sc-819	1:1000	WB	
MET^{25H2}	Cell Signaling	3127	1:1000	WB	
MET (mouse)	Santa Cruz	sc-8057	1:200	WB	
MET (human)	Santa Cruz	sc-161	1:1000	WB	
$pY_{1234/35}$MET	Cell Signaling	3126	1:2000	WB	
PARP	Cell Signaling	9546S	1:2000	WB	

P53	Novocastra	CM5	1:1000	WB	
$pS_{15}P53$	Cell Signaling	9284	1:1000	WB	
RB	Cell Signaling	9313	1:1000	WB	
$pS_{780}RB$	Cell Signaling	9307	1:1000	WB	
$pS_{795}RB$	Abcam	Ab47474	1:1000	WB	
XIAP	Transduction laboratory	610716	1:3000	WB	
Goat anti-rabbit IgG-peroxidase	Jackson Immuno Research	115-035-144	1:4000	WB	
Goat anti-mouse IgG-peroxidase	Jackson Immuno Research	115-035-146	1:4000	WB	
anti-Ki67-APC (clone SolA15)	eBioscience	17-5698-82	1:200	FACS	

FACS: Fluorescence-Activated Cell Sorter; IF: immunofluorescence; WB: western-blot

Table S3A: for Figure 5A.

		no	A11	R547	Adav	A11+R547	A11+Adav
pS795RB	MGT4	1	0,9315527	0,16092187	1,54836092	0,07424294	0,97169236
	MGT9	1	1,01758044	0,34246868	0,99595888	0,24531738	0,66196819
	MGT11	1	0,95630636	0,41884003	1,12258585	0,19468984	0,61838129
	mean	1	0,96847983	0,30741019	1,22230188	0,17141672	0,75068061
pS780RB	MGT4	1	1,06384215	0,46787801	0,96006607	0,32445766	0,66937797
	MGT9	1	1,00885892	0,59724822	1,40492209	0,22889576	0,66056575
	MGT11	1	1,10483045	0,9750051	1,70358498	0,17024157	0,63100378
	mean	1	1,05917717	0,68004378	1,35619105	0,24119833	0,65364917
RB	MGT4	1	1,56126416	0,72186916	1,32097267	0,36647935	0,88828449
	MGT9	1	1,23970477	0,4361505	0,91853147	0,30467778	0,66417653
	MGT11	1	1,16229858	1,26224733	1,24972093	0,32727225	0,65935086
	mean	1	1,32108917	0,80675566	1,16307502	0,33280979	0,73727063
pS795RB/RB	MGT4	1	0,59666566	0,22292387	1,17213698	0,20258425	1,09389769
	MGT9	1	0,82082482	0,78520758	1,08429477	0,8051699	0,99667508
	MGT11	1	0,82277168	0,33182089	0,89826922	0,5948865	0,93786378
	mean	1	0,74675405	0,44665078	1,05156699	0,53421355	1,00947885
pS780RB/RB	MGT4	1	0,68139792	0,64814794	0,72678724	0,88533682	0,7535626
	MGT9	1	0,81378966	1,36936267	1,5295307	0,75127159	0,99456353
	MGT11	1	0,95055648	0,77243586	1,36317232	0,52018333	0,95700758
	mean	1	0,81524802	0,92998216	1,20649675	0,71893058	0,90171124
pT600 FOXM1/FOXM1	MGT4	1	0,97246009	0,3275371	1,21237692	0,2554552	1,70567484
	MGT9	1	1,16437667	0,25266219	1,11477097	0,24439447	1,09841105
	MGT11	1	1,43541527	0,24964528	2,29457399	0,29393335	2,76308765
	mean	1	1,19075068	0,27661485	1,54057396	0,26459434	1,85572451
pY15CDK1/CDK1	MGT4	1	0,99103697	0,9997345	0,17631286	0,86145415	0,16267749
	MGT9	1	1,13099606	1,02854996	0,15422014	1,03420767	0,16380551
	MGT11	1	1,00457991	1,20205007	0,19459357	1,37186597	0,17401194
	mean	1	1,04220431	1,07677818	0,17504219	1,08917593	0,16683165
pS15p53/p53	MGT4	1	0,96206479	1,22799201	1,9481385	1,24748956	2,22419023
	MGT9	1	1,01720249	1,55221515	4,94188662	2,7550666	3,52205026
	MGT11	1	1,03488188	0,99228339	1,95068887	1,33032459	1,31378801
	mean	1	1,00471639	1,25749685	2,94690467	1,77762691	2,35334283

Table S3B: for Figure 6A.

		no	A11	R547	Adav	A11+R547	A11+Adav
cleaved Caspase 3	MGT4	1	1,16330983	1,25998555	2,23364451	6,36530012	19,4941584
	MGT9	1	2,53702346	6,26904004	1,68322366	12,5739811	10,1590023
	MGT11	1	2,21488845	1,16767049	1,76485982	17,425839	20,3379615
	mean	1	1,97174058	2,89889869	1,89390933	12,1217067	16,6637074
cleaved PARP	MGT4	1	1,35565335	2,71191184	2,05256471	14,6108568	15,7039562
	MGT9	1	2,99725014	7,96017954	1,16991716	9,86735789	8,09219608
	MGT11	1	1,77442992	1,89411905	1,60699583	3,47886693	4,28487433
	mean	1	2,04244447	4,18873681	1,6098259	9,31902722	9,36034221
XIAP	MGT4	1	1,07480648	0,48145721	0,69264018	0,27242422	0,37583842
	MGT9	1	1,15759029	0,47683615	0,93803392	0,14450672	0,49017015
	MGT11	1	1,00858927	0,98559463	0,98525765	0,21607659	0,49784309
	mean	1	1,08032868	0,64796266	0,87197725	0,21100251	0,45461722
MCL-1	MGT4	1	1,13765363	0,67646782	0,73338944	0,53016182	0,6959116
	MGT9	1	0,90793218	0,44607936	0,65031359	0,28040793	0,4398872
	MGT11	1	1,01795224	1,23718197	1,02904342	0,26679576	0,60075284
	mean	1	1,02117935	0,78657638	0,80424882	0,35912183	0,57885055
BCL-XL	MGT4	1	1,39175671	0,79860408	0,83284638	0,86041748	0,77874487
	MGT9	1	1,05989868	0,64990877	0,85291073	0,34534101	0,64153777
	MGT11	1	1,27586203	1,01502702	1,08386304	0,67104466	1,19263456
	mean	1	1,24250581	0,82117996	0,92320672	0,62560105	0,8709724
γ-H2AX	MGT4	1	1,16583725	1,31531107	7,4426144	3,76549249	14,4775584
	MGT9	1	3,51128579	5,8791111	2,20015012	5,54221583	5,86724002
	MGT11	1	19,1639816	1,20852872	6,40873991	27,7304492	37,4890765
	mean	1	7,94703487	2,80098363	5,35050147	12,3460525	19,2779583
pS1987ATM	MGT4	1	1,29698385	1,44593876	1,29392539	1,23503123	1,33916345
	MGT9	1	0,79400189	0,67989518	0,74791365	0,46092035	0,64865896
	MGT11	1	0,9535647	1,11802719	1,80566134	0,78748604	1,08287957
	mean	1	1,01485015	1,08128704	1,28250013	0,82781254	1,02356733
ATM	MGT4	1	1,00905028	1,03598071	1,51083926	0,63173001	0,95409899
	MGT9	1	0,85106613	1,01360893	1,58629885	0,5827966	1,19342995
	MGT11	1	1,20354292	1,58001664	1,6070673	0,60536892	1,36200596
	mean	1	1,02121978	1,20986876	1,56806847	0,60663184	1,16984496
pS428ATR	MGT4	1	1,04321501	0,77108644	1,17346889	0,72106462	0,94027455
	MGT9	1	0,82213893	0,23625009	0,92913318	0,13067808	0,99802548
	MGT11	1	1,08350718	0,71769412	1,18214251	0,46428544	0,74709511
	mean	1	0,98295371	0,57501022	1,09491486	0,43867605	0,89513172
ATR	MGT4	1	1,23600716	0,82712045	1,15773943	0,82374835	1,04774557
	MGT9	1	0,60157325	0,35624405	0,45607655	0,2251816	0,58060096
	MGT11	1	1,01824229	0,92269771	1,27701094	0,56998185	0,86425439
	mean	1	0,9519409	0,70202074	0,96360897	0,53963727	0,83086698
pS1987ATM/ATM	MGT4	1	1,28535106	1,39571977	0,85642823	1,95499851	1,40358964
	MGT9	1	0,9329497	0,67076676	0,47148344	0,79087687	0,54352496
	MGT11	1	0,79229804	0,70760469	1,12357543	1,3008366	0,79506229
	mean	1	1,00353293	0,92469708	0,81716237	1,34890399	0,91405896
pS428ATR/ATR	MGT4	1	0,8440202	0,93225411	1,01358635	0,87534575	0,89742641
	MGT9	1	1,36664809	0,66316922	2,03723075	0,5803231	1,71895251
	MGT11	1	1,06409564	0,77782151	0,92571056	0,8145618	0,86443889
	mean	1	1,09158798	0,79108161	1,32550922	0,75674355	1,1602726

Table S3C: for Figure 8A.

		no	A11	R547	A11+R547
MET(25H2)	MGT4	1	0,82180823	0,08455866	0,06052498
	MGT9	1	1,21152279	0,35001143	0,24185877
	MGT11	1	1,14489546	0,28014391	0,1538484
	mean	1	1,05940883	0,238238	0,15207738
p(Y1234/1235)MET	MGT4	1	0,94986515	0,37028216	0,34298712
	MGT9	1	1,10060583	0,24401187	0,10363695
	MGT11	1	1,14289173	0,60639969	0,26599957
	mean	1	1,06445424	0,40689791	0,23754121
p(Y1234/1235)MET / MET	MGT4	1	0,86518411	0,22836277	0,1764643
	MGT9	1	1,10077809	1,43440328	2,33371183
	MGT11	1	1,0017532	0,46197898	0,57837836
	mean	1	0,98923847	0,70824834	1,02951816
p(T202Y204)ERKs	MGT4	1	1,03257184	0,3395853	0,47269481
	MGT9	1	1,18261689	0,23062938	0,3106951
	MGT11	1	1,18785422	0,33955436	0,48396292
	mean	1	1,13434765	0,30325635	0,42245094
ERKs	MGT4	1	0,91225905	0,79206133	0,66530427
	MGT9	1	1,02707225	0,92791321	0,59591159
	MGT11	1	1,07965474	0,84428145	0,72977894
	mean	1	1,00632868	0,854752	0,66366493
p(T202Y204)ERKs/ERKs	MGT4	1	1,13188446	0,42873611	0,71049418
	MGT9	1	1,1514447	0,24854628	0,52137784
	MGT11	1	1,10021674	0,40218148	0,66316372
	mean	1	1,12784863	0,35982129	0,63167858
p(Y627)GAB1	MGT4	1	1,1099289	0,43860793	0,40911731
	MGT9	1	1,15206773	0,62988797	0,60644738
	MGT11	1	1,14370314	0,84961841	0,87790666
	mean	1	1,13523326	0,63937144	0,63115712
GAB1	MGT4	1	1,02060188	0,35523056	0,32703936
	MGT9	1	0,93623969	0,29206129	0,29608801
	MGT11	1	0,94564087	0,52615788	0,14692367
	mean	1	0,96749415	0,39114991	0,25668368
p(Y627)GAB1 / GAB1	MGT4	1	1,08752387	1,23471341	1,25097271
	MGT9	1	1,23052647	2,15669788	2,04819968
	MGT11	1	1,20944767	1,61475945	5,97525689
	mean	1	1,17583267	1,66872358	3,09147643
p(S473)AKT	MGT4	1	0,78752341	0,6962135	0,57654437
	MGT9	1	1,00840485	0,99824448	0,51029255
	MGT11	1	1,0150818	0,8686713	0,39268555
	mean	1	0,93700335	0,85437643	0,49317416
AKT	MGT4	1	1,05239379	1,05220428	1,05093522
	MGT9	1	1,00128701	0,99866282	0,96925451
	MGT11	1	0,99928679	0,95442584	1,00807171
	mean	1	1,01765587	1,00176431	1,00942048
p(S473)AKT / AKT	MGT4	1	0,74831628	0,66167142	0,54860125
	MGT9	1	1,00710868	0,9995811	0,52647942
	MGT11	1	1,01580628	0,91015065	0,38954129
	mean	1	0,92374375	0,85713439	0,48820732

Table S3D: for Figure 8D.

	MDA-MB-231	SUM159	Hs578T	SK-BR-3	MCF-7	BT-474
MET(25H2)	0,6406167	0,69219191	1,02510996	0,38101784	0,14823882	0,14513178

Table S3E: for Figure 8F.

		no	A11	R547	A11+R547
MET(25H2)	MDA-MB-231	1	0,97758647	0,84313166	10,8451508
	SUM159	1	1,19311557	1,97791295	12,0494777
	Hs578T	1	1,00507505	0,90693291	6,35615738
p(T202Y204)ERKs	MDA-MB-231	1	0,52537124	0,68070719	0,16430478
	SUM159	1	1,00585718	0,59384647	0,33834688
	Hs578T	1	1,05593535	1,26306127	0,21151734
ERKs	MDA-MB-231	1	0,47432451	0,91799345	0,22878548
	SUM159	1	1,45748468	5,0990118	2,41601575
	Hs578T	1	1,20674408	5,30992943	7,10378408
p(T202Y204)ERKs/ERKs	MDA-MB-231	1	1,10761984	0,74151638	0,71816084
	SUM159	1	0,69013225	0,11646305	0,14004332
	Hs578T	1	0,8750284	0,23786781	0,02977531
p(S473)AKT	MDA-MB-231	1	1,03333305	1,80386758	0,40255631
	SUM159	1	1,10323277	0,73844985	0,24413652
	Hs578T	1	1,10380777	1,07440749	0,15811753
AKT	MDA-MB-231	1	0,65705213	0,75006786	0,08684719
	SUM159	1	1,07502142	0,89103699	0,15815285
	Hs578T	1	1,07693929	1,00863862	0,29881481
p(S473)AKT / AKT	MDA-MB-231	1	1,10761984	0,74151638	0,71816084
	SUM159	1	0,69013225	0,11646305	0,14004332
	Hs578T	1	0,8750284	0,23786781	0,02977531

Table S4: Oligonucleotides used for RT-qPCR experiments.

Oligonucleotide (name)	Sequence (Forward)	Sequence (Reverse)
PUMA	ACCGCTCCACCTGCCGTCAC	ACGGGCGACTCTAAGTGCTGC
Fas-L	GAAGGAACTGGCAGAACTCCGT	GCCACACTCCTCGGCTCTTTTT
B2M	ACAGTTCCACCCGCCTCACATT	TAGAAAGACCAGTCCTTGCTGAAG

Table S5. KEGG_2019_Human

Term	Overlap	P-value	Adjusted P-va	Old P-value	Old Adjusted	Odds Ratio	Combined Sc	Genes
Cell cycle	9/124	7,34E-08	1,01E-05	0	0	13,5665904	222,867345	CDC20;CDKN1C;CCNB2;CCNB1;PTTG1;CCNE2;CDK1;BUB1B;TTK
p53 signaling	5/72	8,16E-05	0,00372669	0	0	12,5284594	117,937959	CCNB2;CCNB1;RRM2;CCNE2;CDK1
Oocyte meios	7/125	1,23E-05	8,40E-04	0	0	10,1046902	114,274837	CDC20;CCNB2;CCNB1;PTTG1;CCNE2;CDK1;AURKA
ECM-receptor	5/82	1,52E-04	0,00519038	0	0	10,8958838	95,8253101	COMP;COL1A1;COL1A2;FN1;HMMR
Protein digest	5/90	2,35E-04	0,0053598	0	0	9,8664008	82,4539977	COL1A1;COL1A2;MME;COL11A1;COL10A1
Progesterone-	5/99	3,66E-04	0,00715502	0	0	8,91768842	70,5746889	CCNB2;CCNB1;CDK1;GNAI1;AURKA
Focal adhesio	7/199	2,33E-04	0,0053598	0	0	6,18691631	51,7468483	COMP;COL1A1;COL1A2;CAV2;PDGFD;CAV1;FN1
Pyrimidine me	3/57	0,00521504	0,06495098	0	0	9,17731481	48,2378767	RRM2;TK1;TYMS
Amoebiasis	4/96	0,00292675	0,04455164	0	0	7,22871757	42,1713458	COL1A1;COL1A2;GNAL;FN1
Drug metabol	4/108	0,00446298	0,06114286	0	0	6,3907563	34,5863775	RRM2;MAOA;FMO2;TK1
Bacterial inva	3/74	0,01071473	0,11213358	0	0	6,97394366	31,634756	CAV2;CAV1;FN1
PI3K-Akt signa	8/354	0,00157007	0,02688739	0	0	3,92681578	25,3540258	COMP;COL1A1;NTRK2;COL1A2;CCNE2;PDGFD;FN1;FGF2
Relaxin signali	4/130	0,00853441	0,0974345	0	0	5,26904095	25,0998624	COL1A1;COL1A2;MMP1;GNAI1
Gap junction	3/88	0,01705122	0,13846223	0	0	5,82117647	23,7011154	PDGFD;CDK1;GNAI1
Nitrogen meta	1/17	0,09959855	0,3591282	0	0	10,1746926	23,4690239	CA3
Phenylalanine	1/17	0,09959855	0,3591282	0	0	10,1746926	23,4690239	MAOA
Cocaine addic	2/49	0,03656234	0,21222213	0	0	6,97379989	23,0744662	MAOA;GNAI1
Mineral absor	2/51	0,03932841	0,21222213	0	0	6,68848035	21,6426382	TF;MT1M
IL-17 signaling	3/93	0,01972463	0,14222498	0	0	5,49638889	21,578202	CXCL10;MMP1;CXCL2
Small cell lung	3/93	0,01972463	0,14222498	0	0	5,49638889	21,578202	CCNE2;CKS2;FN1
Steroid biosyr	1/19	0,11064906	0,37451509	0	0	9,04326047	19,907759	SQLE
Regulation of	2/55	0,04508917	0,21222213	0	0	6,18244191	19,1600873	IRS2;GNAI1
AGE-RAGE sig	3/100	0,0238342	0,16326427	0	0	5,09793814	19,0491277	COL1A1;COL1A2;FN1
One carbon p	1/20	0,11612376	0,37451509	0	0	8,56686799	18,4453131	TYMS
Human T-cell	5/219	0,01145891	0,11213358	0	0	3,89335498	17,3993568	CDC20;CCNB2;PTTG1;CCNE2;BUB1B
Cellular senes	4/160	0,01718145	0,13846223	0	0	4,24929972	17,2688364	CCNB2;CCNB1;CCNE2;CDK1
Viral myocard	2/59	0,05113982	0,21894236	0	0	5,74742642	17,0882011	CAV1;DMD
Histidine meta	1/23	0,13234809	0,37451509	0	0	7,39754098	14,9601933	MAOA
Renin-angiote	1/23	0,13234809	0,37451509	0	0	7,39754098	14,9601933	MME
Platelet activa	3/124	0,04115729	0,21222213	0	0	4,08181818	13,0224458	COL1A1;COL1A2;GNAI1
RIG-I-like rece	2/70	0,06913496	0,28701483	0	0	4,81502188	12,8642688	CXCL10;ISG15
Human papillo	6/330	0,01639699	0,13846223	0	0	3,09480848	12,7216971	COMP;COL1A1;COL1A2;CCNE2;FN1;ISG15
Chemokine sig	4/190	0,02991776	0,19517778	0	0	3,55850727	12,48788	CXCL10;CXCL14;CXCL2;GNAI1

Melanoma	2/72	0,07260184	0,2925427	0	0	4,67697757	12,2666132	PDGFD;FGF2
Dopaminergic	3/131	0,04713409	0,21222213	0	0	3,85722656	11,782897	GNAL;MAOA;GNAI1
PPAR signaling	2/74	0,07612391	0,29797073	0	0	4,54660239	11,7092874	MMP1;SORBS1
FoxO signaling	3/132	0,04802107	0,21222213	0	0	3,82713178	11,619614	CCNB2;CCNB1;IRS2
Proteoglycans	4/201	0,03568244	0,21222213	0	0	3,35793201	11,1923116	CAV2;CAV1;FN1;FGF2
Human immu	4/212	0,04204774	0,21222213	0	0	3,17857143	10,0727331	CCNB2;CCNB1;CDK1;GNAI1
Regulation of	4/214	0,0432698	0,21222213	0	0	3,14797919	9,88559987	PDGFD;FN1;FGF2;IQGAP3
Tyrosine meta	1/36	0,19930744	0,49153762	0	0	4,64683841	7,49491679	MAOA
Prostate canc	2/97	0,12002643	0,37451509	0	0	3,44184428	7,29685903	CCNE2;PDGFD
Aldosterone-r	1/37	0,20424058	0,49153762	0	0	4,51753188	7,1759037	NR3C2
Pathways in ca	7/530	0,04545612	0,21222213	0	0	2,23310477	6,90254422	CCNE2;MMP1;FN1;CKS2;BIRC5;FGF2;GNAI1
Longevity regu	2/102	0,13025611	0,37451509	0	0	3,26892562	6,66289654	IRS2;CRYAB
Chagas diseas	2/103	0,132326	0,37451509	0	0	3,23639637	6,54556865	GNAL;GNAI1
Alcoholism	3/180	0,0996122	0,3591282	0	0	2,78248588	6,41772194	NTRK2;MAOA;GNAI1
Ferroptosis	1/40	0,21885981	0,49153762	0	0	4,16939891	6,33466728	TF
Glycine, serine	1/40	0,21885981	0,49153762	0	0	4,16939891	6,33466728	MAOA
Bladder cance	1/41	0,22367345	0,49424617	0	0	4,06495902	6,08755302	MMP1
Tryptophan m	1/42	0,22845766	0,49680474	0	0	3,96561375	5,85484959	MAOA
TNF signaling	2/110	0,14701818	0,40282981	0	0	3,02555862	5,80059803	CXCL10;CXCL2
Serotonergic s	2/113	0,15341456	0,41211362	0	0	2,94334003	5,51761901	MAOA;GNAI1
Viral carcinog	3/201	0,12705258	0,37451509	0	0	2,48472222	5,12636531	CDC20;CCNE2;CDK1
Epstein-Barr v	3/201	0,12705258	0,37451509	0	0	2,48472222	5,12636531	CXCL10;CCNE2;ISG15
Cytokine-cyto	4/294	0,10797968	0,37451509	0	0	2,27029846	5,05325813	CXCL10;INHBA;CXCL14;CXCL2
Type II diabet	1/46	0,24730381	0,51334276	0	0	3,61238616	5,04700089	IRS2
Rap1 signaling	3/206	0,13395065	0,37451509	0	0	2,4229064	4,87072954	PDGFD;FGF2;GNAI1
Arginine and p	1/49	0,26113811	0,52610294	0	0	3,38609973	4,5465359	MAOA
Malaria	1/49	0,26113811	0,52610294	0	0	3,38609973	4,5465359	COMP
Fanconi anem	1/54	0,28363701	0,53969819	0	0	3,06588308	3,86319665	UBE2T
Ubiquitin med	2/137	0,20619262	0,49153762	0	0	2,41714111	3,81653161	CDC20;UBE2C
Insulin signali	2/137	0,20619262	0,49153762	0	0	2,41714111	3,81653161	IRS2;SORBS1
Ras signaling p	3/232	0,1717482	0,45249046	0	0	2,14497817	3,77886337	NTRK2;PDGFD;FGF2
Legionellosis	1/55	0,28805456	0,54059554	0	0	3,00895568	3,74496241	CXCL2
Fluid shear str	2/139	0,21068578	0,49153762	0	0	2,38161308	3,70909437	CAV2;CAV1
Signaling path	2/139	0,21068578	0,49153762	0	0	2,38161308	3,70909437	INHBA;FGF2
Glutathione m	1/56	0,29244509	0,54141861	0	0	2,95409836	3,63199999	RRM2

Parkinson dise	2/142	0,21744393	0,49153762	0	0	2,33022432	3,55548945	GNAL;GNAI1
Gastric cancer	2/149	0,23328333	0,49937214	0	0	2,21847417	3,22899256	CCNE2;FGF2
Long-term de	1/60	0,30974027	0,56579222	0	0	2,7532648	3,22688465	GNAI1
Cushing syndr	2/155	0,24691631	0,51334276	0	0	2,13082699	2,98040008	CCNE2;GNAI1
Cytosolic DNA	1/63	0,32243586	0,57368458	0	0	2,61964569	2,96504871	CXCL10
Hepatitis B	2/163	0,26513575	0,52610294	0	0	2,02412607	2,68705434	CCNE2;BIRC5
Amphetamine	1/68	0,3430825	0,59447095	0	0	2,42353805	2,59266304	MAOA
cGMP-PKG sig	2/166	0,27197183	0,52610294	0	0	1,98679702	2,58692252	IRS2;GNAI1
Adipocytokine	1/69	0,34713632	0,59447095	0	0	2,38777724	2,5263584	IRS2
Renin secretic	1/69	0,34713632	0,59447095	0	0	2,38777724	2,5263584	GNAI1
Adherens junc	1/72	0,35914949	0,60004245	0	0	2,28653891	2,3414537	SORBS1
Arrhythmoger	1/72	0,35914949	0,60004245	0	0	2,28653891	2,3414537	DMD
MAPK signalir	3/295	0,27265189	0,52610294	0	0	1,67679795	2,17909857	NTRK2;PDGFD;FGF2
Gastric acid se	1/75	0,37094338	0,61132439	0	0	2,19350908	2,17531576	GNAI1
Pertussis	1/76	0,37482664	0,61132439	0	0	2,16415301	2,12366531	GNAI1
Kaposi sarcom	2/186	0,31740264	0,57216003	0	0	1,7690442	2,03012707	FGF2;CXCL2
Hypertrophic	1/85	0,40872305	0,64077486	0	0	1,93140125	1,7280585	DMD
Colorectal car	1/86	0,41237491	0,64077486	0	0	1,90858245	1,69066501	BIRC5
Salmonella inf	1/86	0,41237491	0,64077486	0	0	1,90858245	1,69066501	CXCL2
GABAergic syr	1/89	0,42319682	0,64077486	0	0	1,8432377	1,58503312	GNAI1
TGF-beta sign	1/90	0,42676001	0,64077486	0	0	1,82243507	1,55186445	INHBA
Dilated cardio	1/91	0,43030136	0,64077486	0	0	1,80209472	1,51965145	DMD
Morphine adc	1/91	0,43030136	0,64077486	0	0	1,80209472	1,51965145	GNAI1
Rheumatoid a	1/91	0,43030136	0,64077486	0	0	1,80209472	1,51965145	MMP1
NF-kappa B si	1/95	0,44425109	0,64285224	0	0	1,72506104	1,39965477	CXCL2
Circadian entr	1/97	0,45109836	0,64285224	0	0	1,6889515	1,3445234	GNAI1
Hematopoieti	1/97	0,45109836	0,64285224	0	0	1,6889515	1,3445234	MME
Choline metal	1/99	0,45786195	0,64285224	0	0	1,65431582	1,29233095	PDGFD
HIF-1 signaling	1/100	0,46121268	0,64285224	0	0	1,63752277	1,26727233	TF
Melanogenesi	1/101	0,46454286	0,64285224	0	0	1,62106557	1,24287332	GNAI1
Toll-like recep	1/104	0,47441141	0,64994363	0	0	1,57361133	1,17341109	CXCL10
Parathyroid h	1/106	0,48089	0,65229634	0	0	1,54348165	1,13000872	GNAI1
Endocytosis	2/244	0,44390972	0,64285224	0	0	1,34109692	1,08915051	CAV2;CAV1
Insulin resista	1/108	0,48728938	0,65400926	0	0	1,51447832	1,08875411	IRS2
Cholinergic sy	1/112	0,49985432	0,65400926	0	0	1,45960715	1,01214793	GNAI1

Leukocyte tra	1/112	0,49985432	0,65400926	0	0	1,45960715	1,01214793	GNAI1
Toxoplasmosi	1/113	0,50294753	0,65400926	0	0	1,44650176	0,99413645	GNAI1
Glutamatergi	1/114	0,50602176	0,65400926	0	0	1,43362832	0,97655264	GNAI1
Sphingolipid s	1/119	0,52111231	0,65869989	0	0	1,37253404	0,89460353	GNAI1
Neurotrophin	1/119	0,52111231	0,65869989	0	0	1,37253404	0,89460353	NTRK2
AMPK signalir	1/120	0,5240751	0,65869989	0	0	1,36093126	0,8793253	IRS2
Autophagy	1/128	0,547132	0,67425753	0	0	1,27468698	0,76871935	IRS2
Purine metab	1/129	0,54993507	0,67425753	0	0	1,26466445	0,75621251	RRM2
Vascular smo	1/132	0,55824144	0,67630873	0	0	1,23551495	0,7202604	PPP1R14A
Apelin signalir	1/137	0,5717488	0,67630873	0	0	1,18979026	0,66515884	GNAI1
Estrogen signa	1/137	0,5717488	0,67630873	0	0	1,18979026	0,66515884	GNAI1
Measles	1/138	0,57440071	0,67630873	0	0	1,18104583	0,65480492	CCNE2
MicroRNAs in	2/299	0,55121784	0,67425753	0	0	1,08968473	0,64904369	CCNE2;IRS2
Apoptosis	1/143	0,58741786	0,67630873	0	0	1,13917109	0,6060605	BIRC5
Adrenergic sig	1/145	0,59251345	0,67630873	0	0	1,12323543	0,58788088	GNAI1
Breast cancer	1/147	0,59754661	0,67630873	0	0	1,10773636	0,57039892	FGF2
Phospholipase	1/148	0,60004002	0,67630873	0	0	1,10014498	0,56190887	PDGFD
Retrograde er	1/148	0,60004002	0,67630873	0	0	1,10014498	0,56190887	GNAI1
Non-alcoholic	1/149	0,6025181	0,67630873	0	0	1,09265618	0,55358068	IRS2
Phagosome	1/152	0,60986134	0,67630873	0	0	1,07078493	0,52952848	COMP
Oxytocin signa	1/153	0,61227906	0,67630873	0	0	1,06368637	0,52180955	GNAI1
Hepatitis C	1/155	0,61707001	0,67630873	0	0	1,04976581	0,50679838	CXCL10
Wnt signaling	1/158	0,62414654	0,67830319	0	0	1,02954996	0,48529907	SFRP1
Hippo signalin	1/160	0,62879201	0,67830319	0	0	1,01649655	0,4716084	BIRC5
Protein proce	1/165	0,640158	0,68516911	0	0	0,9852559	0,43946379	CRYAB
Alzheimer dis	1/171	0,65334236	0,68852234	0	0	0,95019286	0,40445339	MME
Influenza A	1/171	0,65334236	0,68852234	0	0	0,95019286	0,40445339	CXCL10
NOD-like rece	1/178	0,66811958	0,69872047	0	0	0,91229045	0,3679159	CXCL2
Axon guidanc	1/181	0,67425978	0,69979992	0	0	0,896949	0,35352331	GNAI1
Transcriptiona	1/186	0,68424404	0,7035586	0	0	0,8724856	0,3310565	HOXA10
Calcium signal	1/188	0,68815221	0,7035586	0	0	0,86306654	0,32256701	GNAL
cAMP signalin	1/212	0,73146969	0,74230627	0	0	0,7639655	0,23889163	GNAI1
Human cytom	1/225	0,75238358	0,75791581	0	0	0,71915252	0,20460537	GNAI1
Olfactory tran	1/444	0,93733125	0,93733125	0	0	0,35958258	0,02327166	GNAL

Table S5. WikiPathways_2015

Term	Overlap	P-value	Adjusted P-va	Old P-value	Old Adjusted	Odds Ratio	Combined Scc	Genes
Gastric cancer	6/43	2,42E-07	1,77E-05	0	0	27,4982675	418,898028	TOP2A;TPX2;CENPF;UBE2C;S100P;AURKA
RB in Cancer(I	9/87	3,21E-09	4,68E-07	0	0	20,0394737	391,924624	TOP2A;CCNB2;ANLN;CCNB1;RRM2;CCNE2;CDK1;TTK;TYMS
Cell Cycle(Hon	7/104	3,63E-06	1,77E-04	0	0	12,3053679	154,130002	CDC20;CCNB2;CCNB1;PTTG1;CCNE2;CDK1;BUB1B
Catalytic cycle	1/5	0,03037691	0,13044201	0	0	40,7233607	142,290378	FMO2
Neurotransmi	1/5	0,03037691	0,13044201	0	0	40,7233607	142,290378	MAOA
Inflammatory	3/30	8,16E-04	0,01010409	0	0	18,3796296	130,693445	COL1A1;COL1A2;FN1
Inflammatory	3/31	9,00E-04	0,01010409	0	0	17,7223214	124,295002	COL1A1;COL1A2;FN1
Fluoropyrimid	3/35	0,00128675	0,01252437	0	0	15,5039063	103,188341	RRM2;TK1;TYMS
Gastric cancer	3/35	0,00128675	0,01252437	0	0	15,5039063	103,188341	TOP2A;UBE2C;UBE2T
miRNA regula	4/63	6,13E-04	0,01010409	0	0	11,2906993	83,5172474	CCNB2;CCNB1;CCNE2;CDK1
Focal Adhesio	8/186	1,99E-05	7,27E-04	0	0	7,698681	83,3253619	COMP;COL1A1;COL1A2;CAV2;PDGFD;CAV1;COL11A1;FN1
Hedgehog Sig	2/22	0,0079962	0,05306568	0	0	16,4107438	79,2440187	CCNB1;CDK1
Integrated Pa	8/195	2,80E-05	8,17E-04	0	0	7,32480818	76,7939415	TOP2A;ANXA1;GPRC5A;PTTG1;BUB1B;INHBA;TYMS;FGF2
Endochondral	4/67	7,74E-04	0,01010409	0	0	10,5716953	75,7331046	CDKN1C;ADAMTS5;COL10A1;FGF2
G1 to S cell cy	4/67	7,74E-04	0,01010409	0	0	10,5716953	75,7331046	CDKN1C;CCNB1;CCNE2;CDK1
DNA Damage	4/69	8,65E-04	0,01010409	0	0	10,2453782	72,259229	CCNB2;CCNB1;CCNE2;CDK1
Differentiatio	3/47	0,00302157	0,02594994	0	0	11,26875	65,3810548	TF;INHBA;FGF2
Senescence a	5/108	5,45E-04	0,01010409	0	0	8,13477045	61,1261816	COL1A1;FN1;COL10A1;INHBA;CXCL14
Focal Adhesio	7/183	1,39E-04	0,0033881	0	0	6,75484914	59,9785461	COL1A1;COL1A2;CAV2;PDGFD;CAV1;COL11A1;FN1
Type II interfe	3/50	0,00360385	0,02923119	0	0	10,5478723	59,3397317	CXCL10;IFI6;ISG15
Nanoparticle-	2/28	0,01277452	0,08109044	0	0	12,6198347	55,026299	COL1A1;FN1
Matrix Metall	2/29	0,01366612	0,08313558	0	0	12,1518212	52,1657675	MMP11;MMP1
Osteoblast(M	1/10	0,05983844	0,2184103	0	0	18,0947177	50,9566617	COL1A1
Matrix Metall	2/30	0,01458397	0,0851704	0	0	11,7172373	49,5385124	MMP11;MMP1
Endochondral	3/59	0,00574285	0,03992646	0	0	8,84866071	45,6573209	ADAMTS5;COL10A1;FGF2
Serotonin Tra	1/11	0,06562331	0,22255402	0	0	16,2844262	44,3559154	MAOA
Spinal Cord In	4/96	0,00292675	0,02594994	0	0	7,22871757	42,1713458	CXCL10;ANXA1;CDK1;CXCL2
Integrated Ca	2/35	0,01955477	0,10574061	0	0	9,93939394	39,1069034	MMP1;CDK1
Iron Homeost	1/12	0,07137288	0,22255402	0	0	14,8032787	39,0782475	TF
Iron metaboli	1/12	0,07137288	0,22255402	0	0	14,8032787	39,0782475	TF
miRNA Regula	4/105	0,00403731	0,03102352	0	0	6,58157917	36,278832	CCNB2;CCNB1;CCNE2;CDK1
Quercetin and	1/13	0,07708735	0,22255402	0	0	13,5689891	34,7748227	MMP1
Dopamine me	1/13	0,07708735	0,22255402	0	0	13,5689891	34,7748227	MAOA
ATM Signaling	2/40	0,02513048	0,12230166	0	0	8,62940409	31,7879108	CCNB1;CDK1
Spinal Cord In	4/114	0,00540304	0,03944222	0	0	6,04033613	31,5353437	CXCL10;ANXA1;CDK1;CXCL2
Osteoblast Sig	1/14	0,08276695	0,22255402	0	0	12,5245902	31,2078529	COL1A1

Biogenic Amin	1/14	0,08276695	0,22255402	0	0	12,5245902	31,2078529	MAOA
Biogenic Amin	1/15	0,08841187	0,22255402	0	0	11,6293911	28,209984	MAOA
Cholesterol Bi	1/15	0,08841187	0,22255402	0	0	11,6293911	28,209984	SQLE
Cholesterol Bi	1/15	0,08841187	0,22255402	0	0	11,6293911	28,209984	SQLE
GPCRs, Class C	1/15	0,08841187	0,22255402	0	0	11,6293911	28,209984	GPRC5A
Leptin Insulin	1/15	0,08841187	0,22255402	0	0	11,6293911	28,209984	IRS2
Regulation of	2/44	0,02999915	0,13044201	0	0	7,8059819	27,3723489	CDK1;KIF2C
Type II interfe	2/44	0,02999915	0,13044201	0	0	7,8059819	27,3723489	CXCL10;ISG15
GPCRs, Class C	1/16	0,09402234	0,22878769	0	0	10,8535519	25,6602159	GPRC5A
Neurotransmi	1/16	0,09402234	0,22878769	0	0	10,8535519	25,6602159	MAOA
ACE Inhibitor I	1/17	0,09959855	0,23838342	0	0	10,1746926	23,4690239	NR3C2
TGF Beta Signa	2/52	0,04074045	0,16640625	0	0	6,55438017	20,9775147	TGFBR3;INHBA
Cardiac Proge	2/53	0,04217145	0,16640625	0	0	6,42553881	20,3433322	INHBA;FGF2
IL-4 Signaling I	2/53	0,04217145	0,16640625	0	0	6,42553881	20,3433322	BIRC5;IRS2
Integrin-media	3/97	0,02202049	0,11482114	0	0	5,26143617	20,0764923	CAV2;CAV1;SORBS1
Nucleotide Me	1/19	0,11064906	0,25241816	0	0	9,04326047	19,907759	RRM2
TGF Beta Signa	2/55	0,04508917	0,17323734	0	0	6,18244191	19,1600873	TGFBR3;INHBA
Integrin-media	3/100	0,0238342	0,11999287	0	0	5,09793814	19,0491277	CAV2;CAV1;SORBS1
Nucleotide Me	1/20	0,11612376	0,26083182	0	0	8,56686799	18,4453131	RRM2
Nicotine Activ	1/21	0,12156503	0,2649029	0	0	8,13811475	17,1494977	GNAI1
G1 to S cell cy	2/60	0,05269574	0,19727122	0	0	5,64804788	16,6234515	CCNE2;CDK1
Chemokine sig	4/165	0,01900837	0,10574061	0	0	4,11628999	16,3123458	CXCL10;CXCL14;CXCL2;GNAI1
Oxidative Stre	1/22	0,12697307	0,26866766	0	0	7,75019516	15,9946996	MAOA
Signal Transdu	1/22	0,12697307	0,26866766	0	0	7,75019516	15,9946996	GNAI1
Angiogenesis(	1/23	0,13234809	0,27215242	0	0	7,39754098	14,9601933	FGF2
Cytokines and	1/23	0,13234809	0,27215242	0	0	7,39754098	14,9601933	CXCL2
Signal Transdu	1/24	0,13769029	0,27341557	0	0	7,07555239	14,0290403	GNAI1
IL-9 Signaling I	1/24	0,13769029	0,27341557	0	0	7,07555239	14,0290403	IRS2
PPAR signaling	2/68	0,06572515	0,22255402	0	0	4,96143251	13,5063766	MMP1;SORBS1
Cytokines and	1/25	0,14299986	0,27837305	0	0	6,78039617	13,1872716	CXCL2
Primary Focal	2/70	0,06913496	0,22255402	0	0	4,81502188	12,8642688	CDKN1C;MME
EPO Receptor	1/26	0,14827699	0,28114859	0	0	6,50885246	12,423272	IRS2
EPO Receptor	1/26	0,14827699	0,28114859	0	0	6,50885246	12,423272	IRS2
One Carbon M	1/28	0,15873477	0,29335793	0	0	6,02610808	11,0911759	TYMS
Extracellular v	1/28	0,15873477	0,29335793	0	0	6,02610808	11,0911759	TGFBR3
Apoptosis Mo	2/80	0,08700295	0,22255402	0	0	4,19559229	10,2448527	BIRC5;HN1
One Carbon M	1/30	0,16906518	0,29739176	0	0	5,60994912	9,97152177	TYMS
Prostaglandin	1/30	0,16906518	0,29739176	0	0	5,60994912	9,97152177	ANXA1

Dopminergic I	1/30	0,16906518	0,29739176	0	0	5,60994912	9,97152177	CDKN1C
TCA Cycle(Mu	1/30	0,16906518	0,29739176	0	0	5,60994912	9,97152177	PDK4
Prostaglandin	1/31	0,17418309	0,30084348	0	0	5,4226776	9,47693309	ANXA1
Integrated Bre	3/151	0,06639735	0,22255402	0	0	3,33260135	9,03834202	ANXA1;MMP1;AURKA
Oxidative Stre	1/32	0,17926975	0,30084348	0	0	5,2474881	9,01971654	MAOA
Statin Pathwa	1/32	0,17926975	0,30084348	0	0	5,2474881	9,01971654	SQLE
Trans-sulfurat	1/32	0,17926975	0,30084348	0	0	5,2474881	9,01971654	TYMS
G Protein Sign	2/88	0,10217292	0,24060075	0	0	3,80376706	8,67672967	GNAL;GNAI1
Alpha 6 Beta 4	1/33	0,18432532	0,30237636	0	0	5,08324795	8,59604194	IRS2
Amino Acid m	2/92	0,11001275	0,25241816	0	0	3,63397612	8,02076309	MAOA;PDK4
SIDS Susceptik	3/161	0,0771817	0,22255402	0	0	3,12009494	7,99241283	NTRK2;TF;MAOA
EGF/EGFR Sigr	3/161	0,0771817	0,22255402	0	0	3,12009494	7,99241283	CAV2;CAV1;AURKA
Endothelin Pa	1/35	0,19434398	0,31526913	0	0	4,78375121	7,83638527	GNAI1
G Protein Sign	2/96	0,11800568	0,26104288	0	0	3,47863548	7,43392223	GNAL;GNAI1
Striated Musc	1/38	0,20914356	0,33067346	0	0	4,39521489	6,8773438	DMD
Parkinsons Dis	1/40	0,21885981	0,33067346	0	0	4,16939891	6,33466728	CCNE2
Interleukin-11	1/40	0,21885981	0,33067346	0	0	4,16939891	6,33466728	BIRC5
Wnt Signaling	2/106	0,13858049	0,27341557	0	0	3,14256198	6,21065764	SFRP1;CDK1
Striated Musc	1/41	0,22367345	0,33067346	0	0	4,06495902	6,08755302	DMD
Splicing factor	1/42	0,22845766	0,33067346	0	0	3,96561375	5,85484959	CAV2
Aryl Hydrocarl	1/43	0,23321262	0,33067346	0	0	3,87099922	5,63541889	PLAGL1
Hair Follicle De	1/43	0,23321262	0,33067346	0	0	3,87099922	5,63541889	INHBA
IL-7 Signaling I	1/43	0,23321262	0,33067346	0	0	3,87099922	5,63541889	IRS2
One carbon m	1/44	0,23793852	0,33402907	0	0	3,78078536	5,42823602	TYMS
Apoptosis-rela	1/52	0,27472018	0,37838817	0	0	3,18643523	4,11688147	BIRC5
Vitamin B12 M	1/54	0,28363701	0,38613899	0	0	3,06588308	3,86319665	SAA2
Cardiac Hyper	1/56	0,29244509	0,39171544	0	0	2,95409836	3,63199999	FGF2
BDNF signaling	2/142	0,21744393	0,33067346	0	0	2,33022432	3,55548945	NTRK2;IRS2
Calcium Regul	2/143	0,21970111	0,33067346	0	0	2,31358068	3,506202	GJB2;GNAI1
Regulation of	2/144	0,22196034	0,33067346	0	0	2,29717146	3,45783246	FN1;FGF2
SIDS Susceptik	1/59	0,30545619	0,40542367	0	0	2,8008762	3,32169607	MAOA
Regulation of	2/148	0,23101551	0,33067346	0	0	2,23378241	3,27309535	FN1;FGF2
Calcium Regul	2/149	0,23328333	0,33067346	0	0	2,21847417	3,22899256	GJB2;GNAI1
Interferon typ	1/61	0,31399813	0,41300655	0	0	2,70724044	3,13598135	IRS2
miR-targeted g	4/362	0,18393964	0,30237636	0	0	1,83268391	3,10300445	COL1A1;CDKN1C;TYMS;IQGAP3
Alpha6-Beta4	1/64	0,32661604	0,42199949	0	0	2,57793391	2,88463066	IRS2
SREBP signalli	1/64	0,32661604	0,42199949	0	0	2,57793391	2,88463066	SQLE
Oncostatin M	1/65	0,33077064	0,42361854	0	0	2,53752561	2,8073409	MMP1

Insulin Signali	2/163	0,26513575	0,36866494	0	0	2,02412607	2,68705434	IRS2;SORBS1
TSH signaling	1/67	0,33900372	0,43038733	0	0	2,46038251	2,66150454	GNAI1
AMPK Signalir	1/69	0,34713632	0,43691295	0	0	2,38777724	2,5263584	CCNB1
Folate Metab	1/70	0,35116532	0,4382063	0	0	2,35305298	2,46246561	SAA2
EGFR1 Signali	2/172	0,28563706	0,38613899	0	0	1,9160914	2,40092633	CAV2;CAV1
Alzheimers Di	1/73	0,36310496	0,44926546	0	0	2,25466758	2,28412108	MME
Arrhythmoger	1/74	0,3670362	0,45031333	0	0	2,22366944	2,22877228	DMD
Alzheimers Di	1/79	0,3863343	0,46615544	0	0	2,08060109	1,97876027	MME
Apoptosis(Mu	1/79	0,3863343	0,46615544	0	0	2,08060109	1,97876027	BIRC5
MicroRNAs in	1/80	0,39012331	0,46686888	0	0	2,05416061	1,93356581	FGF2
Apoptosis(Ho	1/86	0,41237491	0,48948567	0	0	1,90858245	1,69066501	BIRC5
Selenium Mic	1/88	0,41961166	0,4940589	0	0	1,86451856	1,61919566	SAA2
Androgen rec	1/90	0,42676001	0,49449969	0	0	1,82243507	1,55186445	CAV1
Corticotropin-	1/90	0,42676001	0,49449969	0	0	1,82243507	1,55186445	GNAI1
IL-3 Signaling	1/97	0,45109836	0,51858552	0	0	1,6889515	1,3445234	BIRC5
Neural Crest D	1/101	0,46454286	0,5298692	0	0	1,62106557	1,24287332	FGF2
Toll-like recep	1/104	0,47441141	0,53693075	0	0	1,57361133	1,17341109	CXCL10
MicroRNAs in	1/109	0,49045966	0,55082393	0	0	1,50037948	1,0688887	FGF2
Iron uptake ar	1/115	0,50907714	0,56642886	0	0	1,42098073	0,95938328	TF
ESC Pluripoter	1/116	0,51211376	0,56642886	0	0	1,4085531	0,94261568	FGF2
Adipogenesis	1/129	0,54993507	0,59918298	0	0	1,26466445	0,75621251	IRS2
Metapathway	1/129	0,54993507	0,59918298	0	0	1,26466445	0,75621251	FMO2
Adipogenesis(	1/134	0,56369436	0,60526179	0	0	1,21681252	0,69752937	IRS2
PodNet: prote	2/306	0,5638055	0,60526179	0	0	1,0642127	0,60984277	CXCL10;BIRC5
Regulation of	1/146	0,59503778	0,63412786	0	0	1,11543245	0,57905486	CXCL10
Insulin Signali	1/153	0,61227906	0,6477735	0	0	1,06368637	0,52180955	SORBS1
Purine metab	1/158	0,62414654	0,65557838	0	0	1,02954996	0,48529907	RRM2
miR-targeted	1/166	0,64238943	0,66992041	0	0	0,97923497	0,43337078	TYMS
miR-targeted	1/172	0,65549268	0,67873711	0	0	0,94458825	0,39896398	FGF2
TNF-alpha NF-	1/179	0,67017896	0,68840509	0	0	0,90711917	0,36303861	CAV1
Metapathway	1/181	0,67425978	0,68840509	0	0	0,896949	0,35352331	FMO2
Non-odorant (	1/256	0,79596052	0,80701553	0	0	0,63072967	0,1439361	GPRC5A
PluriNetWork	1/284	0,82873423	0,83444964	0	0	0,56751434	0,10661084	UHRF1
mRNA proces	1/398	0,91625787	0,91625787	0	0	0,4021968	0,0351751	RBMS3

Table S6. GSE31519.

SampleName	datas	Array_type	GSM_accessic	Biops	nodal	age	tumo	grade	event	event	adjuv	206665_s_at Bcl	MEDIAN = -0,004	204252_at CDK2	MEDIAN = 0,004	203213_at Cdk1	MEDIAN = 0,006	202246_s_at Cdk	MEDIAN = 0,008	207143_at CDK6	MEDIAN = -0,003	202580_x_at Fox	MEDIAN= 0,0040	204092_s_at Aur	MEDIAN= 0,0044	209464_at Aurk	MEDIAN = 0,001	212533_at Wee1	MEDIAN = 0,005129
TNBC_001	1	GPL96	GSM782523	1	0	59	2	3	93	0	NO	-0,002958		0,003285		0,00418		0,008441		-0,000767		0,004304		0,006657		0,001442		0,004491	
TNBC_002	1	GPL96	GSM782524	1	0	65	2	12	37	0	NO	-0,002184		0,003536		0,006012		0,010858		-0,00565		0,000883		0,004826		0,000528		0,002893	
TNBC_003	1	GPL96	GSM782525	1	1	66	2	12	31	0	NO	-0,011604		0,004552		0,005921		0,008522		-0,003047		0,00161		0,00362		-0,00409		0,006735	
TNBC_004	1	GPL96	GSM782526	1	0						NO	-0,013867		0,004121		0,007375		0,006574		-0,000547		0,004008		0,00033		0,000694		0,006116	
TNBC_005	1	GPL96	GSM782527	1	1						NO	-0,005577		0,004448		0,004856		0,009406		-0,003133		0,001876		0,001655		0,00091		0,005595	
TNBC_006	1	GPL96	GSM782528	1	0	60	2	12	120	0	NO	-0,011456		0,004132		0,000932		0,008469		-0,008924		0,003279		0,002474		-0,001456		0,007706	
TNBC_007	1	GPL96	GSM782529	1	0	57	2	3	25	1	NO	-0,010638		0,004279		0,003992		0,008222		-0,002437		0,004311		0,003622		0,000774		0,007266	
TNBC_008	2	GPL96	GSM782530	1	1	46	1	12	23	1	YES	-0,005653		0,006488		0,002608		0,013323		-0,00193		-0,003385		0,002261		-0,004651		0,002943	
TNBC_009	2	GPL96	GSM782531	1	0	55	2	3	21	1	YES	0,00091		0,004673		0,004147		0,009802		-0,000725		0,00514		0,003852		0,00001		0,001386	
TNBC_010	2	GPL96	GSM782532	1	0	61	2	3	45	0	YES	-0,004565		0,003617		0,002205		0,007898		-0,004851		0,001676		0,001742		-0,001946		0,003999	
TNBC_011	2	GPL96	GSM782533	1	0	29	1	3	45	1	YES	-0,006712		0,004987		0,007575		0,009869		-0,00761		0,002873		0,003671		-0,001149		0,003628	
TNBC_012	2	GPL96	GSM782534	1		69	1	3	52	0	YES	-0,00074		0,005474		0,003782		0,008851		-0,002667		0,007149		0,006029		0,002048		0,003067	
TNBC_013	2	GPL96	GSM782535	1	1	47	2	3	16	1	YES	-0,000904		0,004052		0,003298		0,008554		-0,008195		0,000577		0,003768		0,0005		0,000241	
TNBC_014	2	GPL96	GSM782536	1	0	51	1	12	70	0	YES	-0,00406		0,004113		0,00553		0,009481		-0,003865		0,003475		0,000114		0,000858		0,002797	
TNBC_015	2	GPL96	GSM782537	1	0	51	2	12	77	0	YES	0,000635		0,001768		0,002073		0,007535		-0,001199		0,000497		0,002408		-0,000974		0,002325	
TNBC_016	2	GPL96	GSM782538	1	0	57	2	3	47	0	YES	-0,00809		0,005426		0,007126		0,010951		-0,003125		0,006831		0,004625		0,001995		0,007268	
TNBC_017	2	GPL96	GSM782539	1	1	80	2	3	11	1	YES	-0,000585		0,003954		0,002814		0,008472		-0,004793		0,005081		-0,000293		0,00247		0,004354	
TNBC_018	2	GPL96	GSM782540	1	0	48	2	3	76	0	YES	-0,002578		0,005595		0,003848		0,010732		-0,003981		0,006581		0,003435		0,000609		0,006098	
TNBC_019	2	GPL96	GSM782541	1	0	53	2	3	69	0	YES	-0,000529		0,005678		0,005096		0,011099		-0,002094		0,001432		0,002026		-0,000092		0,005187	
TNBC_020	2	GPL96	GSM782542	1	0	53	1	3	28	0	YES	-0,000726		0,006516		0,005703		0,011776		-0,002961		0,010893		0,002887		0,000743		0,004308	
TNBC_021	2	GPL96	GSM782543	1	0	67	2	12	34	0	YES	-0,000486		0,003464		-0,003234		0,006512		-0,005703		-0,006835		0,001113		-0,005604		0,002696	
TNBC_022	2	GPL96	GSM782544	1	0	56	2	3	10	1	YES	-0,001126		0,006658		0,004444		0,012656		-0,000172		0,00679		0,001872		0,005661		0,00452	
TNBC_023	2	GPL96	GSM782545	1	1	53	2	3	29	0	YES	0,000768		0,004916		0,00696		0,009014		0,001547		0,009019		0,003199		0,00342		0,005419	
TNBC_024	2	GPL96	GSM782546	1	0	63	1	3	36	0	YES	-0,001015		0,002052		-0,004994		0,010749		-0,011838		-0,005597		0,002378		-0,003245		0,000581	
TNBC_025	2	GPL96	GSM782547	1	0	47	2	3	31	0	YES	-0,000265		0,004724		0,004215		0,008398		-0,008395		0,008263		0,006453		0,003136		0,003372	
TNBC_026	2	GPL96	GSM782548	1	0	45	1	3	36	0	YES	-0,002184		0,004784		0,005162		0,010091		-0,003442		0,005687		0,003399		0,002833		0,001624	
TNBC_027	2	GPL96	GSM782549	1	0	42	2	3	6	1	YES	-0,002787		0,005446		0,009752		0,009427		-0,001479		0,008935		0,004591		0,00552		0,005911	
TNBC_028	2	GPL96	GSM782550	1	0	51	1	3	33	0	YES	-0,000543		0,004677		0,003572		0,010773		-0,004153		0,004828		0,003636		0,002191		0,003005	
TNBC_029	2	GPL96	GSM782551	1	0	54	2	3	10	1	YES	-0,000808		0,004457		0,001781		0,010468		-0,00635		-0,000426		0,006136		-0,000359		0,005349	
TNBC_030	2	GPL96	GSM782552	1	0	40	1	3	27	0	YES	-0,001569		0,004811		0,004632		0,009		-0,001711		0,005306		-0,001049		0,000706		0,001818	
TNBC_031	2	GPL96	GSM782553	1	0	57	1	3	45	0	YES	-0,001147		0,005407		0,004955		0,010092		-0,001816		0,006299		0,008985		0,000314		0,005251	
TNBC_032	3	GPL96 & GPL97	GSM79115	1	0	40	1	3	120	0	NO	-0,011548		0,005412		0,008804		0,007584		-0,00282		0,008651		0,006517		0,00404		0,006822	
TNBC_033	3	GPL96 & GPL97	GSM79117	1	1	52	2	3	120	0		-0,011003		0,00467		0,004631		0,008628		-0,00756		0,003087		0,012266		-0,00095		0,003048	
TNBC_034	3	GPL96 & GPL97	GSM79122	1	0	74	1	12	120	0	NO	-0,013772		0,007121		0,008747		0,008386		-0,000831		0,009217		0,00379		0,003362		0,004012	
TNBC_035	3	GPL96 & GPL97	GSM79145	1	0	40	2	12	120	0	NO	-0,004879		0,002342		0,000195		0,008314		-0,008118		0,000624		0,004364		-0,004371		0,00203	
TNBC_036	3	GPL96 & GPL97	GSM79147	1	1	34	2	3	8	0		-0,006145		0,004708		0,006217		0,006972		-0,003594		0,004995		0,006676		0,000755		0,007079	
TNBC_037	3	GPL96 & GPL97	GSM79165	1	0	62	2	12	120	0	NO	-0,002441		0,006255		0,004272		0,010526		-0,010984		0,00267		0,003482		-0,000267		0,00431	
TNBC_038	3	GPL96 & GPL97	GSM79191	1	0	58	2	12	120	0		-0,005865		0,005088		0,00703		0,009244		-0,009905		0,002835		0,006083		0		0,004622	
TNBC_039	3	GPL96 & GPL97	GSM79195	1	1	68	1	12	120	0		-0,006701		0,004367		0,003765		0,0064		-0,013064		-0,001054		0,006042		-0,000527		0,001882	
TNBC_040	3	GPL96 & GPL97	GSM79196	1	1	77	2	3	43	0	NO	-0,005028		0,004719		0,002901		0,009206		-0,006189		0,003752		0,006453		0,001509		0,00147	
TNBC_041	3	GPL96 & GPL97	GSM79225	1	0	70	1	12	95	0	NO	-0,016826		0,004308		0,000691		0,007763		-0,010364		-0,004064		0,008339		-0,010242		0,008007	
TNBC_042	3	GPL96 & GPL97	GSM79231	1	1	77	2	12	6	1	NO	-0,004089		0,006261		0,006623		0,008649		-0,007455		0,003402		-0,002002		-0,001628		0,006948	
TNBC_043	3	GPL96 & GPL97	GSM79251	1		88	2	3	2	0	NO	-0,011043		0,003467		0,006068		0,009799		-0,006671		0,004297		0,00628		-0,001734		0,003317	
TNBC_044	3	GPL96 & GPL97	GSM79253	1	0	75	1	3	30	1	NO	-0,005689		0,004389		0,004064		0,008208		-0,003454		0,002154		0,003617		0,002519		0,003291	
TNBC_045	3	GPL96 & GPL97	GSM79255	1	0	77	2	3	37	1	NO	-0,0111		0,005123		0,00361		0,007801		-0,011372		0,004813		0,005785		0,001785		0,004735	
TNBC_046	3	GPL96 & GPL97	GSM79270	1	0	32	1	12	120	0	NO	-0,006358		0,006321		0,008014		0,009331		-0,009067		0,009857		0,00605		0,001279		0,003349	
TNBC_047	3	GPL96 & GPL97	GSM79271	1	1	46	2	3	0	0	NO	-0,00309		0,006026		0,009656		0,009309		-0,006875		0,011511		0,005172		0,00224		0,00479	
TNBC_048	3	GPL96 & GPL97	GSM79280	1	0	50	1	3	120	0	NO	-0,004422		0,005152		0,003935		0,008235		-0,002556		0,003448		0,003369		0,001257		0,004787	
TNBC_049	3	GPL96 & GPL97	GSM79287	1	1	37	1	3	120	0		-0,015205		0,006182		0,003968		0,006642		-0,002632		0,004678		0,007053		0,000585		0,004135	
TNBC_050	3	GPL96 & GPL97	GSM79292	1	0	84	2	3	11	0		-0,006641		0,007176		0,006183		0,008626		-0,008168		0,004389		0,008377		0,002863		0,005993	
TNBC_051	3	GPL96 & GPL97	GSM79299	1	0	37	2	3	119	0	NO	-0,015931		0,007027		0,006428		0,0107		-0,004751		0,006109		0,007206		0,003114		0,006229	
TNBC_052	3	GPL96 & GPL97	GSM79303	1	0	44	1	12	118	0	NO	-0,010721		0,007147		0,002449		0,009195		-0,002971		0,004417		0,006516		-0,000201		0,00269	
TNBC_053	3	GPL96 & GPL97	GSM79306	1	0	38	2	3	2	1	NO	-0,019967		0,006497		0,006339		0,008399		-0,00721		0,00206		0,006706		0,001109		0,004833	
TNBC_054	3	GPL96 & GPL97	GSM79322	1	1	61	2	12	115	1	NO	-0,01387		0,004041		0,00557		0,007463		-0,004223		0,002366		0,006874		0,000728		0,004514	
TNBC_055	3	GPL96 & GPL97	GSM79329	1	0	68	1	3	7	1	NO	-0,014142		0,005359		0,006476		0,008634		-0,003573		0,007927		0,005432		0,001191		0,00428	
TNBC_056	3	GPL96 & GPL97	GSM79336	1	0	77	2	3	3	1	NO	-0,010975		0,006271		0,006898		0,00682		-0,006546		0,006507		0,007755		0,001607		0,005919	
TNBC_057	3	GPL96 & GPL97	GSM79344	1		87	1	3	120	0	NO	-0,008375		0,004694		0,006384		0,008299		-0,006797		0,001577		0,00716		0,00154		0,005858	
TNBC_058	3	GPL96 & GPL97	GSM79356	1	0	61	2	12	120	0	NO	-0,008706		0,004568		0,007379		0,007535		0,00082		0,011712		0,007616		0,003553		0,005036	
TNBC_059	4	GPL96	GSM782554	1	1	52	2	3	6	1	YES	-0,003684		0,005326		0,00621		0,010836		-0,001819		0,007425		0,007541		0,003466		0,003509	
TNBC_060	4	GPL96	GSM782555	1	0	41	2	3	67	0	YES	-0,000551		0,00241		0,000231		0,008649		-0,004159		0,000911		0,002795		-0,00111		-0,001178	
TNBC_061	4	GPL96	GSM782556	1	1	41	2	3	11	1	YES	-0,000661		0,002663		0,00138		0,008728		-0,005918		-0,000157		0,00127		-0,001395		0,001142	
TNBC_062	4	GPL96	GSM782557	1	0	30	2	3	51	0	YES	-0,002217		0,004407		0,003892		0,011167		-0,002618		0,005591		0,003285		-0,000803		0,001837	
TNBC_063	4	GPL96	GSM782558	1	0	59	1	12	48	0	YES	0,000549		0,005462		0,002925		0,008977		-0,007677		0,002863		0,003189		0,000008		0,003223	
TNBC_064	4	GPL96	GSM782559	1	1	71	2	3	58	0	YES	0,001572		0,002871		0,005044		0,008134		-0,002143		0,007754		0,004764		0,001942		0,005073	
TNBC_065	4	GPL96	GSM782560	1	0	43	2	3	3	1	YES	-0,000952		0,003716		0,003746		0,00752		-0,006218		0,004997		0,00281		-0,000417		0,003672	
TNBC_066	4	GPL96	GSM782561	1	0	60	1	3	48	0	YES	0,000886		0,004013		0,000943		0,009311		-0,010963		0,00851		0,004265		0,002905		0,0054	
TNBC_067	4	GPL96	GSM782562	1	0	31	1	3	10	1	YES	-0,001854		0,004275		0,003679		0,009295		-0,005749		0,006235		0,000156		0,000579		0,00244	
TNBC_068	4	GPL96	GSM782563	1	0	64	2	3	120	0	YES	-0,001913		0,004938		0,001578		0,010361		-0,006122		0,004235		0,005606		0,00079		0,00266	
TNBC_069	4	GPL96	GSM782564	1	0	33	1	3	74	1	YES	-0,002105		0,005312		0,003796		0,010123		-0,003942		0,003313		0,00492		0,002092		0,002638	
TNBC_070	4	GPL96	GSM782565	1	0	57	2		104	0	YES	-0,004577		0,004585		0,004376		0,010893		-0,006903		0,004924		0,004291		0,00115		0,001299	
TNBC_071	4	GPL96	GSM782566	1	0	32	2	3	22	1	YES	-0,000443		0,00563		0,006903		0,010099		-0,00378		0,006489		0,002874		0,002275		0,006818	
TNBC_072	4	GPL96	GSM782567	1	0	45	1	12	53	0	YES	0,00118		0,003688		0,000294		0,008932		-0,004699		0,002256		0,00128		0,00009		0,00294	
TNBC_073	4	GPL96	GSM782568	1	0	56	2	3	37	1	YES	-0,001876		0,005008		0,002204		0,008878		-0,002722		0,00349		0,004487		-0,000161		0,003964	
TNBC_074	6	GPL96	GSM65839	1	0	73	2	3	8	1	NO	-0,001833		0,006043		0,004431		0,008587		-0,00588		0,003477		0,000244		0,00046		0,006995	
TNBC_075	6	GPL96	GSM65841	1	0	64	1	12	120	0	NO	-0,003775		0,003982		0,004583		0,010091		-0,002982		0,004897		0,002895		-0,000788		0,002808	
TNBC_076	6	GPL96	GSM65842	1	0	47	2	3	120	0	NO	-0,010358		0,002367		0,005536		0,008351		-0,01069		0,006408		0,006144		0,00121		0,003767	
TNBC_077	6	GPL96	GSM65843	1	0	39	2	3	84	1	NO	-0,002436		0,003985		0,005392		0,007713		-0,004678		0,004669		0,003392		0,000295		0,003458	
TNBC_078	6	GPL96	GSM65845	1	0	32	2	3	120	0	NO	-0,013936		0,004441		0,007172		0,011528		-0,002691		0,007191		0,002758		0,001297		0,005304	

TNBC_079	6	GPL96	GSM65852	1	0	53	2		42	1	NO	-0,00992	0,006736	0,012103	0,010366	-0,003976	0,006856	0,00652	0,001025	0,008377
TNBC_080	6	GPL96	GSM65861	1	0	71	1	12	32	1	NO	-0,004172	0,002338	0,003221	0,008448	-0,005856	0,001294	0,001991	-0,000487	0,004506
TNBC_081	6	GPL96	GSM65865	1	0	43	2	3	120	0	NO	-0,007031	0,002045	-0,005918	0,006841	-0,007284	-0,006007	0,005259	-0,007696	0,005449
TNBC_082	6	GPL96	GSM65876	1	0	52	1	12	63	0	NO	-0,008284	0,002122	0,000571	0,007865	-0,003856	0,000201	0,004608	-0,002235	-0,001998
TNBC_083	6	GPL96	GSM65878	1	0	38	1	12	9	1	NO	-0,009007	0,004214	-0,000128	0,009513	-0,001339	0,001978	0,006161	-0,001548	0,004342
TNBC_084	6	GPL96	GSM65879	1	0	47	1		42	0	NO	-0,013014	0,00483	0,000715	0,008548	-0,00325	-0,001215	0,007566	-0,004362	0,003664
TNBC_085	6	GPL96	GSM150795	1	0	51	1				NO	-0,006167	0,003953	-0,001564	0,008535	-0,00532	-0,007194	0,003436	-0,004532	0,003766
TNBC_086	6	GPL96	GSM150797	1	0	29	2	3			NO	-0,008454	0,002065	0,000494	0,009653	-0,003045	-0,001833	0,006989	-0,005022	0,001803
TNBC_087	7	GPL96 & GPL97	GSM107076	1				3	15	1	NO	-0,005358	0,005688	0,006883	0,007789	0,000041	0	0,004357	0,00239	0,0068
TNBC_088	7	GPL96 & GPL97	GSM107084	1				3	13	1		0,003102	0,004616	0,008323	0,008134	-0,006507	0,003859	0,003027	0,001173	0,006318
TNBC_089	7	GPL96 & GPL97	GSM107094	1					49	1	NO	-0,001882	0,005442	0,003887	0,008429	0,000777	0,002005	0,004364	0,000532	0,002946
TNBC_090	7	GPL96 & GPL97	GSM107114	1				3	11	1	NO	-0,002671	0,005227	0,004921	0,007745	-0,004006	-0,001297	0,005601	-0,000801	0,007287
TNBC_091	7	GPL96 & GPL97	GSM107117	1				3	76	0	NO	-0,006871	0,005282	0,007129	0,007387	-0,005282	0,001976	0,005865	0,002061	0,006399
TNBC_092	7	GPL96 & GPL97	GSM107120	1				3	90	0	NO	-0,002546	0,004065	0,005583	0,011211	-0,005003	0,003127	0,002718	0,002769	0,001876
TNBC_093	7	GPL96 & GPL97	GSM107124	1				3	98	0	NO	-0,008804	0,003815	0,003563	0,009181	-0,0013	-0,006037	0,005213	-0,001593	0,003731
TNBC_094	7	GPL96 & GPL97	GSM107128	1				12	92	0	NO	-0,011896	0,004064	0,011526	0,010972	0,002069	0,005541	0,005228	0,000665	0,007019
TNBC_095	7	GPL96 & GPL97	GSM107131	1				3	76	0	NO	-0,002147	0,001923	-0,002594	0,007289	-0,005456	-0,012029	0,00213	-0,001655	0,005545
TNBC_096	7	GPL96 & GPL97	GSM107143	1				12	89	0	NO	-0,014151	0,003508	0,004838	0,008144	-0,00383	0,000887	0,003714	-0,000323	0,000847
TNBC_097	7	GPL96 & GPL97	GSM107144	1				3	79	0	NO	-0,005839	0,005319	0,008318	0,009678	-0,002479	0,004359	0,003901	0,005439	0,003159
TNBC_098	7	GPL96 & GPL97	GSM107148	1				12	73	0	NO	-0,009663	0,002617	-0,001651	0,008978	-0,004751	-0,001812	0,004332	-0,003221	0,002496
TNBC_099	7	GPL96 & GPL97	GSM107150	1				12	75	1	NO	-0,010101	0,010506	0,007139	0,009857	-0,000771	0,001704	0,004402	0,001055	0,003123
TNBC_100	7	GPL96 & GPL97	GSM107153	1				3	87	0	NO	-0,013718	0,005313	0,008207	0,00896	-0,005114	0,00004	0,007071	0,000159	0,004202
TNBC_101	7	GPL96 & GPL97	GSM107157	1				12	100	0	NO	-0,00548	0,00452	-0,0014	0,00744	-0,00388	-0,00616	0,000426	-0,00228	0,00476
TNBC_102	7	GPL96 & GPL97	GSM107167	1				3	98	0	NO	-0,00453	0,005865	0,004247	0,010435	-0,009424	0,000688	0,006582	0,000243	0,002386
TNBC_103	7	GPL96 & GPL97	GSM107181	1				3	13	1		-0,006794	0,005862	0,00691	0,011724	-0,001398	0,005513	0,007134	0,004076	0,005047
TNBC_104	7	GPL96 & GPL97	GSM107201	1				12	95	0	NO	-0,002982	0,003421	-0,003333	0,007938	-0,008377	-0,007149	0,004428	-0,003684	0,001886
TNBC_105	7	GPL96 & GPL97	GSM107204	1				3	82	0	NO	-0,006922	0,005444	0,002195	0,008779	-0,001182	0,000802	0,008293	0,001984	0,003461
TNBC_106	7	GPL96 & GPL97	GSM107206	1				3	17	1	NO	-0,007609	0,005315	0,006959	0,007839	-0,010592	0,001835	0,00375	0,00195	0,003556
TNBC_107	7	GPL96 & GPL97	GSM107210	1				3	77	1	NO	0,00094	0,00481	0,007103	0,009095	-0,007779	0,008042	0,005111	0,002631	0,003119
TNBC_108	7	GPL96 & GPL97	GSM107211	1				3	86	0		-0,004934	0,006368	0,008266	0,010838	-0,004133	0,006284	0,005679	0,002488	0,007338
TNBC_109	7	GPL96 & GPL97	GSM107217	1					18	1	NO	-0,006763	0,004457	0,007193	0,011611	-0,012002	0,002307	0,003023	0,00215	0,006177
TNBC_110	7	GPL96 & GPL97	GSM107224	1				3	72	0	NO	-0,003917	0,004406	0,002893	0,009569	-0,002492	0,003605	0,003052	0,004273	0,004095
TNBC_111	7	GPL96 & GPL97	GSM107225	1				3	19	1	NO	-0,004238	0,003714	0,005156	0,007996	-0,005505	0,004326	0,002694	0,003714	0,001966
TNBC_112	9	GPL570	GSM38092	1		55						0,003142	0,005399	0,008667	0,009933	0,000612	0,001371	0,00273	0,001687	0,006116
TNBC_113	9	GPL570	GSM46933	1		65						0,001155	0,007498	0,008674	0,010501	0,001092	0,008191	0,003536	0,005608	0,008779
TNBC_114	9	GPL570	GSM46938	1		65						0,001622	0,005624	0,008868	0,009993	0,000671	0,004737	0,00388	0,00292	0,006662
TNBC_115	9	GPL570	GSM46952	1		45						-0,001716	0,005983	0,010229	0,010734	-0,003322	0,008403	0,003868	0,004773	0,008887
TNBC_116	9	GPL570	GSM46968	1	0	45	2	3				0,001264	0,007803	0,011416	0,012103	0,000466	0,00951	0,002733	0,004611	0,008778
TNBC_117	9	GPL570	GSM53031	1		75						-0,005877	0,004122	0,006408	0,00835	-0,000931	-0,001808	0,002523	0,001569	0,005292
TNBC_118	9	GPL570	GSM53033	1		65						0,002442	0,006966	0,009923	0,010662	0,00569	0,007437	0,006854	0,004502	0,009139
TNBC_119	9	GPL570	GSM53172	1	1	55	2	3				-0,003082	0,004428	-0,000658	0,007121	0,001436	-0,011669	0,000532	0,000957	0,005535
TNBC_120	9	GPL570	GSM53187	1		65						0,003892	0,006127	0,011827	0,016874	0,000778	0,005097	0,000677	0,00236	0,006654
TNBC_121	9	GPL570	GSM89023	1	1	45	2	3				0,003518	0,005277	0,01	0,012819	0,00106	0,00588	0,002662	0,006843	0,006096
TNBC_122	9	GPL570	GSM102506	1	1	35	1	3				-0,000953	0,003245	0,012235	0,009453	0,002241	0,010251	0,004101	0,002009	0,00613
TNBC_123	9	GPL570	GSM102525	1	0	55	2	3				-0,000647	0,004992	0,014977	0,011744	0,001785	0,007424	0,005322	0,003673	0,011175
TNBC_124	9	GPL570	GSM102526	1	0	45	1	12				0,000046	0,003587	0,009187	0,009928	0,000023	0,002708	0,005011	0,002268	0,005809
TNBC_125	9	GPL570	GSM102545	1	1	65	1	12				-0,001079	0,003622	0,002183	0,007911	-0,001259	-0,010429	0,004013	-0,008271	0,005112
TNBC_126	9	GPL570	GSM102564	1	0	45	2	3				0,000648	0,005737	0,010897	0,009697	-0,000072	0,0048	0,004017	0,002328	0,007729
TNBC_127	9	GPL570	GSM117581	1	1	45	1	3				0,000529	0,005773	0,011748	0,011269	0,002925	0,006908	0,003239	0,005824	0,009253
TNBC_128	9	GPL570	GSM117661	1	1	55	2	3				-0,00035	0,005271	0,005809	0,011591	-0,007315	0,002555	0,001943	0,000995	0,007692
TNBC_129	9	GPL570	GSM117692	1	0	65	2	3				-0,002825	0,005107	0,013229	0,011355	0,002227	0,005677	0,003499	0,004862	0,007715
TNBC_130	9	GPL570	GSM138011	1		85						0,0003	0,008912	0,012214	0,012237	0,00374	0,009143	0,003382	0,005102	0,00912
TNBC_131	9	GPL570	GSM152698	1	1	75	2	3				-0,007471	0,004678	0,013247	0,011791	-0,001719	0,006755	0,006303	0,005561	0,007853
TNBC_132	9	GPL570	GSM179854	1								0,002974	0,006064	0,008875	0,01013	-0,000767	0,002463	0,001922	0,004763	0,007853
TNBC_133	9	GPL570	GSM179885	1								0,001527	0,005438	0,00997	0,009803	0,008563	0,006822	0,00148	0,002886	0,007036
TNBC_134	12	GPL96	GSM50035	1	1	61	2		22	1	YES	-0,002653	0,006286	0,006343	0,010957	-0,012168	0,004613	0,003857	0,00346	0,004267
TNBC_135	12	GPL96	GSM50040	1	0	57	2		91	0	YES	-0,00166	0,006722	0,005809	0,011785	-0,001494	0,006335	0,004388	0,004316	0,00675
TNBC_136	12	GPL96	GSM50041	1	0	73	2					-0,002765	0,004147	0,005558	0,008956	-0,007027	0,004665	0,004885	0,003024	0,005817
TNBC_137	12	GPL96	GSM50045	1	0	58	2		120	0	YES	-0,001914	0,005563	0,003619	0,009331	-0,004636	0,005264	0,005143	0,003469	0,003499
TNBC_138	12	GPL96	GSM50046	1	0	32	1		96	0	YES	-0,001614	0,004103	0,005581	0,008453	-0,008234	0,007796	0,0052	0,002818	0,006565
TNBC_139	12	GPL96	GSM50048	1	0	49	1		73	0	YES	0,000028	0,003264	0,004559	0,007344	-0,00484	0,005093	0,010439	0,001942	0,00363
TNBC_140	12	GPL96	GSM50050	1	0	63	1					-0,002687	0,004044	0,001413	0,009723	-0,003379	0,004349	0,003673	0,003878	0,005097
TNBC_141	12	GPL96	GSM50052	1	0	47	2		55	0	YES	-0,002669	0,004065	0,002265	0,008968	-0,007603	0,006703	-0,006951	0,001831	0,00031
TNBC_142	12	GPL96	GSM50055	1	0	33	2					-0,004152	0,003344	0,004069	0,008165	-0,005629	0,002452	0,002184	0,001589	0,003233
TNBC_143	12	GPL96	GSM50057	1	0	46	2					0,000501	0,004255	0,006452	0,007203	-0,008622	0,004033	0,000079	0,000083	0,006592
TNBC_144	12	GPL96	GSM50058	1	0	51	2					-0,003596	0,002564	-0,000118	0,009639	-0,009815	0,00283	0,00081	0,001209	0,000884
TNBC_145	12	GPL96	GSM50060	1	0	52	2		42	1	YES	0,001366	0,003635	0,007351	0,009455	-0,00235	0,001175	-0,000474	0,00123	0,003225
TNBC_146	12	GPL96	GSM50061	1	0	61	2		48	1	YES	-0,003908	0,003362	0,000259	0,008592	-0,011523	0,000661	-0,000016	0,00069	-0,000316
TNBC_147	12	GPL96	GSM50063	1	0	49	2		37	1	YES	-0,005149	0,00323	0,001127	0,008135	-0,007831	0,000884	0,003816	0,001554	0,003169
TNBC_148	12	GPL96	GSM50064	1	0	70	2		11	1	YES	-0,004504	0,003493	0,000375	0,00791	-0,005918	-0,001992	0,004398	-0,001501	0,005687
TNBC_149	12	GPL96	GSM50066	1	0	80	2		15	1	YES	-0,008191	0,003249	0,00261	0,008247	-0,015383	0,000916	0,003511	0,000472	0,000139
TNBC_150	12	GPL96	GSM50067	1	0	78	2		9	1		-0,006233	0,004998	0,007359	0,008595	-0,007469	0,010243	0,005152	0,004723	0,007195
TNBC_151	12	GPL96	GSM50069	1	0	73	2		46	1	YES	-0,002722	0,004114	0,002359	0,00974	-0,008348	0,002178	0,001442	0,000302	0,00239
TNBC_152	12	GPL96	GSM50070	1	1	58	2		40	1	YES	-0,00683	0,005498	0,006037	0,006632	-0,001332	0,003146	0,005276	0,001332	0,007284
TNBC_153	12	GPL96	GSM50071	1	1	78	2		70	0	NO	-0,003435	0,002438	0,002687	0,006538	-0,007868	-0,001884	0,002913	-0,002382	0,003075
TNBC_154	12	GPL96	GSM50073	1	1	50	2		87	0	YES	-0,007245	0,006985	0,00508	0,009121	-0,003897	0,005282	0,003027	0,004301	0,006639
TNBC_155	12	GPL96	GSM50075	1	1	49	2		88	0	YES	-0,007641	0,002556	-0,000312	0,008124	-0,010197	0,000227	0,003366	-0,000483	-0,000312
TNBC_156	12	GPL96	GSM50079	1	1	40	2		78	0	YES	-0,000482	0,002269	0,006523	0,00848	-0,004453	0,004963	0,003928	0,003063	0,003148
TNBC_157	12	GPL96	GSM50089	1	1	30	2		105	0		-0,008396	0,003559	-0,004502	0,007848	-0,009035	-0,012959	0,005106	-0,001886	0,002555
TNBC_158	12	GPL96	GSM50092	1	1	50	2		77	0	YES	-0,007707	0,00423	0,005187	0,007418	-0,003709	0,005071	0,003621	0,002289	0,0031

TNBC_159	12	GPL96	GSM50093	1	1	64	2		94	0	YES	-0,000233	0,004076	0,008151	0,006754	-0,004658	0,007452	0,00248	0,002882	0,00524
TNBC_160	12	GPL96	GSM50094	1	1	57	1		39	1	YES	-0,006716	0,004587	0,008272	0,007508	-0,007098	0,00273	0,005325	0,003112	0,006307
TNBC_161	12	GPL96	GSM50096	1	1	32	2		17	1	YES	-0,011093	0,0043	0,008886	0,008083	-0,005819	0,005245	0,005811	0,003325	0,005589
TNBC_162	12	GPL96	GSM50102	1	1	48	2		8	1	YES	-0,008367	0,004683	0,00594	0,008453	-0,00594	0,007139	0,005229	0,00277	0,00494
TNBC_163	12	GPL96	GSM50106	1	1	60	2		19	1	YES	-0,005255	0,004922	0,008008	0,01799	-0,006896	0,001474	0,004604	0,004838	0,002363
TNBC_164	12	GPL96	GSM50107	1	1	52	2		75	0	YES	-0,003992	0,00474	0,006598	0,009592	-0,013001	0,006515	0,006012	0,004131	0,003936
TNBC_165	12	GPL96	GSM50112	1	1	43	2		14	1	YES	-0,002857	0,006934	0,00713	0,008253	-0,011134	0,004517	0,003171	0,00127	0,005152
TNBC_166	12	GPL96	GSM50119	1	1	54	2		50	0	YES	-0,002475	0,004684	0,005402	0,008595	-0,00181	0,004737	0,002096	0,004151	0,001517
TNBC_167	12	GPL96	GSM50123	1	1	59	2		47	0	YES	-0,00269	0,004559	0,004474	0,00671	-0,01008	0,005635	0,006169	0,003709	0,001699
TNBC_168	12	GPL96	GSM50125	1	1	59	2					-0,002038	0,003857	0,008292	0,009201	-0,003581	0,004352	-0,00076	0,002782	0,006942
TNBC_169	14	GPL570	GSM151295	1	1	53	2		6	1	NO	-0,003487	0,003267	0,010537	0,010466	-0,010939	0,003226	0,003346	0,000617	0,006857
TNBC_170	14	GPL570	GSM151309	1	1	50	2	3	120	0	NO	-0,004555	0,00282	0,00883	0,007752	-0,004942	0,001411	0,00495	0,000294	0,00814
TNBC_171	17	GPL96	GSM36788	1	0	40	2	12	79	0	NO	-0,015837	0,004593	0,008679	0,008706	-0,003792	0,005501	0,003473	0,002777	0,002644
TNBC_172	17	GPL96	GSM36793	1	0	36	2	12	101	0	NO	-0,008815	0,00362	0,000221	0,006494	-0,004394	0,001686	0,006412	-0,00945	0,004034
TNBC_173	17	GPL96	GSM36795	1	0	71	2	12	88	0	NO	-0,011708	0,004245	0,004216	0,008888	-0,012221	0,001795	0,005224	0,000684	0,003789
TNBC_174	17	GPL96	GSM36797	1	0	44	1	3	9	1	NO	-0,017369	0,006143	0,007281	0,008949	-0,006302	0,006169	0,009055	0,000927	0,006831
TNBC_175	17	GPL96	GSM36798	1	0	41	1	3	106	0	NO	-0,019436	0,004388	0,005761	0,009664	-0,005842	0,003849	0,000583	-0,002342	0,003365
TNBC_176	17	GPL96	GSM36809	1	0	69	1	3	56	0	NO	-0,012939	0,004647	0,00615	0,007207	-0,001948	0,001419	0,009039	-0,000723	0,004007
TNBC_177	17	GPL96	GSM36822	1	0	73	2	3	50	0	NO	-0,004783	0,004438	0,007626	0,010151	-0,001461	0,002896	0,0062	0,000213	0,00186
TNBC_178	17	GPL96	GSM36824	1	0	70	2	3	86	0	NO	-0,010026	0,005756	0,007161	0,00818	-0,00829	0,003691	0,005432	0,002038	0,003223
TNBC_179	17	GPL96	GSM36828	1	0	51	2	3	76	0	NO	-0,010403	0,003643	0,005916	0,00711	-0,002914	0,005449	0,002338	0,00102	0,005129
TNBC_180	17	GPL96	GSM36835	1	0	68	2	3	14	1	NO	-0,017444	0,003794	0,006497	0,005269	0,001583	0,002839	0,004768	-0,003904	0,00273
TNBC_181	17	GPL96	GSM36846	1	0	36	1	3	103	0	NO	-0,012885	0,003792	0,00459	0,00821	-0,000342	0,003421	0,004614	-0,000399	-0,000314
TNBC_182	17	GPL96	GSM36855	1	0	34	2	3	120	0	NO	-0,013588	0,004625	0,007003	0,00682	-0,003841	0,006219	0,004739	0,000287	0,002012
TNBC_183	17	GPL96	GSM36862	1	0	42	2	3	25	1	NO	-0,016633	0,006705	0,003915	0,009033	-0,006909	0,00673	0,003052	0,00023	0,003327
TNBC_184	17	GPL96	GSM36876	1	0	42	2	3	120	0	NO	-0,015571	0,004273	0,003258	0,008547	-0,010123	0,008333	-0,000725	0,001709	0,004086
TNBC_185	17	GPL96	GSM36883	1	0	73	2	12	88	0	NO	-0,004985	0,002281	0,002338	0,006901	-0,009126	-0,000113	0,007212	-0,003464	0,005718
TNBC_186	17	GPL96	GSM36889	1	0	44	2	3	108	0	NO	-0,011478	0,002947	0,003227	0,008644	-0,007886	0,000449	0,005044	0,000477	0,002835
TNBC_187	17	GPL96	GSM36890	1	0	64	2	12	116	0	NO	-0,012861	0,002611	0,006348	0,008354	-0,004947	-0,000577	0,006651	-0,002061	0,004699
TNBC_188	17	GPL96	GSM36891	1	0	65	1	3	87	0	NO	-0,010956	0,004372	0,005683	0,007869	-0,000738	0,003388	0,006561	0,00071	0,00071
TNBC_189	17	GPL96	GSM36901	1	0	29	2	3	101	0	NO	-0,010558	0,005041	0,007173	0,008753	-0,001605	0,009455	0,002448	0,003662	0,003787
TNBC_190	17	GPL96	GSM36905	1	0	47	1	12	17	1	NO	-0,012097	0,004268	0,002645	0,006467	-0,007829	0,001074	0,003123	0,000393	0,002356
TNBC_191	17	GPL96	GSM36906	1	0	47	1	3	101	0	NO	-0,00986	0,004517	0,005833	0,008724	-0,00795	0,003691	0,003142	0,001652	0,00271
TNBC_192	17	GPL96	GSM36909	1	0	78	2	12	95	0	NO	-0,015275	0,003314	-0,000699	0,009528	-0,005437	-0,000311	0,003392	-0,001786	0,002511
TNBC_193	17	GPL96	GSM36912	1	0	53	2	3	97	0	NO	-0,004529	0,004794	0,006516	0,011177	-0,00498	0,006013	0,005358	0,002357	0,003099
TNBC_194	17	GPL96	GSM36923	1	0	46	2	3	23	1	NO	-0,007694	0,006587	0,005696	0,010231	-0,007343	0,00305	0,004337	0,000837	0,008126
TNBC_195	17	GPL96	GSM36927	1	0	50	2	3	8	1	NO	-0,013111	0,005853	0,004447	0,010172	-0,007284	0,011143	0,001861	-0,001022	0,004447
TNBC_196	17	GPL96	GSM36931	1	0	49	2	12	37	1	NO	-0,01382	0,00383	0,005732	0,00758	-0,006348	0,001152	0,008044	0,000455	0,002464
TNBC_197	17	GPL96	GSM36935	1	0	50	1	3	114	0	NO	-0,012642	0,00349	0,003243	0,009042	-0,008602	0,001924	0,004335	0,001676	0,003023
TNBC_198	17	GPL96	GSM36949	1	0	53	2	3	6	1	NO	-0,011119	0,003799	0,006038	0,00956	-0,00244	0,006314	0,004759	0,001233	0,004327
TNBC_199	17	GPL96	GSM36952	1	0	46	1	3	13	1	NO	-0,009405	0,005294	0,0029	0,0078	-0,007969	0,001267	0,001604	0,000056	0,001436
TNBC_200	17	GPL96	GSM36959	1	0	75	2	3	120	0	NO	-0,014366	0,003527	0,00599	0,009284	-0,004772	0,006846	0,002018	0,001556	0,004746
TNBC_201	17	GPL96	GSM36960	1	0	48	1	12	51	1	NO	-0,013055	0,003984	0,005276	0,009206	-0,001642	0,008425	0,005119	0,001157	-0,00035
TNBC_202	17	GPL96	GSM36961	1	0	68	1	12	120	0	NO	-0,010552	0,004232	0,004616	0,008326	-0,007639	0,001759	0,006792	0,00272	0,002336
TNBC_203	17	GPL96	GSM36966	1	0	70	2	3	90	0	NO	-0,011739	0,004304	0,006334	0,008975	-0,003375	0,005845	0,001857	0,001149	0,005625
TNBC_204	17	GPL96	GSM36969	1	0	41	1	3	32	1	NO	-0,008581	0,005704	0,006764	0,008682	-0,002625	0,006259	0,005205	0,000404	0,006234
TNBC_205	17	GPL96	GSM36977	1	0	72	2	3	120	0	NO	-0,011463	0,003632	0,005812	0,006243	-0,000726	0,007238	0,001634	0,000673	0,003121
TNBC_206	17	GPL96	GSM36981	1	0	60	2	3	87	0	NO	-0,011333	0,005107	0,008337	0,008467	-0,010578	0,007764	0,002986	-0,000156	0,004299
TNBC_207	17	GPL96	GSM36991	1	0	66	1	3	114	0	NO	-0,011745	0,005314	0,006655	0,008939	-0,007474	0,006779	0,004143	0,001887	0,00519
TNBC_208	17	GPL96	GSM37002	1	0	53	2	3	16	1	NO	-0,012127	0,00578	0,007565	0,009152	-0,00034	0,004448	0,007052	0,002323	0,004873
TNBC_209	17	GPL96	GSM37017	1	0	41	2	3	108	0	NO	-0,014779	0,003786	0,008199	0,007755	-0,007285	0,005353	0,005907	0,000966	0,004987
TNBC_210	17	GPL96	GSM37021	1	0	58	2	12	109	0	NO	-0,012832	0,006634	0,008093	0,0115	-0,013267	0,006531	0,001102	0,002254	0,006147
TNBC_211	17	GPL96	GSM37022	1	0	58	1	12	30	1	NO	-0,013942	0,004108	0,006265	0,009166	-0,008473	0,00362	0,004527	-0,000745	0,007908
TNBC_212	17	GPL96	GSM37044	1	0	74	2	3	112	0	NO	-0,011153	0,004105	0,003567	0,007954	-0,003453	-0,00133	0,006765	-0,002717	0,007983
TNBC_213	17	GPL96	GSM37045	1	0	43	1	3	120	0	NO	-0,013772	0,004371	0,009532	0,010243	-0,005451	0,006689	0,004424	0,002949	0,00603
TNBC_214	17	GPL96	GSM37047	1	0	44	1	3	108	0	NO	-0,013741	0,005257	0,006481	0,007928	0,000056	0,008206	0,001311	0,003004	0,003922
TNBC_215	17	GPL96	GSM37048	1	0	43	1	3	87	0	NO	-0,010162	0,002953	-0,000058	0,007383	-0,00275	0,001274	0,004075	0,000087	0,004198
TNBC_216	17	GPL96	GSM37050	1	0	69	1	12	18	1	NO	-0,013619	0,002779	0,002311	0,007016	-0,002229	0,000303	0,003933	-0,00033	0,000138
TNBC_217	17	GPL96	GSM37051	1	0	43	2	3	18	1	NO	-0,013319	0,001736	0,004422	0,008138	-0,01001	0,001736	0,003365	0,000543	0,002794
TNBC_218	17	GPL96	GSM120649	1	0	53	1	12	120	0	NO	-0,009927	0,005295	0,004662	0,009496	-0,001093	0,003424	0,00727	0,000317	0,001755
TNBC_219	17	GPL96	GSM120651	1	0	34	2	3	88	0	NO	-0,012538	0,004632	0,004219	0,008821	-0,007257	0,004042	0,006426	0,001269	0,005664
TNBC_220	17	GPL96	GSM120655	1	0	43	2	3	10	1	NO	-0,010987	0,003368	0,007205	0,009331	-0,004196	0,008034	0,005769	0,002071	0,005383
TNBC_221	17	GPL96	GSM120656	1	0	38	2	3	22	1	NO	-0,014511	0,004381	0,008652	0,007748	-0,006708	0,008104	0,00473	0,000301	0,003943
TNBC_222	17	GPL96	GSM120657	1	0	69	2	3	61	0	NO	-0,011201	0,004194	0,008415	0,007808	-0,004414	0,006566	0,004087	0,001076	0,005187
TNBC_223	17	GPL96	GSM120658	1	0	42	1	3	120	0	NO	-0,010049	0,004664	0,006477	0,009036	-0,009702	0,00725	0,004927	0,002772	0,002399
TNBC_224	17	GPL96	GSM120660	1	0	38	1	3	116	0	NO	-0,010935	0,00601	0,008292	0,008737	-0,002838	0,007206	0,003048	0,00256	0,004869
TNBC_225	17	GPL96	GSM120663	1	0	49	2	3	29	1	NO	-0,009382	0,003918	0,000276	0,007975	-0,008885	0,003587	-0,003267	0,001297	0,001932
TNBC_226	17	GPL96	GSM120666	1	0	34	2	12	87	0	NO	-0,010697	0,003443	0,003895	0,006887	-0,00525	0,004008	0,002708	-0,001637	0,002258
TNBC_227	17	GPL96	GSM120668	1	0	35	1	3	7	1	NO	-0,008653	0,004021	0,00771	0,009762	-0,001608	0,008708	0,004354	0,002024	0,003245
TNBC_228	17	GPL96	GSM120671	1	0	32	2	12	24	1	NO	-0,009566	0,00462	0,005136	0,010136	-0,004783	0,004457	0,00367	0,001848	0,001984
TNBC_229	17	GPL96	GSM120672	1	0	47	2	12	120	0	NO	-0,013445	0,003998	0,002382	0,007941	-0,003478	0,003259	0,003831	0,000822	0,001506
TNBC_230	17	GPL96	GSM120674	1	0	63	2	3	86	0	NO	-0,009532	0,005001	0,001437	0,008068	-0,011024	0,006134	0,001953	0,001243	0,006327
TNBC_231	17	GPL96	GSM120675	1	0	44	1	3	119	0	NO	-0,009999	0,005673	0,008398	0,00924	-0,003258	0,008314	0,00503	0,002415	0,00396
TNBC_232	17	GPL96	GSM120677	1	0	82	2	3	68	0	NO	-0,013321	0,004886	0,008025	0,008735	-0,004095	0,006661	0,002701	0,002184	0,005651
TNBC_233	17	GPL96	GSM120679	1	0	74	2	3	42	1	NO	-0,010236	0,005238	0,006575	0,009595	-0,002967	0,003341	0,003598	0,003234	0,005532
TNBC_234	17	GPL96	GSM120682	1	0	58	1	3	76	0	NO	-0,010938	0,005004	0,006399	0,006809	-0,003008	0,005551	0,001809	-0,000984	0,002379
TNBC_235	17	GPL96	GSM120683	1	0	63	1	3	120	0	NO	-0,00555	0,004445	0,009547	0,008522	-0,007811	0,002367	0,00298	0,001078	0,006733
TNBC_236	17	GPL96	GSM120684	1	0	44	2	12	120	0	NO	-0,010519	0,006986	0,007092	0,011395	-0,00348	0,008314	0,001798	0,000239	0,006827
TNBC_237	17	GPL96	GSM120685	1	0	57	1	3	88	0	NO	-0,00572	0,00478	0,007792	0,008593	-0,001161	0,009229	0,002643	0,002597	0,001713
TNBC_238	17	GPL96	GSM120686	1	0	66	1	3	81	0	NO	-0,011654	0,00609	0,004844	0,007059	-0,001107	0,001882	-0,001505	-0,000747	0,006976

TNBC_239	17	GPL96	GSM120687	1	0	41	2	12	9	1	NO	-0,011493	0,007451	0,008166	0,009266	-0,003107	0,008111	0,005163	0,003877	0,006571
TNBC_240	17	GPL96	GSM120688	1	0	44	2	3	120	0	NO	-0,01384	0,00738	0,009894	0,013165	-0,002757	0,009245	0,005295	0,002136	0,00392
TNBC_241	17	GPL96	GSM120690	1	0	58	2	3	88	0	NO	-0,010365	0,002101	0,005197	0,007213	0,000227	0,003976	0,001911	0,001306	0,003067
TNBC_242	17	GPL96	GSM120691	1	0	61	2	12	103	0	NO	-0,010725	0,004581	0,004087	0,009381	-0,007434	0,008531	0,000529	0,00384	-0,000713
TNBC_243	17	GPL96	GSM120692	1	0	39	2	3	89	0	NO	-0,01228	0,005775	0,008663	0,010429	-0,002944	0,006392	0,002917	0,002047	0,005747
TNBC_244	17	GPL96	GSM120695	1	0	38	2	12	90	0	NO	-0,013116	0,00555	0,005798	0,009636	-0,003948	0,00809	0,003314	0,002154	0,003507
TNBC_245	17	GPL96	GSM120696	1	0	33	2	3	120	0	NO	-0,01001	0,004393	0,007035	0,011011	-0,007702	0,008731	0,004131	0,003364	0,003726
TNBC_246	17	GPL96	GSM120698	1	0	57	2	12	61	0	NO	-0,012765	0,004801	0,004882	0,009983	-0,005537	0,012247	0,000745	0,001909	0,002919
TNBC_247	17	GPL96	GSM120699	1	0	40	1	3	80	0	NO	-0,009933	0,003919	0,005007	0,010178	-0,007675	0,01143	0,001511	0,004545	0,004327
TNBC_248	17	GPL96	GSM120701	1	0	50	2	3	69	0	NO	-0,0015	0,004732	0,006233	0,009032	-0,007647	0,006262	0,005959	0,001731	0,005713
TNBC_249	17	GPL96	GSM120702	1	0	39	2	3	4	1	NO	-0,008936	0,005395	0,007615	0,010622	-0,001433	0,008739	0,004708	0,00326	0,002754
TNBC_250	17	GPL96	GSM120703	1	0	59	2	12	91	0	NO	-0,008527	0,005065	0,009443	0,009243	-0,007726	0,002718	0,002452	0,002862	0,004321
TNBC_251	17	GPL96	GSM120705	1	0	73	2	3	83	0	NO	-0,009845	0,004196	0,006911	0,012094	-0,007377	0,008803	0,004113	0,002605	0,007569
TNBC_252	17	GPL96	GSM120706	1	0	46	1	3	102	0	NO	-0,004931	0,004536	0,003944	0,008903	-0,009692	0,006818	0,003863	0,003409	0,003663
TNBC_253	20	GPL96	GSM782569	2	0	65	2	12	11	1		-0,001842	0,003903	0,000165	0,008796	-0,008301	-0,001044	-0,003825	-0,003188	0,002556
TNBC_254	20	GPL96	GSM782570	2	1	32	2	3	39	1		-0,008208	0,006809	0,008262	0,011115	-0,004091	0,007885	0,004442	0,002315	0,008101
TNBC_255	20	GPL96	GSM782571	2	0	56	2	3	45	0		-0,007417	0,00619	0,007313	0,012301	-0,008409	0,005589	0,003695	0,002037	0,008618
TNBC_256	20	GPL96	GSM782572	2	0	55	2	12	45	0		-0,00165	0,004152	0,007338	0,010068	-0,004977	0,002873	0,006693	-0,000341	0,003527
TNBC_257	20	GPL96	GSM782573	2	1	44	2	12	23	1		-0,002231	0,004377	0,006354	0,009008	-0,004885	0,007794	0,003647	0,003982	0,006184
TNBC_258	20	GPL96	GSM782574	2	0	67	2	12	34	1		-0,002575	0,004011	0,00374	0,010137	-0,009838	0,001355	-0,00056	-0,000271	0,00187
TNBC_259	20	GPL96	GSM782575	2	1	46	2	3	15	1		-0,006389	0,005297	-0,000289	0,011397	-0,004334	-0,007448	0,00328	-0,002857	0,002279
TNBC_260	20	GPL96	GSM782576	2	0	60	2	3	51	0		-0,001939	0,003508	0,001141	0,009696	-0,001055	0,000114	0,002103	-0,00365	0,000371
TNBC_261	20	GPL96	GSM782577	2	1	63	2	12	44	0		-0,001593	0,005649	0,003679	0,009357	-0,00055	0,000463	0,005901	0,000009	0,003853
TNBC_262	20	GPL96	GSM782578	2	1	60	2	3	30	0		-0,003018	0,003865	0,003468	0,008126	-0,004129	0,000556	-0,001754	-0,000026	0,001429
TNBC_263	20	GPL96	GSM782579	2	1	44	2	12	27	0		-0,004094	0,002811	0,001228	0,009033	-0,009743	-0,001556	0,004389	-0,001119	0,004721
TNBC_264	20	GPL96	GSM782580	2	1	69	2	12	26	0		-0,001867	0,003544	0,004491	0,009929	-0,008928	0,006223	0,004742	0,002895	0,001596
TNBC_265	20	GPL96	GSM782581	2	1	50	2	3	25	0		0,000752	0,006152	0,0054	0,010611	-0,003251	0,007226	0,004209	0,002445	0,002257
TNBC_266	20	GPL96	GSM782582	2	1	44	2	3	10	1		-0,001051	0,005655	0,009459	0,010585	-0,00553	0,007657	0,005988	0,00548	0,006056
TNBC_267	20	GPL96	GSM782583	2								0,000407	0,004987	0,007989	0,009363	-0,004071	0,005597	0,004222	0,002442	0,008472
TNBC_268	20	GPL96	GSM782584	2	0	39	2	3	23	0		-0,001695	0,005377	0,005881	0,009987	-0,002861	0,010278	0,004273	0,004265	0,005324
TNBC_269	20	GPL96	GSM782585	2	0	41	2	3	23	0		-0,00238	0,006365	0,005776	0,011446	-0,005482	0,006418	0,003321	0,002621	0,006552
TNBC_270	20	GPL96	GSM782586	2	1	44	2	3	23	0		-0,001861	0,004262	0,006905	0,010358	-0,005611	0,004046	0,004197	0,003291	0,00348
TNBC_271	20	GPL96	GSM782587	2	1	42	2	12	24	0		-0,001545	0,005341	0,007409	0,009739	-0,003482	0,008377	0,006763	0,003979	0,004267
TNBC_272	21	GPL96	GSM505351	3	1	50	2	3				-0,007294	0,004713	0,005593	0,01061	-0,002088	0,004925	0,007875	0,003347	0,005895
TNBC_273	21	GPL96	GSM505331	3	0	75	2	3				-0,001028	0,005163	0,006483	0,009025	-0,004718	0,002464	0,006489	-0,000215	0,004741
TNBC_274	21	GPL96	GSM505460	3	0	52	2	3				-0,000194	0,00295	0,006699	0,00949	0,000147	0,002665	0,008029	0,002367	0,003157
TNBC_275	21	GPL96	GSM505345	3	0	65	1	3				-0,006452	0,005555	0,002732	0,006389	-0,007295	0,001818	0,005469	0,001235	-0,000097
TNBC_276	21	GPL96	GSM505369	3	0	59	2	3				-0,004468	0,004151	0,010117	0,007364	-0,001006	0,003561	0,009	0,002313	0,003716
TNBC_277	21	GPL96	GSM505343	3	1	66	2	3				-0,011771	0,004412	0,004325	0,008249	-0,001752	0,00245	0,00435	0,001907	0,00442
TNBC_278	21	GPL96	GSM505372	3	0	38	2	3				-0,003666	0,004688	0,008471	0,007897	-0,00301	0,006401	0,00101	-0,000689	0,005333
TNBC_279	21	GPL96	GSM505358	3	0	38	1	3				-0,005349	0,004853	0,011177	0,007368	-0,001648	0,006279	0,004856	0,003804	0,005699
TNBC_280	21	GPL96	GSM505336	3	1	61	1	3				-0,001809	0,005461	0,008121	0,010364	0,001991	0,007311	0,008758	0,003383	0,006312
TNBC_281	21	GPL96	GSM505333	3	1	29	2	3				-0,010363	0,006201	0,00566	0,008962	-0,005255	0,005708	0,003684	0,002549	0,006427
TNBC_282	21	GPL96	GSM505350	3	1	67	2	12				-0,000935	0,005883	0,005198	0,007258	-0,004492	-0,001072	0,00368	-0,002984	0,004445
TNBC_283	21	GPL96	GSM505370	3	1	35	1	3				-0,007656	0,00321	0,00505	0,008285	-0,001527	0,003076	0,006349	0,001771	0,005613
TNBC_284	21	GPL96	GSM505359	3	0	48	2	3				-0,003354	0,005021	0,006808	0,011976	-0,008787	0,001767	0,009015	0,001918	0,00534
TNBC_285	21	GPL96	GSM505360	3	1	52	2	3				-0,000979	0,005091	0,005144	0,008384	0,000533	0,000956	0,009301	-0,001124	0,004047
TNBC_286	21	GPL96	GSM505357	3	1	44	2	3				-0,003411	0,006398	0,007	0,010154	-0,002975	0,005504	0,007212	0,001225	0,005812
TNBC_287	21	GPL96	GSM505421	3	1	60	2	3				-0,011004	0,003714	0,003996	0,00889	0,007102	0,007585	0,004131	0,004545	0,00391
TNBC_288	21	GPL96	GSM505417	3	1	51	2	3				-0,007123	0,005757	0,007016	0,010938	-0,001922	0,008003	0,000675	0,002665	0,004138
TNBC_289	21	GPL96	GSM505423	3	1	48	2	3				-0,013755	0,005967	0,004084	0,008565	-0,00766	0,009815	0,00189	0,007342	0,00712
TNBC_290	21	GPL96	GSM505411	3	1	46	2	3				-0,003869	0,006785	0,002841	0,011854	-0,013335	0,006835	0,007191	0,002387	0,001531
TNBC_291	21	GPL96	GSM505413	3	1	51	2	12				-0,010095	0,004027	-0,003073	0,00708	-0,006083	0,004343	0,00421	0,002385	0,003404
TNBC_292	21	GPL96	GSM505435	3	1	71	1	12				-0,007605	0,002044	0,001943	0,007312	-0,010539	-0,002745	0,005699	0,002101	-0,000081
TNBC_293	21	GPL96	GSM505487	3	1	63	1	3				-0,003228	0,002335	0,00499	0,008233	-0,00069	0,00157	0,006457	0,002755	0,005789
TNBC_294	21	GPL96	GSM505459	3	1	62	2	3				0,000345	0,005177	0,010285	0,011012	-0,005515	0,003644	-0,004649	0,00283	0,003125
TNBC_295	21	GPL96	GSM505467	3	1	46	2	12				-0,00283	0,001936	0,000892	0,005542	0,00584	0,003396	0,006961	0,002148	0,003726
TNBC_296	21	GPL96	GSM505428	3	1	38	2	3				-0,010471	0,005553	0,008046	0,007225	-0,005394	0,002252	0,004909	0,002927	0,003894
TNBC_297	21	GPL96	GSM505449	3	1	41	2	3				0,001458	0,002937	0,004213	0,007966	-0,006897	0,002577	0,009101	0,002028	0,003859
TNBC_298	21	GPL96	GSM505473	3	1	62	2	3				0,001351	0,00426	0,008241	0,007925	-0,001742	0,000419	0,006266	0,003484	0,005044
TNBC_299	21	GPL96	GSM505462	3	1	50	2	12				-0,010694	0,002278	0,005926	0,009807	-0,012186	0,008551	0,004964	0,00687	0,003117
TNBC_300	21	GPL96	GSM505450	3	1	58	2	3				-0,006406	0,003932	0,004132	0,009724	-0,012189	0,003871	0,003792	0,001509	0,001653
TNBC_301	23	GPL570	GSM232199	1	0	73	1	3	120	0	NO	-0,002239	0,003882	0,006686	0,010121	-0,008681	0,001936	0,008964	0,000148	0,008316
TNBC_302	23	GPL570	GSM232204	1	0	82	1		120	0	NO	-0,001317	0,004182	0,00647	0,011102	-0,005246	-0,000183	0,009212	-0,002111	0,004207
TNBC_303	24	GPL96	GSM26871	2	0		2	3				-0,012641	0,004781	0,007847	0,009226	-0,00148	0,005497	0,00403	0,000788	0,002767
TNBC_304	24	GPL96	GSM26878	2	1		2	12				-0,012089	0,003723	0,007163	0,008346	-0,00507	0,001913	0,006057	-0,003501	0,00529
TNBC_305	24	GPL96	GSM26880	2	0		2	3				-0,015734	0,005135	0,009016	0,010254	-0,001803	0,001394	0,006813	-0,001601	0,005794
TNBC_306	24	GPL96	GSM26882	2	1		2	3				-0,016004	0,004879	0,008635	0,008612	-0,003981	0,005443	0,006907	0,00118	0,004302
TNBC_307	24	GPL96	GSM26883	2	0		2					-0,009524	0,003806	0,005344	0,01	-0,002174	0,002214	0,006771	-0,00074	0,002742
TNBC_308	24	GPL96	GSM26884	2	1		2	12				-0,011487	0,004735	0,008326	0,011231	-0,000527	0,005117	0,005775	-0,000168	0,002461
TNBC_309	24	GPL96	GSM26886	2	1		2	12				-0,011261	0,003996	0,006264	0,009254	-0,002144	0,001393	0,00654	-0,002757	0,006159
TNBC_310	24	GPL96	GSM26887	2	0		2	12				-0,011985	0,00488	0,011039	0,010532	-0,004165	0,003845	0,006426	0,001473	0,00736
TNBC_311	24	GPL96	GSM26888	2	0			3				-0,014421	0,003963	0,008037	0,010099	-0,003064	0,005483	0,005071	-0,000611	0,000632
TNBC_312	24	GPL96	GSM26889	2	1		2	12				-0,012665	0,005345	0,009918	0,008846	-0,001634	0,005488	0,008671	0,000073	0,004461
TNBC_313	24	GPL96	GSM26892	2	1		2	3				-0,01358	0,003215	0,009601	0,009101	-0,006752	0,008006	0,005803	0,002173	0,004044
TNBC_314	24	GPL96	GSM26893	2	1		2	3				-0,011149	0,005248	0,00736	0,010801	-0,004876	0,006842	0,006052	0,001965	0,006916
TNBC_315	24	GPL96	GSM26895	2	0		2	3				-0,010807	0,006101	0,011169	0,012083	-0,004188	0,00707	0,006149	0,000995	0,006521
TNBC_316	24	GPL96	GSM26898	2	1		2	3				-0,016339	0,006024	0,011299	0,012179	-0,004361	0,005374	0,004556	0,002095	0,007283
TNBC_317	24	GPL96	GSM26902	2	1		2	3				-0,013313	0,004341	0,008927	0,009985	-0,002241	0,007114	0,002392	0,001959	0,006218
TNBC_318	24	GPL96	GSM26903	2	0		2					-0,010458	0,004022	0,007483	0,008309	-0,000814	0,00077	0,005514	-0,001564	0,005991

TNBC_319	24	GPL96	GSM26905	2	1		2	12				-0,013214	0,004481	0,008533	0,009623	-0,007186	0,007315	0,006008	0,00241	0,004919
TNBC_320	24	GPL96	GSM26906	2	1		2	3				-0,009495	0,003668	0,0089	0,008308	-0,000603	0,004073	0,005217	-0,000621	0,002915
TNBC_321	24	GPL96	GSM26908	2	0		2	3				-0,012002	0,004826	0,009633	0,010405	-0,003748	0,005184	0,004976	0,002755	0,005145
TNBC_322	24	GPL96	GSM26910	2	1		2	3				-0,01183	0,00508	0,007035	0,006506	-0,005908	0,002784	0,004154	-0,005058	0,007626
TNBC_323	24	GPL96	GSM26912	2	0		2	3				-0,014198	0,004529	0,007633	0,009732	-0,007952	0,005655	0,002458	0,001542	0,004329
TNBC_324	25	GPL96	GSM282385	1	0	57	2	12	89	0	NO	-0,002659	0,004686	0,004096	0,013892	-0,002772	0,005196	0,006277	0,005496	0,006738
TNBC_325	25	GPL96	GSM282398	1	0	50	2	3	15	1	NO	-0,009787	0,003575	0,011766	0,010865	-0,009165	0,007203	-0,00017	0,001252	0,010868
TNBC_326	25	GPL96	GSM282413	1	0	68	1	3	17	1	NO	-0,010753	0,004426	0,007724	0,010353	0,000827	0,005282	0,0048	-0,001529	0,005398
TNBC_327	25	GPL96	GSM282427	1	0	56	2	3	120	0	NO	-0,003192	0,005851	0,005314	0,010209	-0,005153	0,004769	0,005878	0,003597	0,003162
TNBC_328	25	GPL96	GSM282435	1	0	40	2	3	115	0	NO	-0,005725	0,005213	0,009653	0,010603	-0,003713	0,004968	0,004513	0,002826	0,00388
TNBC_329	25	GPL96	GSM282440	1	0	52	2	3	106	0	NO	-0,001959	0,004688	0,00683	0,010026	-0,002495	0,006808	0,008229	0,005089	0,00493
TNBC_330	25	GPL96	GSM282446	1	0	67	1	12	114	0	NO	-0,004214	0,004424	0,003861	0,01034	0,000029	0,0031	0,003662	-0,00021	0,005595
TNBC_331	25	GPL96	GSM282454	1	0	43	1	3	8	1	NO	-0,012923	0,004722	0,007517	0,012154	-0,002782	0,008363	0,004315	0,002694	0,005352
TNBC_332	25	GPL96	GSM282457	1	0	54	1	3	120	0	NO	-0,003647	0,003521	0,003493	0,008677	0,005682	0,001496	0,005007	0,000184	0,006814
TNBC_333	25	GPL96	GSM282464	1	0	49	2	3	120	0	NO	-0,010668	0,004926	0,004372	0,011296	-0,002228	0,005289	0,004341	0,000748	0,005816
TNBC_334	25	GPL96	GSM282465	1	0	55	2	3	90	0	NO	-0,004828	0,002773	0,004057	0,008286	-0,005834	0,005189	0,006163	-0,001479	0,005459
TNBC_335	25	GPL96	GSM282474	1	0	45	2	3	120	0	NO	-0,004966	0,003871	0,00709	0,011986	-0,005471	0,006596	0,006148	0,002901	0,002599
TNBC_336	25	GPL96	GSM282482	1	0	58	2	12	116	1	NO	-0,003187	0,003052	0,003214	0,008036	-0,001207	0,001465	0,004348	-0,000213	0,00278
TNBC_337	25	GPL96	GSM282493	1	0	64	2	12	120	0	NO	-0,003258	0,004142	0,005068	0,009667	-0,006953	-0,000439	-0,000837	-0,000199	0,007721
TNBC_338	25	GPL96	GSM282497	1	0	65	2	3	93	0	NO	-0,004617	0,005038	0,00434	0,009687	-0,000617	0,005627	0,003174	0,001641	0,005708
TNBC_339	25	GPL96	GSM282511	1	0	58	2	3	120	0	NO	-0,00595	0,004199	0,005472	0,008348	0,000044	0,009881	0,005003	-0,00183	0,006127
TNBC_340	25	GPL96	GSM282528	1	0	69	1	12	120	0	NO	-0,004914	0,005005	0,004398	0,009803	-0,00155	0,006166	0,004047	0,000418	0,005917
TNBC_341	25	GPL96	GSM282535	1	0	34	1	3	37	1	NO	-0,005184	0,003446	-0,001837	0,007069	-0,001889	-0,00226	0,004814	-0,00195	0,002409
TNBC_342	25	GPL96	GSM282551	1	0	58	1	12	8	1	NO	-0,013272	0,006514	0,005898	0,010864	-0,002749	0,001833	0,005645	0,002429	0,004129
TNBC_343	25	GPL96	GSM282565	1	0	55	2	3	78	1	NO	-0,01289	0,00489	0,005624	0,012033	-0,001266	0,00336	0,005985	0,002574	0,006378
TNBC_344	25	GPL96	GSM282569	1	0	50	2	3	10	1	NO	-0,010953	0,004656	0,006778	0,009507	0,001666	0,007217	0,002029	0,000756	0,006992
TNBC_345	29	GPL96	ArrayExpress	1	1	72	2	12	104	0	YES	0,000607	0,003166	0,003991	0,007354	-0,003929	0,004081	0,004897	0,000974	0,009128
TNBC_346	29	GPL96	ArrayExpress	1	0	57	2	12	120	0	YES	-0,001341	0,004115	0,00663	0,008698	-0,002754	0,005368	0,005262	0,002058	0,005036
TNBC_347	29	GPL96	ArrayExpress	1	0	55	1	12	81	0	NO	-0,001658	0,003826	0,004243	0,008417	-0,013307	-0,001843	0,004847	-0,000723	0,006368
TNBC_348	29	GPL96	ArrayExpress	1	1	51	1	12	18	0	NO	-0,004148	0,003843	0,005553	0,008308	-0,011704	0,001446	0,003445	-0,000031	0,004039
TNBC_349	29	GPL96	ArrayExpress	1	0	50	1	12	43	1	YES	-0,002119	0,003307	0,007854	0,010223	-0,012405	0,004604	0,00301	0,002401	0,007845
TNBC_350	29	GPL96	ArrayExpress	1	0	45	1	12	10	1	NO	-0,002343	0,004327	0,009592	0,009188	-0,002538	0,002449	0,003254	-0,003269	0,004997
TNBC_351	29	GPL96	ArrayExpress	1	0	47	2	3	101	0	YES	-0,001642	0,00536	0,008342	0,010395	-0,004647	0,003013	-0,000059	0,001996	0,005164
TNBC_352	29	GPL96	ArrayExpress	1	0	60	2	3	37	1	YES	-0,001765	0,00373	0,007074	0,008159	-0,002046	0,002961	0,006039	-0,002091	0,005277
TNBC_353	29	GPL96	ArrayExpress	1	0	44	2		120	0	YES	-0,004861	0,003098	0,008005	0,009173	-0,00564	0,005766	0,009052	0,000009	0,00626
TNBC_354	29	GPL96	ArrayExpress	1	1	49	2	3	120	0	YES	-0,00224	0,004309	0,005975	0,007873	-0,005306	0,004427	0,000086	0,004445	0,00575
TNBC_355	29	GPL96	ArrayExpress	1	1	43	2	12	53	0	YES	-0,000236	0,003682	0,008585	0,009438	-0,003815	0,000617	0,003017	0,000203	0,006679
TNBC_356	29	GPL96	ArrayExpress	1	0	69	1	12	83	0	NO	-0,003319	0,001951	0,005468	0,006187	-0,007271	0,003028	-0,001819	0,00185	0,004143
TNBC_357	29	GPL96	ArrayExpress	1	0	44	2	3	120	0	YES	0,001055	0,003223	0,005992	0,007094	-0,002169	0,002242	0,000056	0,000918	0,008004
TNBC_358	29	GPL96	ArrayExpress	1	1	54	2	3	16	1	YES	-0,00123	0,003344	0,00542	0,007845	-0,005651	0,000682	0,004311	-0,001914	0,003927
TNBC_359	29	GPL96	ArrayExpress	1	1	40	2	12	117	0	YES	-0,001178	0,004571	0,008519	0,010676	-0,00377	0,005896	0,003478	0,001991	0,005492
TNBC_360	29	GPL96	ArrayExpress	1	1	39	1	3	4	1	YES	-0,004036	0,002938	0,004934	0,007651	-0,005265	0,001555	0,000397	-0,00086	0,005938
TNBC_361	29	GPL96	ArrayExpress	1	1	50	2	3	103	0	YES	0,000395	0,002951	0,006559	0,007594	-0,00124	0,004898	0,000554	0,001122	0,006151
TNBC_362	29	GPL96	ArrayExpress	1	0	36	1	12	120	0	NO	-0,002773	0,003195	0,007607	0,008101	-0,005226	0,003463	0,003435	0,001362	0,00523
TNBC_363	29	GPL96	ArrayExpress	1	1	74	1	3	98	0	YES	-0,002255	0,00341	0,006158	0,008997	-0,004468	0,002688	0,001335	0,001399	0,005315
TNBC_364	29	GPL96	ArrayExpress	1	1	50	2	12	119	0	YES	-0,002722	0,003915	0,004387	0,006621	0,00028	-0,001609	0,003432	-0,007597	0,006129
TNBC_365	29	GPL96	ArrayExpress	1	0	63	2		25	0	YES	0,000148	0,003989	0,007785	0,008537	-0,001697	0,007991	-0,001977	0,004346	0,007755
TNBC_366	29	GPL96	ArrayExpress	1	0	51	2	3	94	0	NO	-0,003047	0,005722	0,009297	0,010057	-0,005443	0,00917	0,004549	0,001077	0,009063
TNBC_367	29	GPL96	ArrayExpress	1	1	72	2		69	0	NO	-0,003484	0,00358	0,00758	0,008878	-0,006053	0,005895	0,003843	0,00294	0,004424
TNBC_368	30	GPL96	GSM125130	2							NO	-0,009358	0,003496	0,002541	0,011586	0,00062	-0,00324	0,003938	-0,001187	0,004554
TNBC_369	31	GPL96	GSM505513	3	1	42	2	3				-0,006853	0,005351	0,007706	0,008984	-0,007659	0,010569	0,00371	0,004718	0,005794
TNBC_370	31	GPL96	GSM505514	3	1	44	2	3				-0,014359	0,006155	0,007957	0,01095	-0,007083	0,002699	0,00584	0,000396	0,003708
TNBC_371	31	GPL96	GSM505516	3	1	39	2	3				-0,005584	0,005709	0,0095	0,009654	-0,000315	0,007974	0,005026	0,003016	0,004509
TNBC_372	31	GPL96	GSM505517	3	1	59	2	12				-0,009527	0,003289	-0,002003	0,006927	-0,002535	-0,007349	0,000804	-0,003067	-0,000196
TNBC_373	31	GPL96	GSM505526	3	1	68	2	12				-0,003536	0,004993	0,007391	0,007856	-0,003634	0,003678	0,004314	0,003722	0,007309
TNBC_374	31	GPL96	GSM505527	3	1	60	2	12				-0,003477	0,005877	0,004248	0,007689	-0,006349	0,003895	0,00129	0,001248	0,006345
TNBC_375	31	GPL96	GSM505528	3	1	52	2	3				-0,000282	0,002986	0,004632	0,008696	-0,004732	0,001074	0,002399	0,000826	0,004665
TNBC_376	31	GPL96	GSM505531	3	1	46	2	3				-0,006402	0,002258	0,002812	0,006488	-0,00618	-0,00282	0,00455	0,001713	0,002017
TNBC_377	31	GPL96	GSM505533	3	0	50	2	12				-0,020329	0,005053	-0,004048	0,006465	-0,000566	-0,007402	0,003046	-0,004977	0,000604
TNBC_378	31	GPL96	GSM505534	3	0	66	2	12				-0,006187	0,007927	0,007905	0,011395	-0,003893	0,008448	0,006156	0,003107	0,004954
TNBC_379	31	GPL96	GSM505541	3	1	62	2	3				-0,011948	0,005303	0,003661	0,009008	-0,005547	0,002823	0,005066	0,000218	0,004742
TNBC_380	31	GPL96	GSM505543	3	1	68	2	3				-0,003786	0,005601	0,012238	0,012171	-0,005278	0,005619	0,003031	0,001328	0,003419
TNBC_381	31	GPL96	GSM505545	3	1	32	2	3				-0,009891	0,006006	0,006523	0,00732	-0,000924	0,005125	0,007447	0,003887	0,00456
TNBC_382	31	GPL96	GSM505548	3	1	51	2	12				-0,015395	0,004028	-0,00205	0,006604	0,000904	-0,001646	0,00632	-0,002532	0,001858
TNBC_383	31	GPL96	GSM505552	3	1	40	2	3				-0,010686	0,003684	0,003258	0,008943	-0,003185	-0,001423	0,003042	0,000482	0,005078
TNBC_384	31	GPL96	GSM505553	3	1	50	2	3				-0,001586	0,005492	0,006318	0,010128	-0,010247	0,00566	0,001746	0,003317	0,005625
TNBC_385	31	GPL96	GSM505554	3	1	52	2	3				-0,011798	0,003199	0,004392	0,008197	-0,007157	0,002006	0,008244	0,000634	0,001035
TNBC_386	31	GPL96	GSM505561	3	0	48	1	12				-0,009859	0,005342	0,005026	0,010261	-0,005094	0,003683	0,005296	0,001304	0,002548
TNBC_387	31	GPL96	GSM505562	3	1	61	2	3				-0,009539	0,007281	0,009575	0,012511	-0,007139	0,006689	0,006952	0,003632	0,006823
TNBC_388	31	GPL96	GSM505563	3	1	51	2	3				-0,004176	0,004107	0,003455	0,006524	-0,00707	0,002675	0,004908	-0,000631	0,002619
TNBC_389	31	GPL96	GSM505564	3	1	49	2	3				-0,006379	0,005388	0,006293	0,011272	-0,005676	0,007431	0,004953	0,003595	0,00608
TNBC_390	31	GPL96	GSM505566	3	1	57	2	3				0,001184	0,00453	0,005518	0,008998	-0,00181	0,003977	0,005402	0,004209	0,000272
TNBC_391	31	GPL96	GSM505568	3	1	50	2	3				-0,001651	0,004619	0,000325	0,008841	-0,007155	-0,000136	0,002577	-0,001558	0,003314
TNBC_392	31	GPL96	GSM505570	3	1	43	2	12				-0,011749	0,003661	0,006614	0,009544	-0,000913	0,002019	0,006289	-0,004646	0,0016
TNBC_393	31	GPL96	GSM505572	3	1	58	2	3				-0,000946	0,004473	0,000495	0,008265	-0,010839	-0,005947	0,004986	-0,001225	0,00174
TNBC_394	31	GPL96	GSM505574	3	0	72	2	3				-0,005374	0,0047	0,008468	0,00846	-0,001598	0,004289	0,005346	0,001852	0,002134
TNBC_395	31	GPL96	GSM505575	3	0	33	2	3				-0,005589	0,007039	0,007981	0,009768	-0,004626	0,001753	0,005467	0,003814	0,005292
TNBC_396	31	GPL96	GSM505580	3	1	46	2	12				-0,009221	0,003941	0,00151	0,007976	-0,000208	0,000999	0,005687	-0,000723	0,004554
TNBC_397	31	GPL96	GSM505583	3	1	53	2	3				-0,009498	0,006911	0,009128	0,009865	-0,002429	0,002595	0,000907	0,003216	0,006497
TNBC_398	31	GPL96	GSM505584	3	1	51	2	3				-0,002536	0,005775	0,002877	0,009681	-0,00633	0,001679	0,005063	0,000487	0,002779

TNBC_399	31	GPL96	GSM505586	3	1	64	2	3				-0,010139	0,005308	0,000697	0,009581	-0,004627	-0,004682	0,001345	-0,001417	0,005077
TNBC_400	31	GPL96	GSM505588	3	0	36	2	3				-0,010872	0,004557	0,007015	0,00546	-0,001548	0,003694	0,007135	-0,00334	0,004603
TNBC_401	31	GPL96	GSM505592	3	1	50	2	3				-0,01001	0,004833	0,010031	0,008828	-0,004358	0,00476	0,000471	0,00355	0,007311
TNBC_402	32	GPL570	GSM85474	1				3				-0,010896	0,005632	0,011748	0,012112	-0,001599	0,006335	0,006864	0,002627	0,002078
TNBC_403	32	GPL570	GSM85476	1				3				-0,004493	0,001562	0,004916	0,007756	-0,003754	0,002844	0,003774	-0,000308	0,005166
TNBC_404	32	GPL570	GSM85477	1				3				-0,000416	0,003492	0,006812	0,010118	-0,002242	0,001886	0,005127	0,002567	0,004176
TNBC_405	32	GPL570	GSM85478	1				3				0,000805	0,006234	0,006743	0,009405	-0,0016	0,008071	0,006249	0,002589	0,006216
TNBC_406	32	GPL570	GSM85479	1				3				0,002555	0,005197	0,008225	0,009698	-0,000518	0,005311	0,00152	0,00436	0,005563
TNBC_407	32	GPL570	GSM85480	1				3				-0,001887	0,000768	0,003284	0,00804	0,002801	-0,000031	0,002991	-0,002063	0,00007
TNBC_408	32	GPL570	GSM85481	1				3				0,001117	0,00676	0,007967	0,012113	-0,003305	0,006109	0,006566	0,003648	0,008579
TNBC_409	32	GPL570	GSM85482	1				3				-0,010145	0,004099	0,002506	0,008949	-0,000798	0,004781	0,005264	-0,000313	0,005665
TNBC_410	32	GPL570	GSM85483	1				3				-0,0037	0,003679	0,0069	0,0084	0,002542	0,003534	0,003392	0,002174	0,004143
TNBC_411	32	GPL570	GSM85484	1				3				-0,011184	0,004003	0,006263	0,01854	-0,012382	0,006464	0,008349	0,001565	0,005189
TNBC_412	32	GPL570	GSM85485	1				3				0,000102	0,004409	0,009694	0,009383	-0,007503	0,003157	0,006011	0,003133	0,004957
TNBC_413	32	GPL570	GSM85486	1				3				-0,001055	0,003845	0,005994	0,008524	-0,010934	0,008942	0,00558	0,000993	0,006422
TNBC_414	32	GPL570	GSM85487	1				3				-0,00152	0,006906	0,0119	0,009829	-0,00002	0,006507	0,004076	0,004249	0,0061
TNBC_415	32	GPL570	GSM85489	1				3				0,000891	0,002542	0,004694	0,009439	-0,001036	0,007444	0,005945	0,002679	0,002984
TNBC_416	32	GPL570	GSM85490	1				3				0,00836	0,00246	0,006975	0,009734	-0,002056	0,003801	0,006906	0,002223	0,005185
TNBC_417	32	GPL570	GSM85492	1				3				-0,003517	0,002242	0,006585	0,006373	-0,002722	0,002105	0,004533	-0,002066	0,006819
TNBC_418	33	GPL96	GSM782588	1	0	59	1	12	56	0	NO	-0,008405	0,001877	-0,004131	0,007916	0,001222	-0,00886	0,003803	-0,00261	0,006017
TNBC_419	33	GPL96	GSM782589	1	1	62	2	3	66	1	NO	-0,004401	0,002636	0,002582	0,008455	-0,00518	0,000247	0,004338	0,000646	0,004137
TNBC_420	34	GPL96	GSM305166	1	0				101	0	NO	-0,006139	0,004316	0,006315	0,010626	-0,002847	0,001339	0,006308	0,000195	0,003807
TNBC_421	35	GPL96	GSM121673	1								-0,002802	0,006544	0,007454	0,008464	-0,002967	0,006042	0,005604	0,002787	0,007862
TNBC_422	35	GPL96	GSM121675	1								-0,012883	0,005755	0,006155	0,008843	-0,00211	0,002506	0,007441	0,002117	0,007499
TNBC_423	35	GPL96	GSM121678	1								-0,004554	0,004487	0,002169	0,007901	-0,000831	0,004759	0,005991	-0,0022	0,004224
TNBC_424	35	GPL96	GSM121684	1								-0,004263	0,005854	0,006619	0,007467	-0,001764	0,002636	0,00649	-0,000222	0,006012
TNBC_425	35	GPL96	GSM121685	1								-0,012583	0,006466	0,00388	0,006579	-0,001662	0,001289	0,00718	-0,001014	0,006146
TNBC_426	35	GPL96	GSM121690	1								-0,002376	0,004218	0,004001	0,006777	-0,008434	-0,000007	-0,001465	-0,000122	0,004143
TNBC_427	35	GPL96	GSM121692	1								-0,007909	0,004994	0,003576	0,008089	-0,000224	0,001085	0,005694	0,00045	0,004335
TNBC_428	35	GPL96	GSM121693	1								-0,004763	0,004747	0,005338	0,008222	-0,001514	0,002867	0,00719	-0,00155	0,003214
TNBC_429	35	GPL96	GSM121697	1								-0,002554	0,004632	0,001333	0,007773	-0,004226	0,001341	0,001347	-0,003796	0,008535
TNBC_430	35	GPL96	GSM121700	1								-0,004023	0,005184	0,002796	0,009276	-0,002341	0,005432	0,002501	-0,001184	0,002975
TNBC_431	35	GPL96	GSM121703	1								-0,013726	0,004797	0,002679	0,006868	-0,001939	-0,000071	0,004192	-0,001536	0,005961
TNBC_432	35	GPL96	GSM121708	1								-0,005145	0,005298	0,002719	0,00818	0,00449	0,004181	0,005387	0,002438	0,003196
TNBC_433	35	GPL96	GSM121709	1								-0,009095	0,004419	0,002128	0,009292	-0,001491	0,005798	0,006989	0,003821	0,005331
TNBC_434	35	GPL96	GSM121721	1								-0,00502	0,005885	0,003076	0,00666	-0,00083	0,002367	0,004014	-0,003045	0,004163
TNBC_435	35	GPL96	GSM121724	1								-0,015329	0,004693	0,000979	0,006432	-0,002614	0,003726	0,0064	-0,00011	0,004997
TNBC_436	35	GPL96	GSM121758	1								-0,010633	0,005007	0,001148	0,007451	-0,004959	-0,001013	0,002593	-0,004656	0,004532
TNBC_437	35	GPL96	GSM121763	1								-0,003611	0,003878	0,002745	0,007713	-0,001119	0,006107	0,003661	-0,000002	0,002299
TNBC_438	35	GPL96	GSM121764	1								-0,006629	0,005937	0,004676	0,010372	0,001213	0,0025	0,00724	0,001958	0,002653
TNBC_439	35	GPL96	GSM121765	1								-0,001823	0,00507	-0,001699	0,009202	-0,001868	-0,000151	0,004564	-0,005572	0,007399
TNBC_440	35	GPL96	GSM121768	1								-0,004792	0,00577	0,003101	0,008942	-0,001964	0,002406	0,005557	-0,000555	0,005171
TNBC_441	35	GPL96	GSM121772	1								-0,003657	0,004603	0,005285	0,009129	-0,007935	0,007049	0,002802	0,002046	0,007087
TNBC_442	35	GPL96	GSM121777	1								-0,003131	0,005041	0,005463	0,008723	-0,011383	0,002967	0,004233	-0,000057	0,004531
TNBC_443	35	GPL96	GSM121781	1								-0,001115	0,005198	0,004238	0,009211	-0,005339	0,004235	-0,001087	-0,000159	0,008428
TNBC_444	35	GPL96	GSM121800	1								-0,008197	0,003918	0,004193	0,00822	-0,007554	0,003874	0,004303	0,003176	0,004727
TNBC_445	35	GPL96	GSM121807	1								-0,005118	0,006203	0,003201	0,006314	-0,007464	0,003459	0,00808	0,000605	0,002002
TNBC_446	35	GPL96	GSM121812	1								-0,004232	0,004995	0,002326	0,007516	-0,002992	0,004623	0,007125	0,002161	0,003438
TNBC_447	35	GPL96	GSM121818	1								-0,008949	0,005446	0,001736	0,007191	-0,002662	0,002445	-0,000137	-0,000093	0,005706
TNBC_448	35	GPL96	GSM121822	1								-0,013579	0,007571	0,010503	0,008171	-0,003591	0,006793	0,004227	0,005877	0,005319
TNBC_449	35	GPL96	GSM121824	1								-0,004191	0,007726	0,004476	0,008027	-0,008316	0,004126	0,004157	0,002318	0,004074
TNBC_450	35	GPL96	GSM121825	1								-0,009072	0,006408	0,004062	0,010716	-0,003739	0,002511	0,003218	0,001486	0,010131
TNBC_451	35	GPL96	GSM121833	1								-0,007093	0,004704	0,006655	0,006583	-0,004897	0,004281	0,005578	0,00206	0,004818
TNBC_452	35	GPL96	GSM121834	1								-0,009825	0,005762	0,000192	0,009128	-0,001573	0,004929	0,002678	-0,00135	0,003753
TNBC_453	35	GPL96	GSM121836	1								-0,002181	0,007768	0,006675	0,009395	-0,009879	0,005951	0,004524	0,002513	0,001721
TNBC_454	35	GPL96	GSM121848	1								-0,00991	0,005766	0,004841	0,007539	-0,000331	0,004797	0,007737	0,001303	0,003307
TNBC_455	35	GPL96	GSM121851	1								-0,003653	0,00468	0,001814	0,007596	-0,004545	-0,00098	0,003294	-0,002774	0,006346
TNBC_456	35	GPL96	GSM121852	1								-0,006984	0,004271	0,001078	0,004262	-0,008067	-0,000205	0,007468	-0,002418	0,003505
TNBC_457	36	GPL570	GSM320216	1								0,000901	0,004162	0,0092	0,006462	-0,003127	0,004934	0,003463	0,001974	0,008139
TNBC_458	36	GPL570	GSM320228	1								-0,000872	0,003076	0,006473	0,008823	-0,000414	0,009805	0,006239	0,002569	0,005129
TNBC_459	36	GPL570	GSM320230	1								-0,000914	0,003878	0,006607	0,009528	-0,002749	0,007237	0,001577	0,000282	0,003699
TNBC_460	36	GPL570	GSM320234	1								-0,001799	0,004942	0,009193	0,009233	-0,001863	0,003898	0,006509	0,001418	0,005129
TNBC_461	36	GPL570	GSM320237	1								0,000173	0,005492	0,008343	0,009081	-0,004007	0,004117	0,004711	0,002945	0,007039
TNBC_462	37	GPL96	GSM152339	1		56	1	12				-0,012466	0,004334	0,006634	0,009272	-0,007999	0,003251	0,005579	-0,001021	0,004851
TNBC_463	37	GPL96	GSM152343	1		85	2	3				-0,013908	0,006028	0,008928	0,009503	-0,0104	0,003592	0,005437	0,000061	0,004176
TNBC_464	37	GPL96	GSM152344	1		29	2	12				-0,009875	0,00695	0,011318	0,010872	-0,000808	0,002573	0,009854	0,001523	0,006268
TNBC_465	37	GPL96	GSM152349	1		65	2	3				-0,011063	0,005754	0,007993	0,009022	-0,004825	0,007416	0,005085	0,005884	0,007335
TNBC_466	37	GPL96	GSM152352	1		65	2	3				-0,011535	0,003917	0,008728	0,006258	-0,005031	0,005459	0,003971	0,001617	0,005815
TNBC_467	37	GPL96	GSM152357	1		41	2	3				-0,015677	0,007834	0,008941	0,008696	0,001083	0,00325	0,004241	0,000012	0,004166
TNBC_468	37	GPL96	GSM152361	1		51	1	3				-0,007559	0,007291	0,009481	0,008688	-0,005162	0,00009	0,003041	0,00021	0,004465
TNBC_469	38	GPL570	GSM346882	1				3				-0,002692	0,003964	0,009988	0,008576	-0,002207	0,007637	0,004094	0,001464	0,007733
TNBC_470	38	GPL570	GSM346883	1				3				-0,000225	0,001344	0,007493	0,007898	-0,001315	0,004227	0,003546	0,000483	0,008288
TNBC_471	38	GPL570	GSM346884	1				3				-0,001878	0,004236	0,010258	0,010338	0,000544	0,00925	0,00631	0,002445	0,007072
TNBC_472	38	GPL570	GSM346885	1				3				-0,001322	-0,000252	0,00713	0,006412	0,000315	0,003476	0,005973	0,001784	0,00519
TNBC_473	38	GPL570	GSM346886	1				3				-0,000574	0,002663	0,00555	0,008686	-0,003309	0,004677	0,00409	0,002136	0,004654
TNBC_474	38	GPL570	GSM346889	1				3				-0,001025	0,004186	0,007441	0,008205	-0,002901	0,003733	0,00514	0,00108	0,007746
TNBC_475	38	GPL570	GSM346890	1				3				-0,001376	0,00269	0,007676	0,008088	0,000478	0,00638	0,005015	0,000715	0,007403
TNBC_476	38	GPL570	GSM346892	1				3				-0,000844	0,002815	0,005518	0,006195	-0,000553	0,00295	0,005248	-0,000524	0,005821
TNBC_477	38	GPL570	GSM346900	1				3				-0,002024	0,004252	0,007773	0,007726	-0,002761	0,003249	0,006992	0,001161	0,007842
TNBC_478	38	GPL570	GSM346904	1				3				-0,004837	0,003007	0,009784	0,008232	-0,001898	0,007495	0,00337	0,002986	0,006715

TNBC_479	39	GPL570	GSM272166	4								-0,000841	0,004666	0,005912	0,009633	-0,000467	0,004457	0,004082	-0,001074	0,008837
TNBC_480	39	GPL570	GSM272167	4								-0,001285	0,004199	0,005528	0,009852	-0,000782	0,002798	-0,001897	0,00068	0,008185
TNBC_481	39	GPL570	GSM272221	4								-0,000911	0,005629	0,010301	0,010406	-0,004734	0,005201	0,006741	0,003935	0,005026
TNBC_482	39	GPL570	GSM272242	4								-0,00464	0,005223	0,004565	0,007674	-0,009002	0,002129	0,005015	-0,009644	0,007416
TNBC_483	39	GPL570	GSM272287	4								0,000345	0,003057	0,001889	0,008804	-0,003547	-0,008929	0,004757	-0,005365	0,003493
TNBC_484	40	GPL96	GSM177925	1	0	52	2	3	120	0	NO	-0,001899	0,003218	0,009205	0,008493	-0,000501	0,003561	-0,000174	0,003719	0,003956
TNBC_485	40	GPL96	GSM177935	1	0	37	2	3	14	1	NO	0,000714	0,00232	0,008618	0,008745	0,001377	0,005889	0,00022	0,00283	0,006654
TNBC_486	40	GPL96	GSM177937	1	0	53	2	3	103	1	NO	-0,001085	0,003074	0,006795	0,00819	-0,004289	0,002868	0,002577	0,003462	0,003953
TNBC_487	40	GPL96	GSM177943	1	0	39	1	12	108	0	NO	-0,000787	0,003453	0,008733	0,010078	-0,001041	0,004544	0,004144	0,000457	0,002996
TNBC_488	40	GPL96	GSM177944	1	0	52	2	12	18	1	NO	-0,002145	0,004881	0,004363	0,009614	-0,000271	0,005769	0,004779	0,001208	0,003155
TNBC_489	40	GPL96	GSM177954	1	0	37	2	12	26	1	NO	-0,00068	0,001779	0,008947	0,01036	-0,002119	0,006252	0,005356	0,003061	0,006435
TNBC_490	40	GPL96	GSM177956	1	0	40	2	3	17	1	NO	-0,002908	0,002934	0,006935	0,009496	-0,002908	0,001867	0,005941	-0,00008	0,004455
TNBC_491	40	GPL96	GSM177963	1	0	45	2	3	57	0	NO	-0,001263	0,003192	0,012125	0,010815	-0,001715	0,000929	0,005821	0,002239	0,005169
TNBC_492	40	GPL96	GSM177966	1	0	40	2	3	51	0	NO	0,000256	0,002894	0,007889	0,008273	-0,001767	0,004867	0,004265	0,001178	0,00794
TNBC_493	40	GPL96	GSM177970	1	0	47	1	3	108	0	NO	-0,000368	0,003078	0,006787	0,010655	-0,006156	0,005682	0,005064	0,003025	0,006472
TNBC_494	40	GPL96	GSM177885	1	0	57	2	3	24	1	NO	-0,001977	0,004084	0,005411	0,009521	-0,002445	-0,000572	0,004746	-0,000156	0,008116
TNBC_495	40	GPL96	GSM177897	1	0	46	2	3	120	0	NO	-0,002904	0,002114	0,005272	0,00866	-0,000968	0,00433	0,00508	0,003235	0,006597
TNBC_496	40	GPL96	GSM177898	1	0	57	2	12	93	1	NO	0,000304	0,002054	0,002156	0,008928	-0,002409	-0,001471	0,005269	-0,000659	0,006468
TNBC_497	40	GPL96	GSM177899	1	0	33	2	3	15	1	NO	-0,001241	0,004185	0,012069	0,008176	-0,000852	0,002628	0,004973	0,001703	0,006594
TNBC_498	40	GPL96	GSM177903	1	0	57	2	12	60	1	NO	-0,00046	0,004654	0,004245	0,010127	-0,001611	0,000332	0,003341	0,001841	0,007135
TNBC_499	40	GPL96	GSM177909	1	0	58	2	3	4	1	NO	-0,009463	0,002751	0,007419	0,010246	0,000429	0,001539	0,004812	0,00217	0,005173
TNBC_500	40	GPL96	GSM177913	1	0	47	2	3	120	0	NO	-0,00278	0,004004	0,004641	0,009078	-0,0038	0,004488	0,007515	0,001505	0,008849
TNBC_501	40	GPL96	GSM177988	1	0	43	2	3	88	0	NO	-0,003509	0,00423	0,005972	0,007465	-0,004778	-0,000423	0,003848	0,000995	0,006096
TNBC_502	40	GPL96	GSM177999	1	0	43	2	12	13	1	NO	-0,001505	0,003476	0,005577	0,008924	-0,002594	0,00192	0,007944	0,00096	0,008275
TNBC_503	40	GPL96	GSM178003	1	0	43	2	3	95	1	NO	-0,003925	0,00165	0,006174	0,007799	-0,000975	0,0034	0,004062	0,00085	0,00355
TNBC_504	40	GPL96	GSM178009	1	0	53	2	3	9	1	NO	-0,002771	0,004593	0,000499	0,011733	-0,003021	0,003245	0,005715	0,000649	0,005293
TNBC_505	40	GPL96	GSM178012	1	0	38	2	3	120	0	NO	-0,005447	0,003065	0,009804	0,009424	-0,003116	0,00722	0,002607	0,001951	0,005573
TNBC_506	40	GPL96	GSM178021	1	0	33	2	3	120	0	NO	-0,011573	0,004835	0,008075	0,008898	-0,002417	0,008512	0,003149	0,0009	0,006506
TNBC_507	40	GPL96	GSM178023	1	0	42	2	3	120	0	NO	-0,002229	0,003671	0,008155	0,005979	-0,005612	0,007185	0,007233	0,001573	0,005717
TNBC_508	40	GPL96	GSM178058	1	0	46	2	3	120	0	NO	0,000892	0,005527	0,009766	0,009171	-0,008477	0,003098	0,00255	0,001264	0,007014
TNBC_509	40	GPL96	GSM178065	1	0	47	2	3	120	0	NO	-0,001428	0,002698	0,008068	0,007936	-0,005449	0,002566	0,006659	0,001746	0,007221
TNBC_510	40	GPL96	GSM178075	1	0	48	2	3	95	0	NO	-0,008118	0,002908	0,00354	0,010495	-0,002908	0,002883	0,005175	0,001644	0,003869
TNBC_511	40	GPL96	GSM178078	1	0	39	2	3	120	0	NO	0,001089	0,004677	0,006582	0,01168	-0,001287	0,005221	0,002076	0,003019	0,00636
TNBC_512	40	GPL96	GSM178079	1	0	46	2	3	13	1	NO	-0,000712	0,006436	0,010219	0,010956	-0,002579	0,005183	0,006214	0,002825	0,008524
TNBC_513	40	GPL96	GSM178082	1	0	39	2	3	44	1	NO	-0,00265	0,003368	0,004086	0,010128	-0,004061	0,001634	0,005974	0,00057	0,008221
TNBC_514	40	GPL96	GSM177978	1	0	47	2	3	120	0	NO	-0,007929	0,003027	0,008669	0,007388	-0,006029	0,00522	-0,002118	0,003254	0,005841
TNBC_515	40	GPL96	GSM177979	1	0	39	2	3	84	1	NO	-0,007881	0,003311	0,005988	0,007753	-0,008484	0,004631	0,007288	0,000765	0,007333
TNBC_516	40	GPL96	GSM177980	1	0	32	2	3	120	0	NO	-0,011799	0,004297	0,008047	0,010424	-0,004283	0,007797	0,007105	0,001761	0,007856
TNBC_517	40	GPL96	GSM177985	1	0	45	2	3	120	0	NO	-0,001154	0,002497	0,005299	0,008348	-0,002216	0,002025	0,005765	0,001473	0,00539
TNBC_518	40	GPL96	GSM177993	1	0	38	1	12	9	1	NO	-0,003805	0,002546	0,004076	0,008621	0,003859	-0,00104	0,00513	-0,001224	0,006873
TNBC_519	40	GPL96	GSM177994	1	0	47	1	12	42	0	NO	-0,011318	0,003849	0,001937	0,008508	-0,001952	0,00047	0,005967	0,001054	0,005043
TNBC_520	40	GPL96	GSM178013	1	0	40	1	3	120	0	NO	-0,004578	0,003131	0,010223	0,007319	-0,000069	0,00857	0,009869	0,00408	0,009489
TNBC_521	40	GPL96	GSM178016	1	0	40	2	12	120	0	NO	-0,002087	0,001624	0,002933	0,008477	-0,004342	-0,00054	0,005074	-0,002393	0,003403
TNBC_522	40	GPL96	GSM178034	1	0	32	1	12	120	0	NO	-0,00525	0,005698	0,009656	0,00895	-0,00753	0,008101	0,006396	0,001804	0,004934
TNBC_523	40	GPL96	GSM178035	1	0	50	1	3	120	0	NO	-0,002681	0,0028	0,006097	0,009177	-0,004681	0,004464	0,005439	0,00172	0,005734
TNBC_524	42	GPL570	GSM308259	1		61	2	3	13	1	NO	-0,00331	0,003636	0,007364	0,009839	-0,005793	0,002177	0,005867	0,001161	0,005716
TNBC_525	42	GPL570	GSM308261	1		55	2	3	18	1	NO	-0,004347	0,003278	0,006517	0,008636	-0,003394	0,00446	0,004037	0,001883	0,006346
TNBC_526	42	GPL570	GSM308271	1		50	1	12	29	1	NO	-0,002267	0,001736	0,007052	0,009125	-0,004881	0,006547	0,006722	0,002546	0,006886
TNBC_527	42	GPL570	GSM308272	1		56	2	3	14	1	NO	-0,006303	0,004093	0,007949	0,007375	-0,004165	0,00245	0,00539	-0,000693	0,007313
TNBC_528	42	GPL570	GSM308273	1		55	1	12	19	1	NO	-0,005555	0,003557	0,012033	0,008034	-0,003753	0,007076	0,005215	0,004854	0,008739
TNBC_529	42	GPL570	GSM308275	1		39	2	3	7	1	NO	-0,004887	0,002493	0,004489	0,009111	-0,00456	0,005965	0,004956	0,004006	0,007419
TNBC_530	42	GPL570	GSM308280	1		51	2	12	16	1	NO	-0,010601	0,00366	0,007425	0,00784	-0,005407	0,00334	0,004874	0,001444	0,006651
TNBC_531	42	GPL570	GSM308281	1		66	1	3	5	1	NO	-0,00594	0,004217	0,010073	0,008815	-0,003992	0,004024	0,006648	0,000351	0,005825
TNBC_532	42	GPL570	GSM308283	1		55	1	3	13	1	NO	-0,004455	0,002911	0,008189	0,008955	-0,004467	0,0077	0,003809	0,003834	0,007712
TNBC_533	42	GPL570	GSM308285	1		50	2	3	7	1	NO	-0,010873	0,005043	0,010354	0,010524	-0,000495	0,002407	0,006023	0,001183	0,010368
TNBC_534	42	GPL570	GSM308290	1		62	2	3	6	1	NO	-0,00535	0,003759	0,005831	0,008835	-0,005853	0,002889	0,003386	0,001042	0,006989
TNBC_535	42	GPL570	GSM308295	1		63	2	3	11	1	NO	-0,00628	0,003328	0,007286	0,009447	0,000401	0,001135	0,004687	0,003445	0,00503
TNBC_536	42	GPL570	GSM308301	1		42	1	12	15	1	YES	-0,001226	0,003245	0,007116	0,009342	0,000165	0,00229	0,004356	0,001859	0,006547
TNBC_537	42	GPL570	GSM308307	1		36	2	3	29	1	NO	-0,003107	0,003375	0,008129	0,008535	-0,001539	0,004781	0,004435	0,004798	0,007968
TNBC_538	42	GPL570	GSM308309	1		42	2	12	20	1	NO	-0,002421	0,002759	0,007977	0,008697	0,001573	0,00545	0,005485	0,001266	0,006843
TNBC_539	42	GPL570	GSM308311	1		57	2	12	36	1	NO	-0,001688	0,004815	0,006773	0,011571	0,001777	0,006282	0,008142	0,004202	0,007889
TNBC_540	42	GPL570	GSM308312	1		44	2	12	115	1	NO	-0,001834	0,004107	0,00998	0,009668	0,000084	0,007801	0,00088	0,004085	0,008911
TNBC_541	42	GPL570	GSM308313	1					4	1		-0,003119	0,004435	0,009216	0,010017	-0,000645	0,005744	0,006647	0,002786	0,006789
TNBC_542	42	GPL570	GSM308314	1		58	2	3	8	1	NO	-0,002097	0,004611	0,006456	0,010579	-0,009505	0,006842	0,004544	0,003098	0,005246
TNBC_543	42	GPL570	GSM308316	1		43	1	3	0	1	NO	-0,001955	0,000909	0,003968	0,007428	-0,000175	0,00076	0,001138	0,000442	0,008684
TNBC_544	42	GPL570	GSM308324	1		57	2	3	14	1	NO	0,000754	0,005145	0,007278	0,009401	-0,000232	0,002858	0,005422	0,00054	0,009312
TNBC_545	42	GPL570	GSM308328	1		52	2	3	16	1	NO	-0,000353	0,005851	0,007959	0,009008	0,002148	0,002842	0,000136	0,001824	0,010708
TNBC_546	42	GPL570	GSM308329	1		40	2	3	1	1		0,000284	0,002066	0,006304	0,009432	0,000539	0,005789	-0,004751	0,001984	0,005682
TNBC_547	42	GPL570	GSM308330	1		31	2	3	0	1		-0,000544	0,002854	0,009229	0,008479	0,004824	0,00529	0,000513	0,001499	0,006377
TNBC_548	42	GPL570	GSM308333	1		30	1	3	18	1	NO	-0,002064	0,004909	0,009268	0,009278	-0,002882	0,006176	0,001622	0,004488	0,006912
TNBC_549	42	GPL570	GSM308335	1		38	2	12	50	1	NO	-0,00117	0,004916	0,008673	0,008408	-0,00058	0,006543	0,002144	0,002063	0,006448
TNBC_550	42	GPL570	GSM308336	1		28	2	12	8	1	NO	-0,003603	0,001947	0,007113	0,008092	-0,002966	0,004823	0,000136	0,000239	0,006598
TNBC_551	42	GPL570	GSM308338	1					10	1		-0,002623	0,004132	0,008455	0,008987	0,000456	0,005862	0,002678	0,002878	0,008145
TNBC_552	42	GPL570	GSM308339	1		49	2	3	3	1	NO	-0,003811	0,004461	0,01134	0,009147	-0,003794	0,00428	0,004943	0,004013	0,006803
TNBC_553	42	GPL570	GSM308340	1		46	2	12	60	1	NO	0,003317	0,004164	0,009772	0,0105	-0,00534	0,00217	0,005881	0,00057	0,007011
TNBC_554	42	GPL570	GSM308341	1		57	2	12	17	1	NO	-0,002263	0,004923	0,010327	0,011711	-0,004709	0,005455	0,004452	0,001608	0,005514
TNBC_555	42	GPL570	GSM308344	1		79	2	12	6	1	NO	0,000648	0,002217	0,008159	0,00897	-0,005327	0,001975	0,004821	-0,001172	0,007907
TNBC_556	42	GPL570	GSM308346	1		69	2	3	13	1	NO	0,000476	0,004259	0,006297	0,008416	-0,001377	0,001586	0,006739	0,00113	0,00517
TNBC_557	42	GPL570	GSM308348	1		39	2	3	25	1	YES	0,001916	0,003122	0,010187	0,008686	-0,003159	0,001368	0,004801	0,000733	0,00616
TNBC_558	42	GPL570	GSM308349	1		51	2	3	4	1	NO	0,001036	0,003916	0,007285	0,00889	-0,002584	0,000716	0,004424	0,000351	0,006355

TNBC_559	42	GPL570	GSM308350	1	65		3	15	1	NO	-0,000335	0,002474	0,007547	0,00909	-0,000338	0,006032	0,006071	0,001101	0,00475
TNBC_560	42	GPL570	GSM308352	1	44	2	3	0	1		-0,000997	0,003283	0,009932	0,008552	-0,001903	0,001621	0,003682	0,001569	0,006184
TNBC_561	42	GPL570	GSM308354	1	35	1	3	21	1	NO	0,001114	0,005176	0,007145	0,008887	-0,002717	0,004242	0,002521	0,002643	0,007462
TNBC_562	42	GPL570	GSM308356	1	39	2	3	18	1	YES	-0,001154	0,004555	0,009391	0,008012	-0,003945	0,002755	0,005793	0,000617	0,006918
TNBC_563	42	GPL570	GSM308364	1	44	1	12	29	1	NO	-0,001456	-0,000488	-0,000863	0,006767	-0,004723	-0,006312	0,005206	-0,005004	0,003512
TNBC_564	42	GPL570	GSM308374	1	51	2	3	6	1	NO	-0,000032	0,004122	0,009087	0,008325	-0,004453	0,002315	0,001574	0,003145	0,006635
TNBC_565	42	GPL570	GSM308376	1	56	2	3	6	1	NO	-0,001001	0,006223	0,011021	0,01023	0,001685	0,008861	0,003387	0,004449	0,007978
TNBC_566	42	GPL570	GSM308377	1	28	2	3	19	1	YES	0,000176	0,004159	0,00752	0,0098	-0,001513	0,006448	0,003893	0,002301	0,00734
TNBC_567	42	GPL570	GSM308378	1	41	2	3	15	1	NO	0,00014	0,004471	0,011297	0,008932	-0,000886	0,00353	0,006363	0,002917	0,006646
TNBC_568	42	GPL570	GSM308380	1	56	2	3	7	1	NO	-0,000956	0,00357	0,007984	0,008324	-0,003419	0,00209	0,005881	0,001523	0,006413
TNBC_569	42	GPL570	GSM308388	1	37	1	3	17	1	YES	-0,006146	0,002572	0,006537	0,007906	-0,003601	0,002194	0,003822	0,001194	0,008439
TNBC_570	42	GPL570	GSM308391	1				7	1		-0,002155	0,00396	0,008052	0,009415	0,001006	0,007005	0,002725	0,002439	0,005998
TNBC_571	42	GPL570	GSM308392	1				6	1		-0,006765	0,004951	0,007804	0,00826	-0,005008	0,002836	0,00287	0,000986	0,006019
TNBC_572	42	GPL570	GSM308393	1	40	2	3	33	1	NO	-0,001325	0,002772	0,010265	0,008129	-0,002231	0,005392	0,003354	0,001187	0,005927
TNBC_573	42	GPL570	GSM308394	1	34	2	3	22	1	YES	-0,002091	0,003522	0,006101	0,009426	-0,000479	0,007844	0,002962	0,002854	0,006832
TNBC_574	42	GPL570	GSM308404	1	50	2	3	8	1	NO	-0,002243	0,005815	0,010546	0,01072	-0,00112	0,004694	0,00196	0,003196	0,009181
TNBC_575	42	GPL570	GSM308405	1				24	1		0,000625	0,003861	0,007813	0,008404	-0,004274	0,001309	0,003911	-0,002127	0,007829
TNBC_576	42	GPL570	GSM308407	1	40	1	3	94	1	NO	0,000117	0,001742	0,008371	0,009054	-0,000089	0,004359	0,004414	0,0012	0,008448
TNBC_577	42	GPL570	GSM308408	1	45	2	12	4	1	NO	-0,004677	0,003609	0,008629	0,008288	-0,003084	0,005265	0,002818	0,002257	0,006913
TNBC_578	42	GPL570	GSM308438	1	63	2	12	35	1	NO	0,001685	0,001995	0,006656	0,008754	-0,002001	0,001488	0,003728	-0,001475	0,006057
TNBC_579	42	GPL570	GSM308449	1	56	2	3	47	1	NO	0,001285	0,002068	0,005704	0,008222	-0,001783	0,002755	0,008892	0,000743	0,005174

High levels of expression

Low levels of expression

Table S7. WikiPathways_2015.

Term	Overlap	P-value	Adjusted P-va	Old P	Old A	Odds Ratio	Combined Sc	Genes
RB in Cancer(I	26/87	3,12E-13	1,26E-10	0	0	5,56518761	160,252847	TOP2A;CDKN1A;PCNA;PRIM1;SMC2;CCNB2;CCNB1;CDC45;SKP2;RFC5;PLK4;BARD1;RRM1;RFC4;RRM2;H2AFZ;CDC25B;CCNA2;ANLN;CCNE1;CDK4;POLE2;RBP1;MCM3;CDK1;MCM4
Cell Cycle(Hor	25/104	1,69E-10	3,42E-08	0	0	4,47643604	100,712464	CDKN1A;PCNA;BUB1B;MCM10;CDC14A;CDC20;CCNB2;CCNB1;CDC45;SKP2;BUB1;CDKN2A;PLK1;CDC6;CDC25C;CDC25B;CCNA2;ESPL1;CCNE1;CDK4;CDK1;MCM3;MCM4;MCM2;MAD2L1
Gastric cance	13/43	2,33E-07	3,13E-05	0	0	5,6298991	85,9913803	TOP2A;UBE2C;AURKA;TPX2;ESM1;CENPF;HIST4H4;RUVBL1;MCM4;MYBL2;HIST1H4I;ECT2;KIF20E
DNA Replicati	12/41	1,00E-06	1,01E-04	0	0	5,45033383	75,2861341	RFC5;PRIM2;PCNA;CDC45;RFC4;PRIM1;POLE2;MCM3;MCM4;MCM10;CDC6;MCM2
Cori Cycle(Ho	6/15	7,78E-05	0,00224534	0	0	7,44878957	70,4749077	TPI1;PGAM1;SLC2A1;PGK1;GAPDH;PFKP
ATM Signaling	11/40	5,57E-06	3,22E-04	0	0	5,12104283	61,9517677	CDKN1A;CCNB1;RAD51;CRADD;FANCD2;CCNE1;H2AFX;CDK1;BRCA1;BID;CDC25C
DNA Replicati	11/41	7,25E-06	3,66E-04	0	0	4,99613935	59,1271582	RFC5;PCNA;CDC45;RFC4;PRIM1;POLE2;MCM3;MCM4;MCM10;CDC6;MCM2
Matrix Metall	8/29	1,05E-04	0,00283088	0	0	5,13709626	47,0585361	MMP12;MMP14;MMP7;MMP13;MMP16;BSG;TIMP1;MMP10
Gastric cance	9/35	7,14E-05	0,00221791	0	0	4,78850758	45,7190274	TOP2A;FANCI;RFC4;UBE2C;DSCC1;ATAD2;S100A6;COL9A1;COL9A3
G1 to S cell cy	13/60	1,40E-05	6,30E-04	0	0	4,03476102	45,0853765	PRIM2;CDKN1A;PCNA;CDKN2A;PRIM1;CDC45;CCNE1;CDK4;POLE2;MCM3;CDK1;MCM4;MCM2
Spinal Cord In	18/96	3,05E-06	2,06E-04	0	0	3,49162011	44,3395274	EGR1;NOS2;ARG1;CXCL3;CXCL2;KLK8;GFAP;MMP12;CXCL10;NR4A1;GJA1;VCAN;COL2A1;CDK4;CASP3;IL1B;SLIT1;CDK1
Matrix Metall	8/30	1,37E-04	0,00344977	0	0	4,96585971	44,1875771	MMP12;MMP14;MMP7;MMP13;MMP16;BSG;TIMP1;MMP10
DNA Damage	14/69	1,47E-05	5,95E-04	0	0	3,77837152	42,0356611	CDKN1A;H2AFX;BRCA1;CDC25C;CCNB2;CCNB1;RAD51;FANCD2;CCNE1;CDK4;CASP3;CDK1;PMAIP1;BID
Spinal Cord In	20/114	2,60E-06	2,10E-04	0	0	3,26701297	42,0199766	EGR1;NOS2;ARG1;MIF;KLK8;CXCL2;GFAP;MMP12;LGALS3;CXCL10;NR4A1;GJA1;VCAN;COL2A1;CDK4;CASP3;IL1B;CDK1;SLIT1;CCL2
miRNA regula	13/63	2,45E-05	8,99E-04	0	0	3,84262954	40,8003304	CDKN1A;H2AFX;BRCA1;CDC25C;CCNB2;CCNB1;RAD51;FANCD2;CCNE1;CDK4;CASP3;CDK1;BID
G1 to S cell cy	13/67	4,86E-05	0,00163621	0	0	3,61321882	35,8860627	CDKN1A;PCNA;CDKN2A;PRIM1;CCNB1;CDC45;CCNE1;CDK4;POLE2;MCM3;CDK1;MCM4;MCM2
Glycolysis and	10/47	1,61E-04	0,00381679	0	0	3,96212211	34,6152701	TPI1;PKM;PGAM1;SLC2A1;PGK1;ALDOC;ENO1;GAPDH;HK2;PFKP
Integrated Ca	8/35	4,33E-04	0,00873893	0	0	4,25645118	32,9689838	BARD1;BLM;CDK4;CASP3;PLK1;CDK1;BRCA1;CDC25B
Glycolysis and	10/49	2,31E-04	0,00518936	0	0	3,80040284	31,8176899	TPI1;PKM;PGAM1;SLC2A1;PGK1;ALDOC;ENO1;GAPDH;HK2;PFKP
IL1 and megal	6/24	0,00138024	0,0242442	0	0	4,65549348	30,6587456	IL1B;F2R;CCL2;TIMP1;PLA2G7;HBEGF
Urea cycle an	5/20	0,00348687	0,05031054	0	0	4,65549348	26,3442785	GATM;PYCRL;ACY1;ARG1;SMS
Leptin Insulin	4/15	0,00700997	0,08581903	0	0	4,96585971	24,6327587	SOCS2;SOCS3;SOCS1;PIK3R3
Retinol metak	7/37	0,00315297	0,04717777	0	0	3,52307615	20,2908414	SULT2B1;CYP26A1;CYP26B1;RDH10;ALDH1A2;RBP1;RARA
Senescence a	15/108	6,50E-04	0,01250637	0	0	2,58638527	18,9799544	CDKN1A;CEBPB;SERPINB2;PCNA;CDKN2A;IGFBP3;SERPINE1;IL24;FN1;INHBA;CDC25B;MMP14;IL1B;CCL3;CD44
Integrated Pa	23/195	3,34E-04	0,00710303	0	0	2,19643795	17,5807516	TOP2A;EGR1;CDKN1A;BLM;RRM1;PCNA;IGFBP3;PLK1;BUB1B;BRCA1;INHBA;PTGS2;CDC25C;CCNA2;GPRC5A;RAD51;WT1;CCNE1;CDK4;CASP3;RARA;BID;JUNE
Oncostatin M	10/65	0,00231757	0,03601144	0	0	2,86491907	17,3821426	SOCS3;EGR1;CEBPB;MMP13;CASP3;SERPINE1;OSM;CCL2;JUNB;CYR61
Vitamin A and	7/40	0,00496547	0,06917419	0	0	3,25884544	17,2889782	SULT2B1;CYP26A1;CYP26B1;RDH10;ALDH1A2;RBP1;RARA
miRNA Regul	14/105	0,00145391	0,02447409	0	0	2,48292986	16,2222259	CDKN1A;H2AFX;BRCA1;CDC25C;CCNB2;CCNB1;RAD51;FANCD2;CCNE1;CDK4;CASP3;CDK1;PMAIP1;BID
Complement	8/51	0,00548236	0,07382917	0	0	2,92109395	15,2078545	PROS1;SERPINE1;F2R;C3AR1;PLAUR;A2M;F3;KNG1
Type II interfe	7/44	0,00847812	0,10073999	0	0	2,96258676	14,1323294	SOCS3;CXCL10;SOCS1;NOS2;HIST4H4;IL1B;HIST1H4I
Cardiac Proge	8/53	0,00694574	0,08768991	0	0	2,81086399	13,9689469	SOX2;TBX20;CXCR4;PAX6;GATA4;NCAM1;SCN5A;INHBA
Kennedy path	3/14	0,03604482	0,29124218	0	0	3,99042299	13,2601436	PCYT1B;ETNK2;ETNK1
TP53 Networl	4/21	0,02380625	0,21858466	0	0	3,54704265	13,2581614	CDKN1A;CDKN2A;PMAIP1;BID
Purine metab	18/158	0,00209782	0,03390074	0	0	2,1214907	13,0829309	GUCY2C;PRIM2;RRM1;RRM2;PRIM1;AK4;NME5;ADCY8;AK7;APRT;NT5E;ATIC;PKM;POLE2;POLR2D;ENPP1;POLR2F;PDE9A
Alpha6-Beta4	9/64	0,00691587	0,09012939	0	0	2,61871508	13,0253229	DSP;LAMA5;CDKN1A;RTKN;MMP7;CASP3;PIK3R3;MET;EIF4E
Complement	8/55	0,00868617	0,10026318	0	0	2,70865075	12,8553203	PROS1;SERPINE1;F2R;C3AR1;PLAUR;A2M;F3;KNG1
PodNet: prote	30/306	0,0011106	0,02039471	0	0	1,82568372	12,4198563	COL18A1;LAMA5;CDKN1A;KHDRBS3;SH3KBP1;ARPC1B;SEMA3F;PTGS2;CXXC5;CYR61;CTNNA1;NCK2;KIRREL3;EGLN3;CDKN2A;IGFBP3;PLAUR;KRT7;F3;SMAD7;CYP26A1;CXCL10;VANGL2;P4HA1;WT1;SNAI1;RARA;BIRC5;EZR;ME
Prostaglandin	5/30	0,02058146	0,20280266	0	0	3,10366232	12,0526528	ANXA2;ANXA3;S100A6;PTGS2;S100A10
Prostaglandin	5/31	0,02348575	0,22065681	0	0	3,00354418	11,2673798	ANXA2;ANXA3;S100A6;PTGS2;S100A10
Type II interfe	7/50	0,01675586	0,18295592	0	0	2,60707635	10,6603534	SOCS3;CXCL10;SOCS1;NOS2;HIST4H4;IL1B;HIST1H4I
Monoamine T	5/32	0,0266418	0,23398451	0	0	2,90968343	10,5483993	IL1B;TNFRSF11B;CDC25C;SLC6A2;STX1A
Apoptosis-rel	7/52	0,02047331	0,21208249	0	0	2,50680418	9,74804203	SOCS3;CDKN1A;F2R;CTNNA1;BIRC5;IER3;SMAD7
TGF Beta Sign	7/52	0,02047331	0,20678043	0	0	2,50680418	9,74804203	TGIF1;BAMBI;SERPINE1;LEF1;INHBA;SKIL;SMAD7
Fluoropyrimid	5/35	0,03767616	0,29845431	0	0	2,66028199	8,72234013	RRM1;RRM2;TK1;UPP1;UMPS
TGF Beta Sign	7/55	0,02706861	0,23267487	0	0	2,37006941	8,55448231	TGIF1;BAMBI;SERPINE1;LEF1;INHBA;SKIL;SMAD7
Adipogenesis(	14/134	0,01298877	0,14576288	0	0	1,94557937	8,45095476	EGR2;CDKN1A;CEBPB;SERPINE1;OSM;GATA4;AHR;MIF;SOCS3;CYP26A1;CYP26B1;SOCS1;RARA;TRIB3
AGE/RAGE pa	8/66	0,02452038	0,22013851	0	0	2,25720896	8,37029682	LGALS3;MSR1;MMP14;MMP7;MMP13;NOS2;CASP3;EZR
Wnt Signaling	11/100	0,01831332	0,1946995	0	0	2,04841713	8,19392797	SOX2;FOSL1;MMP7;RACGAP1;WNT7B;TCF7;LEF1;FZD9;WNT7A;CD44;NKD2
Nucleotide M	3/19	0,07879036	0,50525881	0	0	2,94031167	7,47122796	RRM1;RRM2;MTHFD2
One Carbon M	4/28	0,06071227	0,45421773	0	0	2,66028199	7,45307114	AHCY;ATIC;MTHFD1L;MTHFD2
Serotonin Tra	2/11	0,11491923	0,64482456	0	0	3,38581344	7,32529461	IL1B;STX1A
Endochondral	7/59	0,03791907	0,294602	0	0	2,20938674	7,22977879	ADAMTS4;MMP13;COL2A1;MGP;ENPP1;SLC38A2;SOX5
Adipogenesis	13/129	0,02137056	0,20556445	0	0	1,87663303	7,21704449	EGR2;CEBPB;SERPINE1;OSM;GATA4;AHR;MIF;SOCS3;CYP26A1;CYP26B1;SOCS1;RARA;TRIB3
Wnt Signaling	10/93	0,02740598	0,23066703	0	0	2,00236279	7,20248674	SOX2;FOSL1;MMP7;RACGAP1;WNT7B;LEF1;FZD9;WNT7A;CD44;NKD2
Hypertrophy I	3/20	0,08914654	0,56273751	0	0	2,79329609	6,75272007	EIF4E;CYR61;HBEGF
Nucleotide M	3/20	0,08914654	0,55408001	0	0	2,79329609	6,75272007	RRM1;RRM2;MTHFD2
Nuclear recep	4/30	0,07478599	0,5120939	0	0	2,48292986	6,43854678	CYP26A1;NR1I2;RARA;ABCG1
One Carbon M	4/30	0,07478599	0,503559	0	0	2,48292986	6,43854678	AHCY;ATIC;MTHFD1L;MTHFD2
Hypertrophy I	3/21	0,10003659	0,5857215	0	0	2,66028199	6,12455237	EIF4E;CYR61;HBEGF
Sphingolipid I	3/21	0,10003659	0,57735405	0	0	2,66028199	6,12455237	CERS3;SPHK1;SGMS2

Integrated Br	14/151	0,03278089	0,27027507	0	0	1,72654063	5,90115986	BARD1;BLM;DCAKD;PLK1;AHR;BRCA1;HMGCR;CDC25B;SMAD7;AURKA;RAD51;CDK4;CASP3;BID
IL-4 Signaling	6/53	0,06356837	0,46693856	0	0	2,10814799	5,80929543	SOCS3;CEBPB;SOCS1;NFIL3;BIRC5;SOCS5
Oxidative Dan	4/32	0,0903495	0,55304844	0	0	2,32774674	5,59606568	CDKN1A;PCNA;CASP3;C3AR1
Trans-sulfurat	4/32	0,0903495	0,54479399	0	0	2,32774674	5,59606568	AHCY;MTHFD1L;MTHFD2;PSAT1
ErbB Signaling	6/54	0,06842329	0,47660361	0	0	2,06910821	5,54943517	CDKN1A;BCL2L11;TGFA;NRG2;EREG;HBEGF
Histone modi	1/5	0,24119161	0,95530795	0	0	3,72439479	5,29669869	HAT1
Endochondral	7/67	0,06730077	0,47700894	0	0	1,94557937	5,25030868	ADAMTS4;MMP13;COL2A1;MGP;ENPP1;SLC38A2;SOX5
Apoptosis Mo	8/80	0,06491417	0,46830939	0	0	1,86219739	5,09253129	BCL2L11;CRADD;CDKN2A;CASP3;BIRC5;PMAIP1;TNFRSF11B;BID
Cytokines and	3/23	0,12328628	0,65536392	0	0	2,42895312	5,08439672	CSF3;IL1B;CXCL2
Pathogenic Es	6/56	0,07875909	0,51320438	0	0	1,99521149	5,07055389	TUBB6;TUBB3;ARPC1B;NCK2;ARHGEF2;EZR
EGFR1 Signali	15/172	0,04415672	0,33659085	0	0	1,62400935	5,06692567	USP6NL;TGIF1;ERRFI1;CEBPB;SH3KBP1;KRT7;PIK3R3;EPS8;SOCS3;GJA1;SOCS1;ELF3;HAT1;NCK2;SPRY2
ErbB signaling	5/45	0,09211386	0,5472647	0	0	2,06910821	4,93426415	CDKN1A;TGFA;NRG2;EREG;HBEGF
Signaling Path	8/83	0,0770227	0,51011756	0	0	1,79488905	4,60147646	ERRFI1;CDKN1A;CCNE1;CDKN2A;CDK4;SPRY2;BRCA1;MET
Nuclear Rece	4/35	0,1163105	0,63499247	0	0	2,12822559	4,57886018	CYP26A1;NR1I2;RARA;ABCG1
Cytokines and	3/25	0,14826802	0,7582314	0	0	2,23463687	4,26532667	CSF3;IL1B;CXCL2
Cholesterol Bi	2/15	0,19107748	0,89761977	0	0	2,48292986	4,10943836	HMGCR;DHCR7
Cholesterol Bi	2/15	0,19107748	0,8873023	0	0	2,48292986	4,10943836	HMGCR;DHCR7
Nuclear Rece	4/37	0,13521729	0,70945178	0	0	2,01318637	4,02812866	NR4A2;NR4A1;NR1I2;RARA
Arrhythmoge	7/74	0,1017732	0,57910383	0	0	1,76153807	4,02512948	DSP;GJA1;LEF1;TCF7;CTNNA1;DSC2;ITGA9
Regulation of	1/6	0,28194975	1	0	0	3,10366232	3,92931845	GATA4
Nuclear Rece	4/38	0,14510881	0,75158924	0	0	1,96020778	3,78373298	NR4A2;NR4A1;NR1I2;RARA
Proteasome D	6/63	0,12133294	0,65358011	0	0	1,77352133	3,74074122	HIST1H2AH;H2AFZ;HIST1H2AK;H2AFX;HIST1H2AE;HIST1H2AC
Apoptosis Mo	2/16	0,21105567	0,897542	0	0	2,32774674	3,62112046	CASP3;BID
Neurotransmi	2/16	0,21105567	0,88819261	0	0	2,32774674	3,62112046	SLC38A2;STX1A
Wnt Signaling	9/106	0,11617019	0,64291449	0	0	1,58111099	3,40365603	VANGL2;RUVBL1;LEF1;FZD9;CDK1;FHL2;WNT7A;CDC25C;MAPK8IP1
Extracellular	3/28	0,18838487	0,89538219	0	0	1,99521149	3,3305432	TGFA;PROM1;MET
AMPK Signalir	6/69	0,16512348	0,83387356	0	0	1,61930208	2,91646303	CCNA2;CDKN1A;CCNB1;PIK3R3;HMGCR;CAMKK2
Eicosanoid Sy	2/18	0,2515005	0,95854906	0	0	2,06910821	2,85601141	PTGES2;PTGS2
Aryl Hydrocar	4/43	0,1983363	0,90031307	0	0	1,73227665	2,80246196	CDKN1A;PSRC1;NCOA7;AHR
Hair Follicle D	4/43	0,1983363	0,89030959	0	0	1,73227665	2,80246196	VCAN;LEF1;NCAM1;INHBA
IL-6 signaling	4/43	0,1983363	0,88052597	0	0	1,73227665	2,80246196	SOCS3;TIMP1;A2M;JUNB
Folate Metab	6/70	0,17300322	0,85235734	0	0	1,59616919	2,80039115	GPX2;AHCY;MTHFD2;IL1B;SERPINE1;CCL2
Primary Focal	6/70	0,17300322	0,84208798	0	0	1,59616919	2,80039115	KIRREL3;LAMA5;CDKN1A;PCNA;WT1;PLAUR
One carbon m	4/44	0,2096243	0,91062601	0	0	1,69290672	2,64506244	PCYT1B;ETNK2;GAD1;ETNK1
Regulation of	4/44	0,2096243	0,9009385	0	0	1,69290672	2,64506244	SPRED1;CDK1;KIF2C;AURKB
Ovarian Infert	3/31	0,23086659	0,95173573	0	0	1,80212651	2,64176478	EGR1;CEBPB;CDK4
Apoptosis(Ho	7/86	0,17880568	0,85997016	0	0	1,51574206	2,60928277	BCL2L11;CRADD;CDKN2A;CASP3;BIRC5;PMAIP1;BID
Blood Clotting	2/19	0,27181934	1	0	0	1,96020778	2,55340122	SERPINB2;SERPINE1
Ovarian Infert	3/32	0,24540284	0,96255095	0	0	1,74581006	2,45260854	EGR1;CEBPB;CDK4
ACE Inhibitor	1/8	0,35702133	1	0	0	2,32774674	2,39748545	KNG1
Folate-Alcoho	1/8	0,35702133	1	0	0	2,32774674	2,39748545	CEBPB
Androgen rec	7/90	0,20879954	0,91690233	0	0	1,44837575	2,26870772	PSMC3IP;CDKN1A;CCNE1;SNORD96A;FHL2;BRCA1;ETV5
Interferon typ	5/61	0,22904597	0,95396464	0	0	1,52639131	2,24964522	SOCS3;SOCS1;PRMT1;SNORD96A;EIF4E
miR-targeted	24/362	0,16859139	0,84087555	0	0	1,23460601	2,19794109	GALNT7;SLC38A1;CEBPB;SERPINE2;ANXA2;MYO10;CDKN2A;PLK1;CYR61;AURKB;CORO1C;MIR24-2;GJA1;NT5E;PKM;MTHFD2;P4HA2;PSAT1;RDH10;MIR140;TRIP13;SLC38A2;MET;SLC25A2
Semaphorin ir	5/62	0,23907963	0,95631854	0	0	1,50177209	2,14897367	SEMA5A;SEMA7A;CD72;TREM2;MET
Apoptosis Mo	2/21	0,31233523	1	0	0	1,77352133	2,06380812	CASP3;BID
Eicosanoid Sy	2/21	0,31233523	1	0	0	1,77352133	2,06380812	PTGES2;PTGS2
Globo Sphing	2/21	0,31233523	1	0	0	1,77352133	2,06380812	B3GALT5;ST6GALNAC4
PluriNetWork	19/284	0,19148787	0,87910341	0	0	1,24583628	2,05928117	CDKN1A;TFAP2C;KDM3A;CDKN2A;IGFBP3;LEF1;WWP2;BRCA1;UTF1;ETV5;SMAD7;SOX2;SOCS2;P4HA1;CASP3;MYBL2;SGK1;RCOR2;CD44
DNA Damage	7/93	0,23243861	0,94853735	0	0	1,40165395	2,04519413	FOSL1;CDKN1A;BCL2L11;CDKN2A;WNT7B;PMAIP1;WNT7A
Apoptosis(Mu	6/79	0,24998662	0,96185329	0	0	1,41432713	1,96074941	BCL2L11;CRADD;CDKN2A;CASP3;BIRC5;BID
Effects of Nitr	1/9	0,39156304	1	0	0	2,06910821	1,94001395	NOS2
Macrophage	1/9	0,39156304	1	0	0	2,06910821	1,94001395	F3
Mismatch rep	1/9	0,39156304	1	0	0	2,06910821	1,94001395	PCNA
Mismatch rep	1/9	0,39156304	1	0	0	2,06910821	1,94001395	PCNA
BDNF signalin	10/142	0,23297933	0,9412365	0	0	1,31140662	1,91046442	SHC4;GABRB3;EGR1;EGR2;BCL2L11;CASP3;SNORD96A;NCK2;NCAM1;EIF4E
Hedgehog Sig	2/22	0,33242598	1	0	0	1,69290672	1,86446262	CCNB1;CDK1
Signal Transd	2/22	0,33242598	1	0	0	1,69290672	1,86446262	RACGAP1;SPHK1
IL-1 Signaling	3/36	0,30464208	1	0	0	1,55183116	1,84453399	IL1RN;IL1B;IL1RAP
Peptide GPCR	5/66	0,28028459	1	0	0	1,4107556	1,7944103	CCR1;CXCR2;C3AR1;CXCR4;GCAT
SIDS Suscepti	11/161	0,24795514	0,96321035	0	0	1,27230878	1,77424406	SOX2;EGR1;IL1RN;GJA1;CEBPB;KCNJ8;CASP3;IL1B;SCN5A;FOXM1;GAPDH
Amyotrophic	3/37	0,31959132	1	0	0	1,50988978	1,72234971	CASP3;SLC1A2;BID

Chemokine si	11/165	0,27326676	1	0	0	1,24146493	1,61056092	SHC4;CCR1;CXCL10;CXCR2;ELMO1;PIK3R3;CXCR4;CCL2;CXCL3;ADCY8;CXCL2
Osteoblast(M	1/10	0,42425076	1	0	0	1,86219739	1,596705	TNFRSF11B
SREBF and mi	1/10	0,42425076	1	0	0	1,86219739	1,596705	HMGCR
Trans-sulfurat	1/10	0,42425076	1	0	0	1,86219739	1,596705	AHCY
Vitamin D Me	1/10	0,42425076	1	0	0	1,86219739	1,596705	DHCR7
Neural Crest I	7/101	0,29920591	1	0	0	1,29063186	1,55730646	CDH6;COL2A1;MIA;SNAI1;HOXA1;SOX5;GFAP
Blood Clotting	2/24	0,37205005	1	0	0	1,55183116	1,5343372	SERPINB2;SERPINE1
Signal Transdu	2/24	0,37205005	1	0	0	1,55183116	1,5343372	RACGAP1;SPHK1
NOD pathway	3/39	0,3494865	1	0	0	1,43245953	1,50593088	NLRP10;IL1B;CARD9
Oxidation by (	3/39	0,3494865	1	0	0	1,43245953	1,50593088	CYB5R2;CYP26A1;CYP26B1
Focal Adhesio	12/186	0,29805626	1	0	0	1,20141767	1,45428369	COMP;LAMA5;COL2A1;TNN;COL11A1;STYK1;TNC;FN1;MET;PGF;THBS4;ITGA9
Insulin Signali	10/153	0,30728311	1	0	0	1,21712248	1,43618721	SOCS3;EGR1;RPS6KA6;SOCS1;SLC2A1;ENPP1;PIK3R3;TRIB3;SGK1;EIF4E
Parkinsons Di	3/40	0,36438867	1	0	0	1,39664805	1,40996397	GP1BB;CCNE1;CASP3
Myometrial R	10/155	0,32139969	1	0	0	1,20141767	1,36369291	CNN2;GJA1;RGS1;CALD1;IGFBP3;IL1B;MAFF;RGS20;ADM;ADCY8
Ganglio Sphin	1/11	0,4551839	1	0	0	1,69290672	1,3324086	ST3GAL5
Corticotropin-	6/90	0,35380933	1	0	0	1,24146493	1,28987851	NR4A2;FOSL1;NR4A1;GJA1;CASP3;JUNB
Wnt Signaling	4/58	0,37910624	1	0	0	1,28427406	1,24566725	FOSL1;WNT7B;FZD9;WNT7A
EGF/EGFR Sig	10/161	0,36446578	1	0	0	1,15664434	1,16742728	USP6NL;EPS8;ERRFI1;GJA1;PCNA;SH3KBP1;NCK2;SPRY2;MYBL2;AURKA
Physiological	2/27	0,42957049	1	0	0	1,37940548	1,16555546	MYEF2;GATA4
IL-7 Signaling	3/43	0,40867706	1	0	0	1,29920748	1,16256986	CCNA2;BCL2L11;CDK4
Tryptophan m	3/43	0,40867706	1	0	0	1,29920748	1,16256986	PRMT1;ALDH1A2;DHCR24
Wnt Signaling	4/60	0,40375874	1	0	0	1,24146493	1,12593142	FOSL1;WNT7B;FZD9;WNT7A
Alanine and a	1/12	0,48445657	1	0	0	1,55183116	1,12465472	GAD1
Alanine and a	1/12	0,48445657	1	0	0	1,55183116	1,12465472	GAD1
Insulin Signali	10/163	0,37898869	1	0	0	1,14245239	1,10846318	SOCS3;EGR1;RPS6KA6;SOCS1;SLC2A1;ENPP1;PIK3R3;TRIB3;SGK1;EIF4E
Heart Develo	3/44	0,42324189	1	0	0	1,26968004	1,0916854	FOXC1;TBX20;GATA4
Metapathway	8/129	0,39004925	1	0	0	1,1548511	1,08727184	SULT2B1;CYP26A1;CYP26B1;MGST2;HS6ST2;CHST2;HS3ST1;HS2ST1
Leptin signali	4/61	0,41601928	1	0	0	1,22111305	1,07094504	SOCS2;SOCS3;IL1RN;IL1B
Focal Adhesio	11/183	0,39456658	1	0	0	1,11935362	1,04096238	LAMA5;COL2A1;TNN;COL11A1;STYK1;TNC;FN1;MET;PGF;THBS4;ITGA9
miR-targeted	10/166	0,40085764	1	0	0	1,12180566	1,02549744	MIR24-2;CEBPB;PKM;SERPINE2;ANXA2;MTHFD2;RDH10;PTRH1;KCNN4;CORO1C
IL-3 Signaling	6/97	0,42168711	1	0	0	1,15187468	0,9946342	SOCS2;SOCS3;BCL2L11;SLC2A1;RARA;BIRC5
Arylhydrocarb	2/29	0,46633039	1	0	0	1,28427406	0,97972247	CDKN1A;AHR
Dopminergic I	2/29	0,46633039	1	0	0	1,28427406	0,97972247	SOX2;NR4A2
Iron uptake a	7/115	0,42266618	1	0	0	1,13351146	0,97614899	GABRB3;CYBRD1;ATP6V0A4;ATP1A1;ATP1B1;SGK1;ATP6V1C2
Tryptophan m	3/46	0,45198148	1	0	0	1,21447656	0,96443292	PRMT1;ALDH1A2;DHCR24
Homologous	1/13	0,51215781	1	0	0	1,43245953	0,95849088	RAD51
Homologous	1/13	0,51215781	1	0	0	1,43245953	0,95849088	RAD51
Osteoclast(M	1/13	0,51215781	1	0	0	1,43245953	0,95849088	TNFRSF11B
Quercetin and	1/13	0,51215781	1	0	0	1,43245953	0,95849088	ACOX2
Serotonin and	1/13	0,51215781	1	0	0	1,43245953	0,95849088	ARC
Myometrial R	9/151	0,42303915	1	0	0	1,10991898	0,9548528	CNN2;GJA1;RGS1;IGFBP3;IL1B;MAFF;RGS20;ADM;ADCY8
Differentiatio	3/47	0,46612915	1	0	0	1,18863663	0,90727748	NT5E;WNT7B;INHBA
Heart Develo	3/47	0,46612915	1	0	0	1,18863663	0,90727748	FOXC1;TBX20;GATA4
Dopminergic I	2/30	0,48417559	1	0	0	1,24146493	0,90044401	SOX2;NR4A2
Inflammatory	2/30	0,48417559	1	0	0	1,24146493	0,90044401	LAMA5;FN1
Diurnally Regu	3/48	0,48011246	1	0	0	1,16387337	0,85397453	CEBPB;TUBB3;STBD1
Inflammatory	2/31	0,50164517	1	0	0	1,20141767	0,8288127	LAMA5;FN1
Biogenic Amir	1/14	0,53837191	1	0	0	1,330141	0,82363085	GAD1
Keap1-Nrf2(M	1/14	0,53837191	1	0	0	1,330141	0,82363085	CEBPB
Osteoblast Sig	1/14	0,53837191	1	0	0	1,330141	0,82363085	TNFRSF11B
Kit Receptor S	4/67	0,48803977	1	0	0	1,11175964	0,7975301	SPRED1;SOCS1;SH3KBP1;SOCS5
TSH signaling	4/67	0,48803977	1	0	0	1,11175964	0,7975301	EGR1;CCNE1;CDK4;TSHB
Diurnally Regu	3/50	0,50754333	1	0	0	1,11731844	0,75773542	CEBPB;TUBB3;STBD1
Id Signaling P	3/51	0,52097197	1	0	0	1,09541023	0,71427213	TGIF1;CCNA2;CCNE1
Biogenic Amir	1/15	0,56317864	1	0	0	1,24146493	0,71279752	GAD1
GPCRs, Class (	1/15	0,56317864	1	0	0	1,24146493	0,71279752	GPRC5A
TFs Regulate	1/15	0,56317864	1	0	0	1,24146493	0,71279752	MYEF2
Selenium Mic	5/88	0,51408798	1	0	0	1,0580667	0,70399617	GPX2;IL1B;SERPINE1;CCL2;PTGS2
Metapathway	10/181	0,50949511	1	0	0	1,02883834	0,69378173	SULT2B1;CYP26A1;CYP26B1;GPX2;MGST2;CHST5;HS6ST2;CHST2;HS3ST1;HS2ST1
Exercise-indu	3/52	0,53419836	1	0	0	1,07434465	0,67360125	CEBPB;STBD1;HIST1H2BL
TOR Signaling	2/34	0,55169991	1	0	0	1,09541023	0,65149636	PRR5;DDIT4
Deregulation	1/16	0,58665349	1	0	0	1,16387337	0,62071804	RAB27B

GPCRs, Class (	1/16	0,58665349	1	0	0	1,16387337	0,62071804	GPRC5A
ID signaling pa	1/16	0,58665349	1	0	0	1,16387337	0,62071804	CCNE1
Osteoclast Sig	1/16	0,58665349	1	0	0	1,16387337	0,62071804	TNFRSF11B
Transcriptiona	1/16	0,58665349	1	0	0	1,16387337	0,62071804	CEBPB
G13 Signaling	2/35	0,56757729	1	0	0	1,0641128	0,60269045	RTKN;CIT
Vitamin B12 N	3/54	0,56001635	1	0	0	1,03455411	0,5998234	IL1B;SERPINE1;CCL2
Peptide GPCR	4/73	0,55615841	1	0	0	1,02038213	0,59866035	CCR1;CXCR2;C3AR1;CXCR4
Amino Acid m	5/92	0,5539783	1	0	0	1,0120638	0,597755	PKM;ARG1;P4HA2;SMS;FARSB
Hematopoieti	3/55	0,57259598	1	0	0	1,01574403	0,56635339	CSF3;IL1B;CXCR4
FAS pathway a	2/36	0,58304405	1	0	0	1,03455411	0,55813422	CASP3;LMNB1
GPCRs, Other	5/94	0,57330999	1	0	0	0,99053053	0,55106057	CHRM3;PROKR2;F2R;CXCR2;CELSR1
ACE Inhibitor	1/17	0,6088679	1	0	0	1,09541023	0,54349211	KNG1
Drug Inductio	1/17	0,6088679	1	0	0	1,09541023	0,54349211	NR1I2
Mitochondria	1/17	0,6088679	1	0	0	1,09541023	0,54349211	MYEF2
Serotonin Rec	1/17	0,6088679	1	0	0	1,09541023	0,54349211	GATA4
Serotonin and	1/17	0,6088679	1	0	0	1,09541023	0,54349211	ARC
Statin Pathwa	1/17	0,6088679	1	0	0	1,09541023	0,54349211	HMGCR
Parkin-Ubiqui	4/76	0,58833635	1	0	0	0,98010389	0,51990246	TUBB6;TUBB3;GP1BB;CCNE1
IL-6 signaling	5/97	0,60146092	1	0	0	0,95989556	0,48800488	SOCS3;CEBPB;CASP3;SGK1;EIF4E
FAS pathway a	2/38	0,61274022	1	0	0	0,98010389	0,48006882	CASP3;LMNB1
SREBF and mi	1/18	0,6298895	1	0	0	1,03455411	0,47818216	HMGCR
Sulfation Biot	1/18	0,6298895	1	0	0	1,03455411	0,47818216	SULT2B1
TarBasePathw	1/18	0,6298895	1	0	0	1,03455411	0,47818216	GJA1
IL-1 signaling	3/58	0,60896129	1	0	0	0,96320555	0,47775051	IL1B;CCL2;IL1RAP
Alzheimers Di	4/79	0,61910659	1	0	0	0,94288476	0,45209233	CASP3;IL1B;BID;GAPDH
Kit receptor si	3/59	0,62061363	1	0	0	0,94688003	0,45170586	SOCS1;SNAI1;JUNB
MicroRNAs in	4/80	0,62903647	1	0	0	0,9310987	0,43162574	VMP1;MYEF2;PIK3R3;GATA4
Mitochondria	1/19	0,64978227	1	0	0	0,98010389	0,42254038	MYEF2
Serotonin Rec	1/19	0,64978227	1	0	0	0,98010389	0,42254038	EGR1
Interleukin-11	2/40	0,64079021	1	0	0	0,9310987	0,41438842	SOCS3;BIRC5
Oxidation by (	3/61	0,64319984	1	0	0	0,91583478	0,40415771	CYB5R2;CYP26A1;CYP26B1
Calcium Regul	7/143	0,65279262	1	0	0	0,91156516	0,38877869	CHRM3;GJA1;GJB4;RGS1;RGS20;ATP1B1;ADCY8
G13 Signaling	2/41	0,65420336	1	0	0	0,90838897	0,38546307	RTKN;CIT
Structural Pat	2/41	0,65420336	1	0	0	0,90838897	0,38546307	IL1RAP;EIF4E
TWEAK Signal	2/42	0,66721341	1	0	0	0,88676066	0,35882356	CASP3;CCL2
Regulation of	7/146	0,67403544	1	0	0	0,89283437	0,35219868	CXCL10;SOCS1;IL1B;PLK1;CCL3;PIK3R3;TREM1
Glycerophosp	1/21	0,68642033	1	0	0	0,88676066	0,3336571	AGPS
Triacylglyceric	1/21	0,68642033	1	0	0	0,88676066	0,3336571	AGPS
Calcium Regul	7/149	0,69444914	1	0	0	0,87485784	0,31900497	CHRM3;GJA1;GJB4;RGS1;RGS20;ATP1B1;ADCY8
Proteasome D	3/66	0,69543165	1	0	0	0,84645336	0,30745095	H2AFZ;H2AFX;HIST1H2AE
Glutathione n	1/22	0,70327719	1	0	0	0,84645336	0,29795512	GPX2
Oxidative Stre	1/22	0,70327719	1	0	0	0,84645336	0,29795512	JUNB
miR-targeted	8/172	0,71097297	1	0	0	0,86613832	0,29545786	GALNT7;SLC38A1;PKM;ANXA2;P4HA2;HOXA7;SLC38A2;MET
Non-odorant	12/256	0,72638446	1	0	0	0,87290503	0,27904666	CCR1;CHRM3;PROKR2;GPRC5A;CXCR2;F2R;C3AR1;FZD9;LPAR2;CXCR4;LPAR3;CELSR1
MAPK signalir	7/155	0,73272089	1	0	0	0,84099237	0,26154057	NR4A1;DUSP5;TMEM37;CASP3;IL1B;DUSP6;CDC25B
Histone Modi	3/70	0,73288889	1	0	0	0,7980846	0,24801371	HIST4H4;HIST1H3G;HIST1H4I
IL-9 Signaling	1/24	0,73432338	1	0	0	0,77591558	0,23960722	SOCS3
Triacylglyceric	1/24	0,73432338	1	0	0	0,77591558	0,23960722	AGPS
TNF-alpha NF-	8/179	0,75124111	1	0	0	0,83226699	0,23805218	UNC5CL;CRADD;PEG3;FANCD2;CASP3;PFDN2;MCC;TRAIP
miRNAs involv	3/71	0,74166445	1	0	0	0,78684397	0,2351549	CDKN1A;CCNE1;H2AFX
Alzheimers Di	3/73	0,75852556	1	0	0	0,7652866	0,21150898	CASP3;IL1B;BID
EPO Receptor	1/26	0,76212388	1	0	0	0,71622977	0,19456107	SOCS1
EPO Receptor	1/26	0,76212388	1	0	0	0,71622977	0,19456107	SOCS1
Glutathione a	1/26	0,76212388	1	0	0	0,71622977	0,19456107	AHCY
Synaptic Vesic	2/51	0,76710384	1	0	0	0,73027349	0,19361968	SLC38A1;STX1A
Wnt Signaling	2/51	0,76710384	1	0	0	0,73027349	0,19361968	GJA1;LEF1
FSH signaling	1/27	0,77491438	1	0	0	0,68970274	0,17587608	SGK1
GPCRs, Other	3/77	0,78956997	1	0	0	0,72553145	0,17141901	GPR39;FZD9;CELSR1
Nanoparticle-	1/28	0,78701774	1	0	0	0,6650705	0,15928737	FN1
RANKL/RANK	2/54	0,79407736	1	0	0	0,68970274	0,15902779	FHL2;TNFRSF11B
GPCRs, Class /	7/170	0,81336292	1	0	0	0,76678716	0,15840126	CCR1;CHRM3;F2R;CXCR2;C3AR1;CXCR4;GCAT
Cytoplasmic R	5/127	0,8184163	1	0	0	0,73314858	0,14691135	RPS6KA6;SNORD32A;RPL34;SNORD83B;RPL37

Toll-like recep	4/104	0,81617008	1	0	0	0,71622977	0,14548956	CXCL10;IL1B;CCL3;PIK3R3
MAPK Signalir	7/173	0,82698442	1	0	0	0,75349028	0,14314012	NR4A1;DUSP5;TMEM37;CASP3;IL1B;DUSP6;CDC25B
Delta-Notch S	3/81	0,81722371	1	0	0	0,68970274	0,13921126	NOV;LEF1;SKP2
Hypothetical I	1/30	0,80930858	1	0	0	0,62073246	0,13133147	ADCY8
IL17 signaling	1/30	0,80930858	1	0	0	0,62073246	0,13133147	CEBPB
TCA Cycle(Mu	1/30	0,80930858	1	0	0	0,62073246	0,13133147	PDK3
MicroRNAs in	4/109	0,84343345	1	0	0	0,68337519	0,11636122	MYEF2;MIR140;PIK3R3;GATA4
SIDS Susceptil	2/59	0,83292229	1	0	0	0,63125335	0,11540254	SCN5A;FOXM1
Hypothetical I	1/32	0,82926838	1	0	0	0,58193669	0,1089452	ADCY8
Monoamine C	1/32	0,82926838	1	0	0	0,58193669	0,1089452	CHRM3
Oxidative Stre	1/32	0,82926838	1	0	0	0,58193669	0,1089452	JUNB
Statin Pathwa	1/32	0,82926838	1	0	0	0,58193669	0,1089452	HMGCR
TNF alpha Sigi	3/86	0,84738942	1	0	0	0,64960374	0,10757108	CASP3;PLK1;BID
Alpha 6 Beta 4	1/33	0,83845127	1	0	0	0,56430224	0,09942939	LAMA5
Fatty Acid Bet	1/33	0,83845127	1	0	0	0,56430224	0,09942939	TPI1
Monoamine C	1/33	0,83845127	1	0	0	0,56430224	0,09942939	CHRM3
Toll Like Rece	1/33	0,83845127	1	0	0	0,56430224	0,09942939	CASP3
Fatty Acid Bet	1/34	0,84714069	1	0	0	0,54770512	0,09085798	TPI1
Signaling of H	1/34	0,84714069	1	0	0	0,54770512	0,09085798	MET
ESC Pluripoter	4/116	0,87581627	1	0	0	0,64213703	0,0851467	WNT7B;FZD9;WNT7A;SMAD7
Endothelin Pa	1/35	0,85536312	1	0	0	0,5320564	0,08312275	GUCY1B2
Regulation of	5/144	0,89149567	1	0	0	0,64659632	0,07426463	CHRM3;F2R;FN1;PIK3R3;EZR
PPAR signaling	2/68	0,88652282	1	0	0	0,54770512	0,06597021	ACOX2;OLR1
Regulation of	5/148	0,90445635	1	0	0	0,62912074	0,06317708	CHRM3;F2R;FN1;PIK3R3;EZR
IL-5 Signaling	1/40	0,89030255	1	0	0	0,46554935	0,05409401	SPRED1
miR-targeted	1/41	0,89620501	1	0	0	0,45419449	0,0497734	CEBPB
Cytoplasmic R	2/74	0,912882	1	0	0	0,50329659	0,04587481	RPL34;RPL37
Estrogen signa	2/74	0,912882	1	0	0	0,50329659	0,04587481	POLR2F;BRCA1
Eukaryotic Tra	1/42	0,90179015	1	0	0	0,44338033	0,04583375	POLR2F
IL-2 Signaling	1/42	0,90179015	1	0	0	0,44338033	0,04583375	SOCS3
Splicing factor	1/42	0,90179015	1	0	0	0,44338033	0,04583375	CHL1
mRNA Process	4/137	0,94061238	1	0	0	0,54370727	0,03328802	SNRPD1;PRMT1;SNRPG;HNRNPA1
Translation Fa	1/46	0,92128499	1	0	0	0,40482552	0,03318997	EIF4E
Allograft Reje	2/82	0,9391711	1	0	0	0,45419449	0,02850416	CASP3;IL1B
Selenium Met	1/48	0,92953042	1	0	0	0,38795779	0,0283503	GPX2
TCR Signaling	2/89	0,95581955	1	0	0	0,41847132	0,0189091	PSTPIP1;IL1B
Translation Fa	1/60	0,9637284	1	0	0	0,31036623	0,01146672	EIF4E
Notch Signalir	1/61	0,96568192	1	0	0	0,30527826	0,01066055	CDKN1A
SREBP signalli	1/64	0,97093404	1	0	0	0,29096834	0,00858262	HMGCR
IL-5 Signaling	1/68	0,97670919	1	0	0	0,27385256	0,0064537	SOCS1
GPCRs, Class A	7/259	0,98727531	1	0	0	0,50329659	0,00644539	CCR1;GPR39;CHRM3;CXCR2;F2R;C3AR1;CXCR4
mRNA process	11/398	0,99603849	1	0	0	0,51467767	0,00204295	BARD1;LSM8;NPM1;MYEF2;SNRPD1;PCBP4;PRMT1;RPL37;BRCA1;EIF4E;WDR55
G Protein Sigr	1/88	0,99230978	1	0	0	0,21161334	0,00163364	ADCY8
B Cell Recepto	1/93	0,99417128	1	0	0	0,20023628	0,00117054	ILF2
G Protein Sigr	1/96	0,99506432	1	0	0	0,1939789	9,60E-04	ADCY8
Integrin-medi	1/97	0,9953305	1	0	0	0,19197911	8,99E-04	ITGA9
Integrin-medi	1/100	0,99604598	1	0	0	0,18621974	7,38E-04	ITGA9
Odorant GPCF	1/122	0,99883208	1	0	0	0,15263913	1,78E-04	GPR142

Table S8. ***MMTV-R26*** ^{Met} **tumour versus controls.**

index	gene name	pvalue	log2FC
702	*Foxm1*	0.0236012658	1.41555447003448
459	*Aurka*	0.0030585754	1.81423953455477
544	*Aurkb*	0.0189702518	1.65732268945046
465	*Cdk1*	0.0119652018	1.81069099796158
2243	*Cdk2*	0.0068316319	0.657367566261536
1178	*Cdk4*	0.0057604442	1.0252517662403
4394	*Cdk6*	0.3377133336	0.350047272557211
7210	*Cdk5*	0.6642639085	-0.195680364197381
7604	*Cdk3-ps*	0.7824790071	-0.162854424239089
2630	*Wee1*	0.1340048910	0.584243867281207
2791	*Ccna1*	0.1175335340	-0.869644269247914
717	*Ccna2*	0.0385074205	1.39925211687813
581	*Ccnb1*	0.0302622659	1.57829056186044
634	*Ccnb2*	0.0222839844	1.4964121428095
1092	*Ccnd1*	0.0763555189	1.07948033011888
1454	*Ccnd2*	0.0009653828	-1.7032664178437
6145	*Ccnd3*	0.3000047990	-0.293298972372172
671	*Ccne1*	0.0145447076	1.44663741701661
3771	*Ccne2*	0.3245121465	0.420617509929978
7726	*Ccng1*	0.6626222740	-0.154629679856764
7486	*Ccng2*	0.8279623388	0.0840532742418599

Table S9A. A11_vs_No.

ID	logFC	AveExpr	t	P.Value	adj.P.Val	B
B7-H3	0,322884	-0,02672	2,05581	0,045767	0,999887	-3,97403
VHL-EPPK1	-0,3044	-0,01284	-2,76152	0,008363	0,999887	-3,31209
EMA	-0,24786	0,022802	-2,33677	0,02407	0,999887	-3,72549
HER3_pY12	-0,15254	0,046123	-2,23746	0,030372	0,999887	-3,81583

cut-off : P.Value<0.05

Table S9B. R547_vs No.

ID	logFC	AveExpr	t	P.Value	adj.P.Val	B
Rb_pS807_	-2,26559	-0,3406	-10,164	4,06E-13	8,75E-11	19,76045
c-Met_pY1	-1,63916	-0,11708	-3,31643	0,001835	0,032951	-1,91629
Mcl-1	-1,47577	-0,13578	-11,4027	1,01E-14	4,35E-12	23,37069
NDRG1_pT	-1,35527	0,115251	-2,61771	0,012093	0,115828	-3,65773
Connexin-4	-1,28807	-0,0405	-4,16411	0,000143	0,0051	0,504974
53BP1	-1,18178	0,044267	-3,111	0,00327	0,050334	-2,45599
Akt_pS473	-1,1062	0,043018	-2,08676	0,04274	0,241278	-4,77765
ARID1A	-1,05097	0,008024	-3,55905	0,000907	0,018618	-1,25293
Pdcd4	-1,02392	-0,38581	-3,47551	0,001159	0,02172	-1,48433
Akt2_pS47	-1,0155	-0,16412	-2,53926	0,014721	0,132182	-3,83529
Cdc6	-1,00313	0,051176	-6,1805	1,84E-07	2,64E-05	6,97937
HER2_pY12	-0,99549	-0,03211	-3,77354	0,000478	0,012771	-0,64537
Rad51	-0,96045	0,041184	-4,19764	0,000129	0,005091	0,606083
Shc_pY317	-0,94145	-0,16483	-3,02665	0,004123	0,057328	-2,67128
Hif-1-alpha	-0,8917	-0,06664	-5,43683	2,26E-06	0,000162	4,526962
Cyclin-D3	-0,77841	-0,15553	-3,7562	0,000504	0,012771	-0,69516
FN14	-0,75776	-0,03562	-5,84998	5,61E-07	4,84E-05	5,884974
EPHA2	0,750958	0,195028	3,173865	0,002745	0,045508	-2,2931
Src_pY416	-0,7413	-0,01775	-2,46144	0,017831	0,153707	-4,00744
HES1	-0,70096	-0,08295	-2,22051	0,031584	0,205169	-4,51425
p90RSK_pT	-0,65159	0,103213	-4,63795	3,16E-05	0,001945	1,962754
Pyk2_pY40	-0,64559	-0,01398	-2,65085	0,011119	0,108912	-3,58154
HER2	-0,63882	-0,10338	-3,96435	0,000267	0,008218	-0,08997
p38-MAPK	-0,62517	-0,1338	-3,06915	0,00367	0,052852	-2,56329
PHLPP	-0,61105	0,013041	-2,85531	0,006538	0,074151	-3,09655
Coup-TFII	-0,60824	-0,00264	-2,18078	0,034592	0,207074	-4,59387
GCN5L2	-0,59346	-0,23753	-2,12037	0,039652	0,227866	-4,7127
PLC-gamma	-0,58438	-0,08715	-2,49914	0,016257	0,142994	-3,92454
JNK_pT183	-0,57219	-0,13916	-2,02355	0,04912	0,251844	-4,89748
ULK1_pS75	-0,54843	-0,1893	-2,95175	0,005052	0,063966	-2,85921
Snail	-0,5303	0,250236	-2,04292	0,047082	0,247468	-4,86109
NF-kB-p65_	-0,52334	-0,21469	-2,44942	0,018362	0,155176	-4,03368
C-Raf_pS33	-0,52271	0,00189	-2,70278	0,009735	0,102341	-3,46078
Chk1	-0,51207	0,162593	-2,67078	0,010568	0,105925	-3,5354
Mnk1	-0,49885	-0,06276	-2,06787	0,044566	0,243139	-4,81378
Smad1	-0,49774	-0,19949	-2,28733	0,027045	0,194271	-4,37778
ERRalpha	-0,49309	-0,0124	-4,19553	0,00013	0,005091	0,599714
PDK1_pS24	-0,48156	-0,08182	-2,78229	0,007922	0,085362	-3,27266
Aurora-ABC	-0,47789	0,075737	-3,7764	0,000474	0,012771	-0,63713
Histone-H3	-0,47253	0,093741	-2,67731	0,010393	0,105925	-3,52021
MMP14	-0,45283	0,114206	-2,24761	0,029667	0,205169	-4,45927
RBM15	-0,41724	0,077333	-2,86792	0,006323	0,073649	-3,06581
IRS1	-0,39491	-0,16165	-2,02407	0,049064	0,251844	-4,89652
PI3K-p85	-0,39342	-0,11462	-2,08293	0,043105	0,241278	-4,78501
DNMT1	0,374448	0,187822	3,149138	0,002941	0,046953	-2,35741
IR-b	-0,3613	0,194304	-3,56096	0,000902	0,018618	-1,24759
RPA32_pS4	-0,33419	-0,05857	-3,61927	0,000759	0,017218	-1,08425
4E-BP1_pS	-0,33365	-0,09775	-2,80358	0,007493	0,082804	-3,22163
SOD1	0,33142	0,07373	4,141726	0,000154	0,0051	0,437667

MITF	-0,32289	-0,12981	-3,73257	0,000541	0,012952	-0,76284
PKC-b-II_pS	-0,31958	-0,02681	-2,59914	0,012673	0,118745	-3,70013
SHP2	-0,29991	-0,06484	-5,99037	3,49E-07	3,76E-05	6,349228
HER3_pY12	-0,29196	0,046123	-4,28241	9,88E-05	0,004939	0,863208
S6	0,2893	0,054635	2,355064	0,023047	0,182137	-4,2362
MSI2	0,286749	-0,04978	3,494525	0,001096	0,02148	-1,43193
Wee1_pS6	-0,24814	0,100067	-2,91346	0,0056	0,067042	-2,95408
Akt2	-0,24708	-0,17563	-2,0576	0,045587	0,24326	-4,83331
Atg4B	-0,23819	-0,10914	-3,22048	0,002409	0,041526	-2,17102
GSK-3a-b	-0,23404	0,00883	-2,35012	0,023319	0,182137	-4,24663
CD45	0,225285	0,008777	3,068295	0,003679	0,052852	-2,56547
Bcl2	0,217647	0,084614	2,125147	0,03923	0,227866	-4,70341
CD49b	0,179144	0,083165	2,941456	0,005194	0,063966	-2,8848
Bcl-xL	-0,17639	-0,0211	-2,07496	0,043873	0,242425	-4,80025
HSP27	0,169276	0,015585	2,993791	0,004509	0,060053	-2,75411
Annexin-VI	0,165943	0,064325	2,433534	0,019085	0,158184	-4,0682
Notch1-cle	0,165721	0,058174	2,343927	0,023665	0,182137	-4,25969
PAI-1	0,165641	0,03994	4,268752	0,000103	0,004939	0,821628
SF2	0,161461	0,101404	2,29015	0,026866	0,194271	-4,37194
Rheb	0,154196	0,01574	2,98661	0,004598	0,060053	-2,77213
ATP5A	0,153526	0,072204	2,198071	0,033253	0,205169	-4,55936
14-3-3-eps	0,151653	0,027058	2,584417	0,013151	0,120599	-3,73357
CD29	0,150243	0,064657	2,311589	0,025546	0,189836	-4,32744
MEK2	0,145272	-0,0442	2,182739	0,034438	0,207074	-4,58997
Gli1	0,14334	-0,00908	2,197165	0,033322	0,205169	-4,56117
MelanA	0,141021	-0,03267	2,170769	0,035389	0,208942	-4,61375
Bid	0,139832	0,076547	2,233226	0,030671	0,205169	-4,48852
Tau	0,139304	0,086972	2,056308	0,045717	0,24326	-4,83577
Rb	0,135846	0,097094	2,40044	0,020674	0,168122	-4,13957
UQCRC2	0,134148	0,034731	2,201867	0,032965	0,205169	-4,55175
Collagen-V	0,13084	0,014401	2,231359	0,030803	0,205169	-4,49231
BiP-GRP78	0,127991	0,042868	2,24745	0,029678	0,205169	-4,45961
CD31	0,125889	0,029025	2,209031	0,032428	0,205169	-4,53737
Syk	0,123404	0,060814	2,331125	0,024395	0,184457	-4,2866
TIGAR	0,122938	-0,03684	2,208326	0,032481	0,205169	-4,53878
ADAR1	0,122292	0,069972	2,018462	0,049668	0,251844	-4,907

cut-off : P.Value<0.05

Table S9C. Adav_vs_No.

ID	logFC	AveExpr	t	P.Value	adj.P.Val	B
p53	0,893274	0,016506	2,733352	0,008997	0,271392	-3,02026
cdc2_pY15	-0,88263	0,010496	-9,99755	6,77E-13	2,92E-10	19,00459
Pyk2_pY40	-0,85188	-0,01398	-3,49786	0,001086	0,066855	-1,08732
Chk1	-0,8297	0,162593	-4,32739	8,56E-05	0,009228	1,290798
p38-MAPK	0,787398	-0,03056	2,894573	0,00589	0,271392	-2,63827
CDK1_pT1	0,780187	-0,00726	2,727352	0,009138	0,271392	-3,03418
Snail	-0,68821	0,250236	-2,65124	0,011108	0,281613	-3,20892
Rad51	-0,63621	0,041184	-2,78055	0,007958	0,271392	-2,91
JNK2	0,631696	-0,12274	2,102989	0,041224	0,668733	-4,35644
Hif-1-alpha	-0,61398	-0,06664	-3,74354	0,000523	0,045111	-0,40843
MMP14	-0,49777	0,114206	-2,47066	0,017434	0,385051	-3,60906
EMA	-0,46177	0,022802	-4,35352	7,88E-05	0,009228	1,369238
Mcl-1	-0,4581	-0,13578	-3,53955	0,000961	0,066855	-0,97383
RPA32	-0,36247	-0,0536	-2,85045	0,006622	0,271392	-2,74429
RBM15	0,353431	0,077333	2,429356	0,019279	0,395682	-3,69765
ERRalpha	-0,3129	-0,0124	-2,66236	0,010797	0,281613	-3,18361
VHL-EPPK1	-0,28053	-0,01284	-2,54495	0,014514	0,347533	-3,44696
PARP	-0,26728	-0,03808	-2,46061	0,017868	0,385051	-3,63073
SHP2	-0,24647	-0,06484	-4,92299	1,24E-05	0,002681	3,117465
DDB-1	-0,23711	0,062349	-2,09577	0,041893	0,668733	-4,37016
c-Abl_pY41	-0,22358	0,002527	-2,71453	0,009445	0,271392	-3,06385
SOD1	0,218735	0,07373	2,733509	0,008993	0,271392	-3,0199
HER3_pY1	-0,2131	0,046123	-3,12582	0,003138	0,169078	-2,06519
MITF	-0,20391	-0,12981	-2,35725	0,022927	0,449157	-3,84954
Raptor	-0,14889	-0,08896	-2,18487	0,034271	0,596674	-4,19821
C-Raf	-0,14113	0,036654	-2,04081	0,0473	0,728084	-4,47329
MIG6	0,125608	0,101079	2,180557	0,03461	0,596674	-4,20667
PAI-1	0,085934	0,03994	2,214627	0,032014	0,596674	-4,13952

cut-off : P.Value<0.05

Table S9D. A11+R547_vs_No.

ID	logFC	AveExpr	t	P.Value	adj.P.Val	B
Rb_pS807_	-2,29183	-0,3406	-10,2817	2,83E-13	1,22E-10	20,1024
c-Met_pY1	-2,10188	-0,11708	-4,25263	0,000109	0,001732	0,821176
Connexin-4	-1,72293	-0,0405	-5,56994	1,44E-06	7,77E-05	5,002576
NDRG1_pT	-1,6529	0,115251	-3,19258	0,002605	0,016512	-2,19027
Histone-H3	1,598981	0,365476	4,665457	2,89E-05	0,000595	2,095144
Akt_pS473	-1,52954	0,043018	-2,88535	0,006036	0,030131	-2,96795
Akt2_pS47	-1,435	-0,16412	-3,58822	0,000832	0,008152	-1,11931
HER2_pY12	-1,33878	-0,03211	-5,07481	7,54E-06	0,000271	3,395449
53BP1	-1,24197	0,044267	-3,26945	0,002097	0,013907	-1,98797
Stat3	-1,17506	-0,32293	-2,81043	0,007359	0,034105	-3,14962
SHP-2_pY5	-1,14238	-0,1488	-2,83113	0,006969	0,033108	-3,09976
Rad51	-1,14162	0,041184	-4,98944	1E-05	0,000308	3,121753
Mcl-1	-1,10592	-0,13578	-8,545	6,78E-11	1,46E-08	14,75228
Akt1_pS47	-1,09595	-0,06283	-2,87423	0,006217	0,030317	-2,99511
ARID1A	-1,08243	0,008024	-3,6656	0,000661	0,006627	-0,90172
Rictor	-1,06039	-0,12713	-3,07822	0,00358	0,020301	-2,48566
Stat5a	-1,05491	-0,3732	-2,13957	0,037978	0,09966	-4,61741
Shc_pY317	-1,05035	-0,16483	-3,37677	0,001543	0,01143	-1,70071
Hif-1-alpha	-1,04831	-0,06664	-6,39173	8,97E-08	9,67E-06	7,713684
Src_pY416	-1,00952	-0,01775	-3,35204	0,001657	0,011708	-1,76739
Pdcd4	-0,99856	-0,38581	-3,38943	0,001488	0,011384	-1,66646
Cdc6	-0,98756	0,051176	-6,08459	2,54E-07	2,19E-05	6,696983
NF-kB-p65_	-0,95184	-0,21469	-4,455	5,7E-05	0,000961	1,440272
FAK_pY397	-0,95056	-0,14486	-2,90697	0,005698	0,028891	-2,91493
Caspase-3-	0,94917	0,182884	3,392577	0,001475	0,011384	-1,65794
HES1	-0,93768	-0,08295	-2,9704	0,004804	0,025252	-2,75781
PTEN	-0,93694	-0,31196	-2,43659	0,018944	0,062306	-4,0047
Cyclin-D3	-0,92211	-0,15553	-4,44962	5,8E-05	0,000961	1,423672
Pyk2_pY40	-0,90613	-0,01398	-3,72063	0,000561	0,006041	-0,74549
PHLPP	-0,89112	0,013041	-4,164	0,000143	0,002132	0,553697
FOXO3	-0,86716	-0,14293	-2,51256	0,015728	0,055943	-3,83818
HER2	-0,86001	-0,10338	-5,33703	3,15E-06	0,000136	4,242825
H2AX_pS14	0,853326	0,173534	4,894752	1,37E-05	0,000368	2,819654
Coup-TFII	-0,84421	-0,00264	-3,02683	0,004121	0,022204	-2,61617
FRS2-a_pY1	-0,82822	-0,13486	-2,49132	0,016573	0,056243	-3,88512
p70-S6K1	-0,82207	-0,31847	-3,15838	0,002867	0,017401	-2,27932
IRS2	-0,81457	-0,1149	-2,65633	0,010965	0,041448	-3,51276
mTOR	-0,77909	-0,09916	-2,76846	0,008213	0,035399	-3,24995
JNK_pT183	-0,76043	-0,13916	-2,68926	0,010079	0,0406	-3,43638
PRAS40_pT	-0,75448	-0,16259	-2,88251	0,006082	0,030131	-2,9749
S6	0,736784	0,054635	5,99783	3,41E-07	2,45E-05	6,410296
DVL3	-0,73359	-0,25384	-2,66601	0,010697	0,041448	-3,4904
ULK1_pS75	-0,72225	-0,1893	-3,88731	0,000338	0,00394	-0,26522
WIPI1	-0,71426	-0,2543	-2,32003	0,025043	0,07253	-4,25255
GCN5L2	-0,6997	-0,23753	-2,49994	0,016225	0,055943	-3,8661
FN14	-0,69583	-0,03562	-5,37186	2,8E-06	0,000134	4,356063
Caspase-7-	0,690076	0,19787	5,007771	9,41E-06	0,000308	3,180425
p38-MAPK	-0,68958	-0,1338	-3,38535	0,001506	0,011384	-1,67749
EPHA2	0,66279	0,195028	2,801229	0,007539	0,034412	-3,17171

Myosin-IIa	-0,6588	-0,20646	-2,05618	0,04573	0,115938	-4,77794
Smad1	-0,65278	-0,19949	-2,99984	0,004436	0,023603	-2,68412
XIAP	-0,64464	-0,31104	-2,50512	0,016019	0,055943	-3,85467
AMPKa_pT	0,637662	0,086728	2,402575	0,020568	0,064501	-4,07799
PLC-gamma	-0,62274	-0,08715	-2,6632	0,010774	0,041448	-3,49688
PDK1	-0,61755	-0,14864	-2,74466	0,008737	0,036812	-3,30639
Akt2	-0,6143	-0,17563	-5,11559	6,58E-06	0,000258	3,52657
Akt_pT308	-0,60106	0,080566	-2,24855	0,029602	0,082848	-4,39988
DM-Histon	0,589655	0,167256	4,114998	0,000167	0,002229	0,406843
DUSP6	-0,58146	0,009088	-2,04799	0,046561	0,116331	-4,79343
Chk1_pS34	-0,57914	-0,24295	-2,40184	0,020604	0,064501	-4,07956
Rad23A	-0,57874	-0,07387	-3,16779	0,002792	0,017192	-2,25487
Jak2	-0,57759	-0,22193	-2,2394	0,030237	0,084077	-4,41848
RRM2	-0,57091	-0,05838	-2,45146	0,018271	0,060575	-3,9724
PDK1_pS24	-0,56093	-0,08182	-3,24084	0,002274	0,01463	-2,0636
ERRalpha	-0,55114	-0,0124	-4,68943	2,67E-05	0,000595	2,170383
mTOR_pS2	-0,53647	-0,13298	-2,05253	0,046099	0,116191	-4,78486
Mnk1	-0,52864	-0,06276	-2,19138	0,033766	0,091854	-4,51508
PTPN12	-0,51905	-0,19282	-2,0303	0,048401	0,117209	-4,82671
UBAC1	-0,51671	-0,16793	-2,14466	0,037544	0,099476	-4,60744
SOD1	0,514315	0,07373	6,427349	7,95E-08	9,67E-06	7,831688
4E-BP1_pT	-0,50097	-0,04807	-3,67529	0,000642	0,006591	-0,87431
TAZ	-0,4996	-0,1195	-3,11465	0,003237	0,019377	-2,3923
H2AX_pS13	0,486855	0,008345	2,326162	0,024683	0,072369	-4,23976
FoxO3a_pS	-0,48521	-0,12973	-2,13753	0,038153	0,09966	-4,62141
Rictor_pT1	-0,47208	-0,05303	-3,10129	0,003359	0,019563	-2,4266
Chk1	-0,45662	0,162593	-2,38155	0,021634	0,065204	-4,12291
eEF2K	-0,4502	-0,05665	-2,09476	0,041987	0,107079	-4,70433
PI3K-p85	-0,44397	-0,11462	-2,35055	0,023296	0,069245	-4,18857
PAK4	-0,43814	-0,11223	-3,57622	0,000862	0,008259	-1,1528
DNMT1	0,418827	0,187822	3,522371	0,001011	0,008853	-1,30247
IRS1	-0,41406	-0,16165	-2,12223	0,039487	0,10191	-4,65122
MMP14	-0,41074	0,114206	-2,03872	0,047517	0,117029	-4,8109
EMA	-0,40236	0,022802	-3,79339	0,00045	0,004975	-0,53714
KAP1	0,376733	0,102915	2,143759	0,037621	0,099476	-4,60921
Caspase-8	0,365651	0,089702	4,624539	3,3E-05	0,000646	1,96701
cdc2_pY15	0,365367	0,010496	4,138511	0,000155	0,002229	0,477218
Erk5	-0,34933	-0,12646	-3,95072	0,000278	0,003333	-0,07982
Lasu1	-0,34757	-0,10668	-3,51686	0,001027	0,008853	-1,31771
Bcl2	0,344742	0,084614	3,366136	0,001591	0,01143	-1,72941
TRAP1	0,34178	0,101822	2,046688	0,046694	0,116331	-4,79589
MSI2	0,336344	-0,04978	4,098924	0,000176	0,002229	0,358832
SF2	0,328865	0,101404	4,664607	2,9E-05	0,000595	2,092478
4E-BP1_pS	-0,31499	-0,09775	-2,64677	0,011235	0,041448	-3,53481
RPA32_pS4	-0,3135	-0,05857	-3,39528	0,001463	0,011384	-1,65062
Aurora-ABC	-0,30407	0,075737	-2,40284	0,020555	0,064501	-4,07742
Akt1	-0,29883	-0,05668	-2,50279	0,016111	0,055943	-3,85981
MITF	-0,29732	-0,12981	-3,43703	0,001296	0,010746	-1,53703
MLH1	0,294071	0,049452	2,812933	0,007311	0,034105	-3,14361
ATP5A	0,293366	0,072204	4,200206	0,000128	0,001971	0,662682
Cyclin-B1	0,291068	0,036954	2,663442	0,010768	0,041448	-3,49633

SHP2	-0,288	-0,06484	-5,7525	7,8E-07	4,8E-05	5,60165
CD49b	0,280556	0,083165	4,606587	3,5E-05	0,000655	1,910916
IRF-1	0,280384	0,061071	3,683751	0,000626	0,006583	-0,85033
Caspase-8-	0,27515	0,072456	2,747617	0,008671	0,036812	-3,29939
ADAR1	0,271711	0,069972	4,484658	5,18E-05	0,00093	1,531909
Annexin-VI	0,271659	0,064325	3,983839	0,000251	0,003096	0,017558
GRB7	0,270155	0,054688	2,794121	0,007681	0,034412	-3,18874
Annexin-I	0,269303	0,056642	2,359733	0,022792	0,068217	-4,16918
XPA	0,265541	-0,00526	2,654045	0,011029	0,041448	-3,51804
SCD	0,265092	0,107627	3,2823	0,002022	0,013618	-1,95385
Cdc42	0,264264	0,085081	2,962677	0,004906	0,025474	-2,77706
Tau	0,261725	0,086972	3,8634	0,000364	0,004127	-0,33475
Atg4B	-0,26122	-0,10914	-3,53186	0,000983	0,008853	-1,27617
Src	0,256932	0,012489	2,319502	0,025074	0,07253	-4,25366
Rheb	0,255832	0,01574	4,955205	1,12E-05	0,000322	3,012348
Syk	0,254503	0,060814	4,807616	1,82E-05	0,000461	2,543092
Porin	0,252939	0,069191	3,040147	0,003974	0,021681	-2,58248
NAPSIN-A	0,250254	-0,04255	3,311146	0,001863	0,012949	-1,87701
Collagen-V	0,240595	0,014401	4,103134	0,000174	0,002229	0,371399
HER3_pY12	-0,23531	0,046123	-3,45149	0,001243	0,010506	-1,49753
cdc25C	0,235098	0,079258	2,165246	0,035836	0,095933	-4,56694
Rb	0,23336	0,097094	4,123541	0,000163	0,002229	0,432391
AMPK-a2_	0,229382	-0,05915	2,646325	0,011247	0,041448	-3,53584
p21	0,226293	0,021595	2,271983	0,028032	0,079486	-4,35198
Notch1-cle	0,224098	0,058174	3,169607	0,002778	0,017192	-2,25014
14-3-3-bet	0,220424	0,061595	3,087975	0,003485	0,020026	-2,46073
CD29	0,21904	0,064657	3,370087	0,001573	0,01143	-1,71875
N-Ras	0,209379	0,051875	3,062862	0,003734	0,020633	-2,52481
D-a-Tubulin	0,203756	0,028271	3,395323	0,001463	0,011384	-1,6505
CD31	0,20223	0,029025	3,548618	0,000935	0,008765	-1,22968
Glutamate-	0,201878	-0,02475	2,184328	0,034314	0,092432	-4,52912
XBP-1	0,201576	0,02769	3,250042	0,002216	0,01447	-2,03931
BiP-GRP78	0,200515	0,042868	3,520935	0,001015	0,008853	-1,30644
DNA-Ligase	0,198414	0,085852	2,033771	0,048035	0,117209	-4,8202
Patched	0,1962	-0,01103	2,210008	0,032355	0,088823	-4,4778
GATA6	0,192752	0,04835	2,580979	0,013265	0,048451	-3,68498
Gli1	0,191127	-0,00908	2,92966	0,005362	0,027511	-2,85898
RIP3	0,187771	0,039092	2,340856	0,023838	0,070372	-4,20896
HSP27	0,185949	0,015585	3,288678	0,001986	0,013586	-1,9369
DM-K9-His	0,184723	-0,00753	2,030252	0,048406	0,117209	-4,8268
PAI-1	0,183096	0,03994	4,718609	2,43E-05	0,000582	2,262134
DAPK2	0,181077	0,015904	2,495172	0,016417	0,056155	-3,87664
Slfn11	0,180745	-0,01853	2,189827	0,033886	0,091854	-4,51817
MRAP	0,180454	0,053994	2,871651	0,00626	0,030317	-3,00141
Chk1_pS29	0,179698	0,044032	2,66379	0,010758	0,041448	-3,49552
PR	0,177004	0,048848	2,792653	0,00771	0,034412	-3,19225
YAP	0,174133	-0,02923	2,408514	0,020276	0,064501	-4,06525
FOXM1	0,174033	-0,00113	2,229208	0,030957	0,085528	-4,43912
PD-1	0,173112	0,011749	2,294632	0,026585	0,076389	-4,30532
TIGAR	0,173096	-0,03684	3,109319	0,003285	0,019396	-2,406
Smad4	0,172685	0,042306	3,070192	0,00366	0,020485	-2,50614

PCNA	0,171705	0,071675	2,124914	0,03925	0,101909	-4,64601
UQCRC2	0,170038	0,034731	2,79095	0,007745	0,034412	-3,19632
CD134	0,169887	0,019186	2,694285	0,00995	0,040458	-3,42468
Ambra1_pS	0,169089	0,121705	2,734925	0,008961	0,037135	-3,32936
Notch3	0,166996	0,090122	2,769574	0,008189	0,035399	-3,24731
PDH	0,165939	0,028879	2,483139	0,016909	0,056936	-3,90313
Cyclin-D1	0,163783	0,037598	2,776165	0,00805	0,035399	-3,23162
MIF	0,163741	-0,0104	2,668715	0,010624	0,041448	-3,48412
LAD1	0,162616	-0,0278	2,252256	0,029349	0,082676	-4,39233
MelanA	0,162572	-0,03267	2,502521	0,016122	0,055943	-3,86041
MEK2	0,161968	-0,0442	2,43359	0,019082	0,062306	-4,01119
N-Cadherir	0,160043	0,03129	2,381597	0,021631	0,065204	-4,1228
14-3-3-eps	0,1599	0,027058	2,724949	0,009195	0,037742	-3,35285
Bid	0,158638	0,076547	2,533567	0,014931	0,054076	-3,79147
PD-L1	0,158394	0,052266	2,829978	0,00699	0,033108	-3,10254
MIG6	0,157951	0,101079	2,742023	0,008797	0,036812	-3,31261
SFRP1	0,157398	-0,00456	2,399884	0,020702	0,064501	-4,08376
Bak	0,154867	-0,01284	2,479593	0,017057	0,056989	-3,91092
PDHK1	0,15269	0,068548	2,646183	0,011251	0,041448	-3,53617
PRAS40	0,147202	0,046683	2,524564	0,015268	0,054836	-3,81153
Creb	0,144895	0,013834	2,408974	0,020253	0,064501	-4,06426
E2F1	0,14419	0,059001	2,391116	0,021143	0,065089	-4,10251
Bad_pS112	0,134345	0,068923	2,098507	0,041638	0,106821	-4,69712
SOX17	0,133305	0,018711	2,026371	0,048818	0,117546	-4,83407
BAP1	0,129545	0,048736	2,397876	0,020802	0,064501	-4,08806
Ets-1	0,123696	-0,02815	2,277757	0,027657	0,078941	-4,34012
Hexokinase	0,122342	-0,02538	2,381739	0,021624	0,065204	-4,1225
DAPK1_pS:	0,104625	0,050536	2,042284	0,047148	0,116786	-4,80419

cut-off : P.Value<0.05

Table S9E. A11+Adav_vs_No.

ID	logFC	AveExpr	t	P.Value	adj.P.Val	B
Histone-H3	1,660532	0,365476	4,845047	1,61E-05	0,00099	2,782526
PAR	1,510213	0,33572	2,973889	0,004759	0,070328	-2,58996
Caspase-3-	1,396942	0,182884	4,993032	9,88E-06	0,00071	3,250265
Pyk2_pY40	-0,96877	-0,01398	-3,97781	0,000256	0,008491	0,140618
H2AX_pS13	0,893769	0,008345	4,270372	0,000103	0,00402	1,009495
cdc2_pY15	-0,87577	0,010496	-9,91986	8,61E-13	3,71E-10	18,94938
Hif-1-alpha	-0,85022	-0,06664	-5,18395	5,25E-06	0,000452	3,858932
CDK1_pT14	0,844253	-0,00726	2,951311	0,005058	0,070328	-2,64583
p38-MAPK-	0,823227	-0,03056	3,026283	0,004128	0,068422	-2,45923
H2AX_pS14	0,790435	0,173534	4,534002	4,42E-05	0,002117	1,813286
ER-a_pS11	0,790237	-0,08919	2,959113	0,004953	0,070328	-2,62656
DM-Histon	0,75352	0,167256	5,258556	4,09E-06	0,000441	4,098187
Rad51	-0,75204	0,041184	-3,2868	0,001996	0,039113	-1,7879
DUSP6	-0,717	0,009088	-2,52538	0,015237	0,164176	-3,64407
Chk1	-0,70422	0,162593	-3,67296	0,000647	0,014672	-0,73396
Connexin-4	-0,69722	-0,0405	-2,254	0,02923	0,237703	-4,21971
PHLPP	-0,64405	0,013041	-3,00953	0,00432	0,068966	-2,50118
Caspase-7-	0,627079	0,19787	4,550612	4,19E-05	0,002117	1,86451
Snail	-0,61763	0,250236	-2,37936	0,021747	0,203763	-3,96003
MMP14	-0,5732	0,114206	-2,84508	0,006717	0,087728	-2,90488
RBM15	0,549739	0,077333	3,778706	0,000471	0,012677	-0,43449
MLH1	0,469404	0,049452	4,490071	5,09E-05	0,002194	1,678118
Akt1	-0,46457	-0,05668	-3,89089	0,000335	0,010298	-0,11219
KAP1	0,457076	0,102915	2,60094	0,012616	0,146961	-3,47509
SOD1	0,449312	0,07373	5,615004	1,24E-06	0,000178	5,25006
Akt2	-0,44902	-0,17563	-3,73922	0,00053	0,013442	-0,54683
Bcl2	0,424827	0,084614	4,148096	0,000151	0,005416	0,643143
Stathmin-1	0,403128	-0,0511	2,188235	0,034009	0,262066	-4,3515
EMA	-0,39344	0,022802	-3,70931	0,00058	0,013889	-0,63151
Annexin-I	0,357552	0,056642	3,133002	0,003076	0,055247	-2,18841
RPA32	-0,34467	-0,0536	-2,71053	0,009543	0,117518	-3,22361
IGF1R_pY1	-0,33658	0,123006	-2,28208	0,027379	0,231375	-4,16249
Mcl-1	-0,33517	-0,13578	-2,58973	0,012977	0,147185	-3,50038
Rictor_pT1	-0,32518	-0,05303	-2,13624	0,038264	0,280358	-4,4535
PARP	-0,31278	-0,03808	-2,87949	0,006131	0,082578	-2,82167
SHP2	-0,29082	-0,06484	-5,80895	6,45E-07	0,000139	5,881525
Erk5	-0,28423	-0,12646	-3,21449	0,00245	0,045903	-1,97766
S6	0,283645	0,054635	2,309028	0,025701	0,226063	-4,10707
4E-BP1_pT	-0,27786	-0,04807	-2,03849	0,047541	0,315236	-4,63982
Caspase-8	0,269812	0,089702	3,412426	0,001392	0,029351	-1,45229
HER3_pY12	-0,26268	0,046123	-3,85302	0,000376	0,01079	-0,22148
DDB-1	-0,25911	0,062349	-2,29022	0,026862	0,231375	-4,14581
DRP1	-0,24934	-0,07825	-2,41783	0,019825	0,189875	-3,87817
ERRalpha	-0,24313	-0,0124	-2,06867	0,044487	0,299593	-4,58305
PKCa	-0,23768	-0,16464	-2,61958	0,012037	0,144105	-3,43285
B-Raf_pS44	-0,2342	-0,04863	-2,35566	0,023014	0,206645	-4,00996
Raptor	-0,23191	-0,08896	-3,40317	0,00143	0,029351	-1,47726
Lasu1	-0,22083	-0,10668	-2,23452	0,03058	0,244071	-4,25908
XPA	0,212115	-0,00526	2,120066	0,039679	0,280358	-4,48481

ATP5A	0,20923	0,072204	2,995605	0,004487	0,06907	-2,53596
Annexin-VI	0,207886	0,064325	3,048624	0,003883	0,066941	-2,40303
GAPDH	-0,19961	-0,02123	-2,17838	0,034782	0,262999	-4,37098
Atg4B	-0,18483	-0,10914	-2,49903	0,016261	0,170027	-3,70213
IRF-1	0,179682	0,061071	2,360705	0,022739	0,206645	-3,99936
Bad_pS112	0,177552	0,068923	2,773425	0,008108	0,102776	-3,07596
SF2	0,17565	0,101404	2,491419	0,016569	0,170027	-3,71882
C-Raf	-0,16826	0,036654	-2,43318	0,019101	0,187105	-3,84523
CSK	-0,15892	-0,05276	-2,07448	0,04392	0,299593	-4,57205
SOD2	0,14946	0,022023	2,089779	0,042454	0,295126	-4,54294
TUFM	-0,14601	-0,08513	-2,26948	0,028196	0,233703	-4,18824
Syk	0,13419	0,060814	2,534873	0,014882	0,164176	-3,62304
CD49b	0,129286	0,083165	2,122811	0,039436	0,280358	-4,47951
Collagen-V	0,12828	0,014401	2,187705	0,03405	0,262066	-4,35255
DAPK1_pS3	0,12577	0,050536	2,455041	0,018112	0,181543	-3,79804
Rheb	0,109917	0,01574	2,128986	0,038893	0,280358	-4,46756
BAP1	0,109289	0,048736	2,022942	0,049185	0,321193	-4,6688

cut-off : P.Value<0.05

Table S10: Antibodies used for RPPA analysis of *MMTV-R26^Met^* treated cells

#	Official Ab Name	Ab Name Reported on Dataset	Gene Name	Company	Catalog #	Species	RPPA Dilution
1	14-3-3 beta	14-3-3-beta	YWHAB	Santa Cruz	sc-628	Rabbit	1:75
2	14-3-3 epsilon	14-3-3-epsilon	YWHAE	Santa Cruz	sc-23957	Mouse	1:50
3	14-3-3 zeta	14-3-3-zeta	YWHAZ	Santa Cruz	sc-1019	Rabbit	1:5000
4	4E-BP1	4E-BP1	EIF4EBP1	CST	9452	Rabbit	1:100
5	4E-BP1 (phospho S65)	4E-BP1_pS65	EIF4EBP1	CST	9456	Rabbit	1:250
6	4E-BP1 (phospho T37/46)	4E-BP1-pT37-T46	EIF4EBP1	CST	9459	Rabbit	1:2000
7	53BP1	53BP1	TP53BP1	CST	4937	Rabbit	1:300
8	A1Up	UBQLN4	UBQLN4	Santa Cruz	sc-136145	Mouse	1:125
9	Acetyl-CoA-Carboxylase	ACC1	ACACA, B	Epitomics/ Abcam	1768-1/ ab45174	Rabbit	1:1500
10	Acetyl-CoA-Carboxylase (phospho S79)	ACC_pS79	ACACA, B	CST	3661	Rabbit	1:500
11	ACSL1 (D2H5)	ACSL1	ACSL1	CST	9189	Rabbit	1:500
12	ACVRL1	ACVRL1	ACVRL1	Epitomics/ Abcam	2940-1/ ab108207	Rabbit	1:30
13	ADAR1	ADAR1	ADAR	Abcam	ab88574	Mouse	1:100
14	Akt	Akt	AKT1, 2, 3	CST	4691	Rabbit	1:7500
15	Akt (phospho S473)	Akt_pS473	AKT1, 2, 3	CST	9271	Rabbit	1:150
16	Akt (phospho T308)	Akt_pT308	AKT1, 2, 3	CST	2965	Rabbit	1:250
17	Akt1	Akt1	AKT1	CST	2938	Rabbit	1:1000
18	Akt1 (phospho S473)	Akt1_pS473	AKT1	CST	9018	Rabbit	1:1000
19	Akt2	Akt2	AKT2	CST	3063	Rabbit	1:3000
20	Akt2 (phospho S474)	Akt2_pS474	AKT2	CST	8599	Rabbit	1:1000
21	Ambra1 (phospho S52)	Ambra1_pS52	AMBRA1	Millipore	ABC80	Rabbit	1:250
22	AMPK alpha 2 (phospho S345)	AMPK-a2_pS345	PRKAA1, 2	Abcam	ab129081	Rabbit	1:200
23	AMPKa	AMPKa	PRKAA1, 2	CST	2532	Rabbit	1:75

**24**	AMPKa (phospho T172)	AMPKa_pT172	PRKAA1, 2	CST	2535	Rabbit	1:100
**25**	Androgen Receptor (D6F11)	AR	AR	CST	5153	Rabbit	1:250
**26**	Annexin I	Annexin-I	ANXA1	BD Biosciences	610066	Mouse	1:5000
**27**	Annexin VII	Annexin-VII	ANXA7	BD Biosciences	610668	Mouse	1:20
**28**	A-Raf	A-Raf	ARAF	CST	4432	Rabbit	1:200
**29**	A-Raf (phospho S299)	A-Raf_pS299	ARAF	CST	4431	Rabbit	1:25
**30**	ARID1A	ARID1A	ARID1A	Sigma-Aldrich	HPA005456	Rabbit	1:1000
**31**	ASNS	ASNS	ASNS	Sigma-Aldrich	HPA029318	Rabbit	1:500
**32**	Atg3	Atg3	ATG3	CST	3415	Rabbit	1:72
**33**	Atg4B	Atg4B	ATG4B	CST	13507	Rabbit	1:200
**34**	Atg5	Atg5	ATG5	CST	12994	Rabbit	1:1000
**35**	Atg7	Atg7	ATG7	CST	8558	Rabbit	1:1000
**36**	ATM	ATM	ATM	CST	2873	Rabbit	1:250
**37**	ATM (phospho S1981)	ATM_pS1981	ATM	CST	5883	Rabbit	1:20
**38**	ATP5A	ATP5A	ATP5A	Abcam	ab14748	Mouse	1:500
**39**	ATP5H	ATP5H	ATP5H	Abcam	ab110275	Mouse	1:30
**40**	ATR	ATR	ATR	CST	2790	Rabbit	1:30
**41**	ATR (phospho S428)	ATR_pS428	ATR	Abcam	ab178407	Rabbit	1:1000
**42**	ATRX	ATRX	ATRX	Abcam	ab97508	Rabbit	1:300
**43**	Aurora B/AIM1	Aurora-B	AURKB	CST	3094	Rabbit	1:38
**44**	Axl	Axl	AXL	CST	8661	Rabbit	1:500
**45**	B7-H3	B7-H3	CD276	CST	14058	Rabbit	1:200
**46**	B7-H4	B7-H4	VTCN1	CST	14572	Rabbit	1:50
**47**	Bad (phospho S112)	Bad_pS112	BAD	CST	9291	Rabbit	1:50
**48**	Bak	Bak	BAK1	Epitomics/ Abcam	1542-1/ ab32371	Rabbit	1:400
**49**	BAP1	BAP1	BAP1	Santa Cruz	sc-28383	Mouse	1:200
**50**	Bax	Bax	BAX	CST	2772	Rabbit	1:100
**51**	b-Catenin	b-Catenin	CTNNB1	CST	9562	Rabbit	1:1500
**52**	Bcl2	Bcl2	BCL2	Dako	M0887	Mouse	1:50
**53**	Bcl2A1	Bcl2A1	BCL2A1	Abnova	PAB8528	Rabbit	1:250
**54**	Bcl-xL	Bcl-xL	BCL2L1	CST	2762	Rabbit	1:100

**55**	Beclin 1	Beclin	BECN1	ThermoFisher	PA1-16857	Rabbit	1:500
**56**	beta Actin	b-Actin	ACTB	CST	4970	Rabbit	1:50
**57**	beta Catenin (phospho T41/S45)	b-Catenin_pT41_S45	CTNNB1	CST	9565	Rabbit	1:30
**58**	Bid	Bid	BID	CST	2002	Rabbit	1:500
**59**	Bim (C34C5)	Bim	BCL2L11	Epitomics/ Abcam	1036-1/ ab32158	Rabbit	1:400
**60**	BiP/GRP78	BiP-GRP78	HSPA5	BD Biosciences	610978	Mouse	1:150
**61**	BMK1/Erk5 (phospho T218/Y220)	BMK1-Erk5_pT218_Y220	MAPK7	Millipore	07-507	Rabbit	1:500
**62**	B-Raf	B-Raf	BRAF	CST	14814	Rabbit	1:500
**63**	B-Raf (phospho S445)	B-Raf_pS445	BRAF	CST	2696	Rabbit	1:75
**64**	BRD4	BRD4	BRD4	CST	13440	Rabbit	1:1000
**65**	CA9 (CAIX)	CA9	CA9	CST	5649	Rabbit	1:200
**66**	c-Abl	c-Abl	ABL1	CST	2862	Rabbit	1:100
**67**	c-Abl (phospho Y412)	Abl_pY412	ABL1	CST	2865	Rabbit	1:200
**68**	Caspase 3 (cleaved asp175)	Caspase-3-cleaved	CASP3	CST	9661	Rabbit	1:500
**69**	Caspase 7 (cleaved)	Caspase-7-cleaved	CASP7	CST	9491	Rabbit	1:60
**70**	Caspase 8	Caspase-8	CASP8	CST	9746	Mouse	1:150
**71**	Caspase 8 (cleaved asp391)	Caspase-8-cleaved	CASP8	CST	9496	Rabbit	1:500
**72**	Caspase-3	Caspase-3	CASP3	Epitomics/ Abcam	1476-1/ ab32042	Rabbit	1:250
**73**	Caveolin 1	Caveolin-1	CAV1	CST	3238	Rabbit	1:3000
**74**	CD134/OX40	CD134	TNFRSF	Abcam	ab76000	Rabbit	1:100
**75**	CD171 (L1)	CD171	L1CAM	Biolegend	826701	Mouse	1:1000
**76**	CD20	CD20	MS4A1	Epitomics/ Abcam	1632-1/ ab78237	Rabbit	1:75
**77**	CD26	CD26	DPP4	Abcam	ab28340	Rabbit	1:1000
**78**	CD29	CD29	ITGB1	BD Biosciences	610467	Mouse	1:30
**79**	CD31	CD31	PECAM1	Dako/ Fisher	M0823/ MS353S	Mouse	1:25
**80**	CD38	CD38	CD38	Abcam	ab108403	Rabbit	1:250
**81**	CD4	CD4	CD4	Abcam	ab133616	Rabbit	1:500

**82**	CD44	CD44	CD44	CST	3570	Mouse	1:20
**83**	CD45	CD45	CD45	DAKO/ ThermoFisher	M070129-2/ MS355P	Mouse	1:1000
**84**	CD49b	CD49b	ITGA2	BD Biosciences	611016	Mouse	1:50
**85**	CD86	CD86	CD86	Abcam	ab53004	Rabbit	
**86**	Cdc2 (phospho Y15)	cdc2_pY15	CDK	CST	4539	Rabbit	1:38
**87**	cdc25C	cdc25C	CDC25C	CST	4688	Rabbit	1:250
**88**	CDK1/2/3 (phospho T14)	CDK1_pT14	CDK1, 2, 3	Abcam	ab32384	Rabbit	1:1000
**89**	CDKN2A/p16INK4 a	p16INK4a	CDKN2A	Abcam	ab81278	Rabbit	1:500
**90**	Chk1	Chk1	CHEK	CST	2360	Mouse	1:100
**91**	Chk1 (phospho S296)	Chk1_pS296	CHEK1	Abcam	ab79758	Rabbit	1:125
**92**	Chk1 (phospho S345)	Chk1_pS345	CHEK1	CST	2348	Rabbit	1:30
**93**	Chk2	Chk2	CHEK2	CST	3440	Mouse	1:50
**94**	Chk2 (phospho T68)	Chk2_pT68	CHEK2	CST	2197	Rabbit	1:250
**95**	c-IAP2	c-IAP2	BIRC3	CST	3130	Rabbit	1:50
**96**	CIITA	CIITA	CIITA	CST	3793	Rabbit	1:250
**97**	c-Jun (phospho S73)	c-Jun_pS73	JUN	CST	9164	Rabbit	1:30
**98**	c-Kit	c-Kit	KIT	Epitomics/ Abcam	1522-1/ ab32363	Rabbit	1:250
**99**	Claudin 7	Claudin-7	CLDN7	Abcam	ab79481	Rabbit	1:250
**100**	c-Myc	c-Myc	MYC	Santa Cruz	sc-764	Rabbit	1:250
**101**	COG3	COG3	COG3	ProteinTech	11130-1-AP	Rabbit	1:750
**102**	Collagen-VI/COL6A1	Collagen-VI	COL6A1	Santa Cruz	sc-20649	Rabbit	1:6000
**103**	Complex II Subunit	Complex-II-Subunit	SDHB	Life Technologies	459230	Mouse	1:200
**104**	Connexin 43	Connexin-43	GJA1	CST	3512	Rabbit	1:150
**105**	Coup-TFII	Coup-TFII	NR2F2	CST	6434	Rabbit	1:50
**106**	Cox2	Cox2	PTGS2	CST	4842	Rabbit	1:75
**107**	Cox-IV	Cox-IV	COX4I1	CST	4850	Rabbit	1:5000
**108**	C-Raf	C-Raf	RAF1	Millipore	04-739	Rabbit	1:100
**109**	C-Raf (phospho S338)	C-Raf_pS338	RAF1	CST	9427	Rabbit	1:200

**110**	Creb	Creb	CREB1	CST	9197	Rabbit	1:75
**111**	CSK	CSK	CSK	CST	4980	Rabbit	1:300
**112**	CtIP	CtIP	RBBP8	CST	9201	Rabbit	1:500
**113**	Cyclin B1	Cyclin B1	CCNB1	Epitomics/ Abcam	1495-1/ ab32053	Rabbit	1:1500
**114**	Cyclin D1	Cyclin-D1	CCND1	Millipore Sigma	SAB4502603	Rabbit	1:200
**115**	Cyclin D3	Cyclin D3	CCND3	CST	2936	Mouse	1:1000
**116**	Cyclin E1	Cyclin E1	CCNE1	Santa Cruz	sc-247	Mouse	1:25
**117**	Cyclophilin-F	Cyclophilin-F	PPIF	Abcam	MSA04/ ab110324	Mouse	1:50000
**118**	Cytokeratin 19	Cytokeratin-19	KRT19	Dako	M0888	Mouse	1:50
**119**	DAP Kinase 1 (phospho S308)	DAPK1_pS308	DAPK1	GeneTex	GTX10524	Mouse	1:200
**120**	DAP Kinase 2	DAPK2	DAPK2	Abcam	ab51601	Rabbit	1:250
**121**	DDB-1	DDB-1	DDB1	CST	6998	Rabbit	1:5000
**122**	Detyrosinated alpha-Tubulin	D-a-Tubulin	TUBA4A, TUBA3C	Abcam	ab48389	Rabbit	1:1500
**123**	Di-Methyl-Histone H3 (Lys4/C64G9)	DM-Histone-H3	HIST1H3A	CST	9725	Rabbit	1:100
**124**	Dimethyl-K9 Histone H3	DM-K9-Histone-H3	HIST3H3	Abcam	ab1220	Mouse	1:250
**125**	DNA Ligase IV	DNA-Ligase-IV	LIG4	CST	14649	Rabbit	1:1000
**126**	DNA Polymerase gamma (D1Y6R)	POLG	POLG	CST	13609	Rabbit	1:500
**127**	DNMT1 (D63A6)	DNMT1	DNMT1	CST	5032	Rabbit	1:500
**128**	DRP1 (D8H5)	DRP1	DNM1L	CST	5391	Rabbit	1:1000
**129**	DUSP4/MKP2	DUSP4	DUSP4	CST	5149	Rabbit	1:150
**130**	DUSP6	DUSP6	DUSP6	Abcam	ab76310	Rabbit	1:750
**131**	Dvl3	Dvl3	DVL3	CST	3218	Rabbit	1:30
**132**	E2F1	E2F1	E2F1	Santa Cruz	sc-251	Mouse	1:20
**133**	E-Cadherin	E-Cadherin	CDH1	CST	3195	Rabbit	1:150
**134**	eEF2	eEF2	EEF2	CST	2332	Rabbit	1:50
**135**	eEF2K	eEF2K	EEF2K	CST	3692	Rabbit	1:50
**136**	EGFR	EGFR	EGFR	CST	2232	Rabbit	1:75
**137**	EGFR (phospho Y1173)	EGFR_pY1173	EGFR	Epitomics/ Abcam	1124-1/ ab32578	Rabbit	1:300
**138**	eIF4E	eIF4E	EIF4E	CST	9742	Rabbit	1:75
**139**	eIF4E (phospho S209)	eIF4E_pS209	EIF4E	Abcam	ab76256	Rabbit	1:250

**140**	eIF4G	eIF4G	EIF4G1	CST	2498	Rabbit	1:1000
**141**	Elk1 (phospho S383)	Elk1_pS383	ELK1	CST	9181	Rabbit	1:50
**142**	Enolase-2 (D20H2)	Enolase-2	ENO2	CST	8171	Rabbit	1:250
**143**	ENY2	ENY2	ENY2	GeneTex	GTX629542	Mouse	1:500
**144**	Eph Receptor A2	EPHA2	EPHA2	Abcam	ab133501	Rabbit	1:1000
**145**	Epithelial Membrane Antigen	EMA	MUC1	DAKO	M061329-2	Mouse	1:750
**146**	ErbB3/HER3	HER3	ERBB3	Santa Cruz	sc-285	Rabbit	1:300
**147**	ErbB3/HER3 (phospho Y1289)	HER3_pY1289	ERBB3	CST	4791	Rabbit	1:50
**148**	ERCC1	ERCC1	ERCC1	Santa Cruz	sc-17809	Mouse	1:38
**149**	Erk5	Erk5	MAPK7	CST	3552	Rabbit	1:500
**150**	ERRalpha (E1G1J)	ERRalpha	ESRRA	CST	13826	Rabbit	1:500
**151**	ERRFI1/MIG6	MIG6	ERRFI1	Sigma-Aldrich	WH0054206 M1	Mouse	1:50
**152**	Estrogen Receptor	ER	ESR1	Lab Vision	RM-9101	Rabbit	1:40
**153**	Estrogen Receptor alpha	ER-a	ERSA	CST	13258	Rabbit	1:500
**154**	Estrogen Receptor alpha (phospho S118)	ER-a_pS118	ESR1	Epitomics/ Abcam	1091-1/ ab32396	Rabbit	1:500
**155**	Ets-1	Ets-1	ETS1	Bethyl	A303-501A	Rabbit	1:100
**156**	FAK	FAK	PTK2	Epitomics/ Abcam	1700-1/ ab40794	Rabbit	1:1000
**157**	FAK (phospho Y397)	FAK_pY397	PTK2	CST	3283	Rabbit	1:25
**158**	Fatty Acid Synthase	FASN	FASN	CST	3180	Rabbit	1:1000
**159**	FGF-basic	FGF-basic	FGF2	VWR	10775-082 (500-P18)	Rabbit	1:1000
**160**	Fibronectin	Fibronectin	FN1	Epitomics	1574-1	Rabbit	1:10000
**161**	FoxM1	FOXM1	FOXM1	CST	5436	Rabbit	1:30
**162**	FoxO3a	FoxO3a	FOXO3	CST	2497	Rabbit	1:20
**163**	FoxO3a (phospho S318/S321)	FoxO3a_pS318_S321	FOXO3	CST	9465	Rabbit	1:30
**164**	FRS2-a (phospho Y196)	FRS2-a_pY196	FRS2	CST	3864	Rabbit	1:100
**165**	G6PD	G6PD	G6PD	Santa Cruz	sc-373887	Mouse	1:75

**166**	Gab2	Gab2	GAB2	CST	3239	Rabbit	1:300
**167**	GAPDH	GAPDH	GAPDH	Ambion/ Invitrogen	AM4300	Mouse	1:75000
**168**	GATA3	GATA3	GATA3	BD Biosciences	558686	Mouse	1:150
**169**	GATA6	GATA6	GATA6	CST	5851	Rabbit	1:200
**170**	GCLC	GCLC	GCLC	Proteintech Group	12601-1-AP	Rabbit	1:500
**171**	GCLM	GCLM	GCLM	Abcam	ab124827	Rabbit	1:500
**172**	GCN5L2	GCN5L2	KAT2A	CST	3305	Rabbit	1:30
**173**	Gli1	Gli1	GLI1	CST	3538	Rabbit	1:3000
**174**	Gli3	Gli3	GLI3	Abcam	ab69838	Rabbit	1:1000
**175**	Glucose-6 Phosphate Dehydrogenase	G6PD	G6PD	CST	8866	Rabbit	1:30
**176**	Glutamate Dehydrogenase1/ 2	Glutamate-D1-2	GLUD1	Novus	NBP2-16679	Rabbit	1:500
**177**	Glutaminase	Glutaminase	GLS	Abcam	ab156876	Rabbit	1:150
**178**	Glycogen Synthase	Gys	GYS1	CST	3886	Rabbit	1:2000
**179**	Glycogen Synthase (phospho S641)	Gys_pS641	GYS1	CST	3891	Rabbit	1:300
**180**	GPBB	GPBB	PYGM	Novus	NBP1-32799	Rabbit	1:200
**181**	Granzyme B	Granzyme-B	GZMB	CST	4275	Rabbit	1:500
**182**	GRB7	GRB7	GRB7	Abcam	ab183737	Rabbit	1:500
**183**	Grp75 (D13H4)	Grp75	HSPA9	CST	3593	Rabbit	1:250
**184**	GSK-3alpha/beta	GSK-3a-b	GSK3A, B	Santa Cruz	sc-7291	Mouse	1:750
**185**	GSK-3alpha/beta (phospho S21/S9)	GSK-3a-b_pS21_S9	GSK3A, B	CST	9331	Rabbit	1:200
**186**	GSK-3B	GSK-3B	GSK3B	CST	9315	Rabbit	1:750
**187**	GSK-3beta (phospho S9)	GSK-3b_pS9	GSK3B	CST	5558	Rabbit	1:250
**188**	H2AX (phospho S140)	H2AX_pS140	H2AFX	Pierce Biotechnolog y	MA12022	Mouse	1:100
**189**	Hamartin/TSC1	TSC1	TSC1	CST	4906	Rabbit	1:200
**190**	HER2	HER2	ERBB2	Lab Vision	MS-325-P1	Mouse	1:300
**191**	HER2 (phospho Y1248)	HER2_pY1248	ERBB2	R&D systems	AF1768	Rabbit	1:1500
**192**	Heregulin	Heregulin	NRG1	CST	2573	Rabbit	01:30

**193**	HES1	HES1	HES1	CST	11988	Rabbit	1:500
**194**	Hexokinase II	Hexokinase II	HK2	CST	2106	Rabbit	1:100
**195**	Hif-1-alpha	Hif-1-alpha	HIF1A	BD Biosciences	610958	Mouse	1:20
**196**	Histone H3	Histone H3	HIST3H3	Abcam	ab1791	Rabbit	1:5000
**197**	HLA-DQA1	HLA-DQA1	HLA-DQA1	Abcam	ab128959	Rabbit	1:3000
**198**	HLA-DR/DP/DQ/DX	HLA-DR-DP-DQ-DX	HLA-DRA	Santa Cruz	sc-53302	Mouse	1:250
**199**	HMHA1	HMHA1	HMHA1	ProteinTech	14832-1-AP	Rabbit	1:3000
**200**	HSP27	HSP27	HSBP1	CST	2402	Mouse	1:75
**201**	HSP27 (phospho S82)	HSP27_pS82	HSBP1	CST	2401	Rabbit	1:75
**202**	HSP60	HSP60	HSP60	CST	12165	Rabbit	1:1000
**203**	HSP70	HSP70	HSPA1A	CST	4872	Rabbit	1:50
**204**	Hsp75/TRAP1	TRAP1	TRAP1	BD Biosciences	612344	Mouse	1:750
**205**	IDO	IDO	IDO1	CST	86630	Rabbit	1:200
**206**	IGF1R (phospho Y1135/Y1136	IGF1R_pY1135_Y1136	IGF1R, INSR	CST	3024	Rabbit	1:30
**207**	IGF-1Receptor beta	IGF1R-b	IGF1R	CST	3018	Rabbit	1:50
**208**	IGFBP2	IGFBP2	IGFBP2	CST	3922	Rabbit	1:50
**209**	IGFBP3	IGFBP3	IGFBP3	BD Biosciences	611504	Mouse	1:1000
**210**	IGFRb	IGFRb	IGF1R	CST	3027	Rabbit	1:250
**211**	IL-6	IL-6	IL6	CST	12153	Rabbit	1:250
**212**	INPP4b	INPP4b	INPP4B	CST	4039	Rabbit	01:30
**213**	Insulin Receptor beta	IR-b	INSR	CST	3025	Rabbit	1:100
**214**	IRF-1	IRF-1	IRF1	CST	8478	Rabbit	1:250
**215**	IRS1	IRS1	IRS1	Millipore	06-248	Rabbit	1:250
**216**	IRS2	IRS2	IRS2	CST	4502	Rabbit	1:100
**217**	JAB1	JAB1	COPS5	Santa Cruz	sc-13157	Mouse	1:30
**218**	Jagged1	Jagged1	JAG1	Abcam	ab109536	Rabbit	01:50
**219**	Jak2	Jak2	JAK2	CST	3230	Rabbit	1:750
**220**	JNK (phospho T183/Y185)	JNK_pT183_Y185	MAPK8	CST	4668	Rabbit	01:30
**221**	JNK2	JNK2	MAPK9	CST	4672	Rabbit	1:25
**222**	KAP1	KAP1	TRIM28	Abcam	ab10484	Rabbit	1:2000
**223**	KMT3A/HYPB/HIF-1	SETD2	SETD2	abcam	ab184190	Rabbit	1:1000

**224**	LAD1	LAD1	LAD1	Atlas	HPA028732	Rabbit	1:500
**225**	Lasu1/Ureb1	Lasu1	HUWE1	Bethyl	IHC-00439	Rabbit	1:1000
**226**	LC3A/B	LC3A-B	MAP1LC3A, B	CST	4108	Rabbit	1:250
**227**	Lck	Lck	LCK	CST	2752	Rabbit	1:75
**228**	LDHA	LDHA	LDHA	CST	3582	Rabbit	1:250
**229**	LRP6 (phospho S1490)	LRP6_pS1490	LRP6	CST	2568	Rabbit	1:250
**230**	MAPK (phospho T202/Y204)	MAPK_pT202/Y204	MAPK1, 3	CST	4377	Rabbit	1:25
**231**	Mcl-1	Mcl-1	MCL1	CST	5453	Rabbit	1:100
**232**	MDM2 (phospho S166)	MDM2_pS166	MDM2	CST	3521	Rabbit	1:60
**233**	MEK1	MEK1	MAP2K1	Epitomics/ Abcam	1235-1/ ab32576	Rabbit	1:1500
**234**	MEK1 (phospho S217/S221)	MEK1_p_S217/ S221	MAP2K1, 2	CST	9154	Rabbit	1:50
**235**	MEK2	MEK2	MAP2K2	CST	9125	Rabbit	1:50
**236**	MelanA	MelanA	MLANA	Abcam	ab51061	Rabbit	1:500
**237**	Melanoma gp100	Melan-gp100	PMEL	Abcam	ab137078	Rabbit	1:500
**238**	MERIT40	MERIT40	MERIT40	CST	12711	Rabbit	1:3000
**239**	MERIT40 (phospho S29)	MERIT40_pS29	BABAM1	CST	12110	Rabbit	1:300
**240**	Merlin/NF2	Merlin	NF2	Novus	22710002	Rabbit	1:250
**241**	MIF	MIF	MIF	Santa Cruz	sc-130329	Rabbit	1:100
**242**	MITF (D5G7V)	MITF	MITF	CST	12590	Rabbit	1:500
**243**	Mitofusin-1	Mitofusin-1	MFN1	CST	14739	Rabbit	1:500
**244**	Mitofusin-2	Mitofusin-2	MFN2	CST	11925	Rabbit	1:1000
**245**	MLH1 (4C9C7)	MLH1	MLH1	CST	3515	Mouse	1:500
**246**	MLKL	MLKL	MLKL	CST	14993	Rabbit	1:1000
**247**	MMP2	MMP2	MMP2	CST	4022	Rabbit	1:75
**248**	Mnk1	Mnk1	MKNK1	CST	2195	Rabbit	1:750
**249**	Monocarboxylic Acid Transporter 4	MCT4	SLC16A4	Millipore	AB3314P	Rabbit	1:500
**250**	MR1	MR1	MR1	Santa Cruz	sc-377312	Mouse	1:500
**251**	MRAP	MRAP	MRAP	Abcam	ab103319	Rabbit	1:500
**252**	MSH2 (D24B5)	MSH2	MSH2	CST	2017	Rabbit	1:750
**253**	MSH6	MSH6	MSH6	Novus	22030002	Rabbit	1:1000
**254**	MSI2 (EP1305Y)	MSI2	MSI2	Abcam	ab76148	Rabbit	1:1000

**255**	MTCO1	MTCO1	MTCO1	Abcam	ab14705	Mouse	1:500
**256**	mTOR	mTOR	MTOR	CST	2983	Rabbit	1:3000
**257**	mTOR (phospho S2448)	mTOR_pS2448	MTOR	CST	2971	Rabbit	1:50
**258**	MTSS1	MTSS1	MTSS1	Novus	H00009788-M01A	Mouse	1:250
**259**	Myosin Heavy Chain 11	Myosin-11	MYH11	Novus	21370002	Rabbit	1:1000
**260**	Myosin IIa	Myosin-IIa	MYH9	CST	3403	Rabbit	1:1000
**261**	Myosin IIa (phospho S1943)	Myosin-IIa_pS1943	MYH9	CST	5026	Rabbit	1:750
**262**	Myt1	Myt1	PKMYT1	CST	4282	Rabbit	1:100
**263**	NAPSIN-A	NAPSIN-A	NAPSA	Epitomics/ Abcam	5795-1/ ab129189	Rabbit	1:150
**264**	N-Cadherin	N-Cadherin	CDH2	CST	4061	Rabbit	1:25
**265**	NDRG1 (phospho T346)	NDRG1_pT346	NDRG1	CST	3217	Rabbit	01:50
**266**	NDUFB4	NDUFB4	NDUFB4	Abcam	ab110243	Mouse	1:25
**267**	NF-kB p65 (phospho S536)	NF-kB-p65_pS536	RELA	CST	3033	Rabbit	1:30
**268**	Notch1	Notch1	NOTCH1	CST	3268	Rabbit	01:30
**299**	Notch1 (Cleaved)	Notch1-cleaved	NOTCH1	CST	4147	Rabbit	1:100
**270**	Notch3	Notch3	NOTCH3	Novus	H00004854-M01	Mouse	1:250
**271**	NQO1	NQO1	NQO1	CST	3187	Mouse	1:15000
**272**	N-Ras	N-Ras	NRAS	Santa Cruz	sc-31	Mouse	1:50
**273**	NRF2	NRF2	NRF2	CST	12721	Rabbit	1:500
**274**	Oct-4	Oct-4	POU5F1	CST	2750	Rabbit	1:40
**275**	p16/INK4a	p16-INK4a	CDKN2A	Epitomics/ Abcam	1712-1/ ab40803	Rabbit	1:500
**276**	p21	p21	CDKN1A	Santa Cruz	sc-6246	Rabbit	1:150
**277**	p27 (phospho T157)	p27_pT157	CDKN1B	R&D Systems	AF1555	Rabbit	1:30
**278**	p27 (phospho T198)	p27_pT198	CDKN1B	Abcam	ab64949	Rabbit	01:50
**279**	p27 KIP 1	p27-Kip-1	CDKN1B	Epitomics/ Abcam	1591-1/ ab32034	Rabbit	01:40
**280**	p38 (phospho T180/Y182)	p38_pT180_Y182	MAPK11, 12, 13, 14	CST	9211	Rabbit	1:38
**281**	p38 alpha MAPK	p38-a	MAPK1	CST	9228	Mouse	1:300
**282**	p38 MAPK	p38-MAPK	MAPK11, 12, 14	CST	9212	Rabbit	1:1500

**283**	p38/MAPK (phospho T180/Y182)	p38-MAPK_pT180_Y182	MAPK14	CST	9215	Rabbit	1:250
**284**	p44/42 MAPK	p44-42-MAPK	MAPK1, 3	CST	4695	Rabbit	1:2000
**285**	p53	p53	TP53	CST	9282	Rabbit	1:2500
**286**	p70 S6 Kinase (phospho T389)	p70-S6K_pT389	RPS6KB1	CST	9205	Rabbit	1:50
**287**	p70/S6K1	p70-S6K1	RPS6KB1	Epitomics/ Abcam	1494-1/ ab32529	Rabbit	1:300
**288**	p90RSK (phospho T573)	p90RSK_pT573	RPS6K	CST	9346	Rabbit	1:25
**289**	PAI-1	PAI-1	SERPINE1	BD Biosciences	612024	Mouse	1:50
**290**	PAICS	PAICS	PAICS	Sigma-Aldrich	HPA035895	Rabbit	1:250
**291**	PAK1	PAK1	PAK1	CST	2602	Rabbit	1:750
**292**	PAK4	PAK4	PAK4	CST	3242	Rabbit	1:300
**293**	PAR	PAR	PAR	Trevigen	4336-BPC-100	Rabbit	1:30000
**294**	PARG	PARG	PARG	CST	66564	Rabbit	1:1000
**295**	PARK7/DJ1	DJ1	PARK7	Abcam	ab76008	Rabbit	1:5000
**296**	PARP	PARP	PARP1	CST	9532	Rabbit	1:1000
**297**	Patched	Patched	PTCH1	Abcam	ab53715	Rabbit	1:1000
**298**	Paxillin	Paxillin	PXN	CST	2542	Rabbit	1:250
**299**	P-Cadherin	P-Cadherin	CDH3	CST	2130	Rabbit	1:38
**300**	PCNA	PCNA	PCNA	CST	2586	Mouse	1:250
**301**	PD-1	PD-1	PDCD1	CST	43248	Mouse	1:500
**302**	Pdcd4	Pdcd4	PDCD4	Rockland	600-401-965	Rabbit	1:750
**303**	PDGFRB	PDGFR-b	PDGFRB	Invitrogen	MA5-15143	Rabbit	1:500
**304**	PDH	PDH	PDH	Abcam	ab110332	Mouse	1:100
**305**	PDHK1	PDHK1	PDHK1	CST	3820	Rabbit	1:300
**306**	PDK1	PDK1	PDPK1	CST	3062	Rabbit	01:50
**307**	PDK1 (phospho S241)	PDK1_pS241	PDPK1	CST	3061	Rabbit	01:50
**308**	PD-L1	PD-L1	CD274	CST	13684	Rabbit	1:250
**309**	PEA-15	PEA-15	PEA15	CST	2780S	Rabbit	1:100
**310**	PED/PEA-15 (phospho S116)	PEA-15_pS116	PEA15	Life Technologies	44836G	Rabbit	1:100
**311**	PHGDH	PHGDH	PHGDH	CST	13428	Rabbit	1:1000
**312**	PI3 Kinase p110 alpha	PI3K-p110-a	PIK3CA	CST	4255	Rabbit	1:50
**313**	PI3K p110 beta	PI3K-p110-b	PIK3BC	Santa Cruz	sc-376412	Mouse	1:40

**314**	PI3K p85	PI3K-p85	PIK3R1	Millipore	06-195	Rabbit	1:15000
**315**	PKA RI alpha	PKA-a	PRKAR1A	CST	5675	Rabbit	1:250
**316**	PKC alpha/beta II (phospho T638/641)	PKC-a-b-II_pT638_T641	PRKCA, B	CST	9375	Rabbit	1:1000
**317**	PKC(pan) beta II (phospho S660)	PKC-b-II_pS660	PRKCA, B, D, E, H, Q	CST	9371	Rabbit	1:200
**318**	PKC delta (phospho S664)	PKC-delta_pS664	PRKCD	Millipore	07-875	Rabbit	1:75
**319**	PKCalpha	PKCa	PRKCA	CST	2056	Rabbit	1:200
**320**	PKM2	PKM2	PKM	CST	4053	Rabbit	1:300
**321**	PLC gamma2 (phospho Y759)	PLC-gamma2_pY759	PLCG2	CST	3874	Rabbit	01:25
**322**	PLK1	PLK1	PLK1	CST	4513	Rabbit	1:125
**323**	Met (phospho Y1234/Y1235)	c-Met_pY1234_Y1235	MET	CST	3129	Rabbit	1:100
**324**	PMS2	PMS2	PMS2	Novus Biologicals	22510002	Rabbit	1:1500
**325**	PRAS40	PRAS40	AKT1S1	Life Technologies	AHO1031	Mouse	1:75
**326**	PRAS40 (phospho T246)	PRAS40_pT246	AKT1S1	Life Technologies	441100G	Rabbit	1:500
**327**	PREX1	PREX1	PREX1	Abcam	ab102739	Rabbit	1:100
**328**	Progesterone Receptor [YR85]	PR	PGR	abcam	206926	Rabbit	1:500
**329**	PTEN	PTEN	PTEN	CST	9552	Rabbit	1:500
**330**	PTPN12	PTPN12	PTPN12	Abcam	ab76942	Rabbit	1:500
**331**	Puma	Puma	BBC3	CST	4976	Rabbit	1:50
**332**	PYGB	PYGB	PYGB	Sigma-Aldrich	SAB2900066	Rabbit	1:750
**333**	PYGM	PYGM	PYGM	Novus	H00005837-M10	Mouse	1:500
**334**	Pyk2 (phospho Y402)	Pyk2_pY402	PYK2	CST	3291	Rabbit	1:500
**335**	Pyruvate Dehydrogenase	PDHA1	PDHA1	CST	3205	Rabbit	1:200
**336**	Rab11	Rab11	RAB11A, B	CST	3539	Rabbit	1:30
**337**	Rab25	Rab25	RAB25	CST	4314	Rabbit	1:30
**338**	Rac1/Cdc42	Cdc42	CDC42	CST	4651	Rabbit	1:100
**339**	Rad23A	Rad23A	RAD23A	CST	24555	Rabbit	1:1000
**340**	Rad50	Rad50	RAD50	CST	3427	Rabbit	1:250
**341**	Rad51	Rad51	RAD51	Millipore	ABE257	Rabbit	1:1000

**342**	Raptor	Raptor	RPTOR	CST	2280	Rabbit	1:300
**343**	Rb	Rb	RB1	CST	9309	Mouse	1:150
**344**	Rb (phospho S807/811)	Rb_pS807_S811	RB1	CST	9308	Rabbit	1:1000
**345**	RBM15	RBM15	RBM15	Novus	21390002	Rabbit	1:5000
**346**	Rheb	Rheb	RHEB	R&D Systems	MAB3426	Mouse	1:75
**347**	Rictor	Rictor	RICTOR	CST	2114	Rabbit	1:100
**348**	Rictor (phospho T1135)	Rictor_pT1135	RICTOR	CST	3806	Rabbit	1:200
**349**	RIP	RIP	RIP	CST	4926	Rabbit	1:75
**350**	RIP3	RIP3	RIP3	CST	13526	Rabbit	1:500
**351**	RPA32 (phospho S4/S8)	RPA32_pS4/S8	RPA2	Bethyl	A300-245A	Rabbit	1:250
**352**	RPA32/RPA2	RPA32	RPA2	CST	2208	Rat	1:150
**353**	RRM1	RRM1	RRM1	CST	3388	Rabbit	1:100
**354**	RRM2	RRM2	RRM2	Life Technologies	PA527856	Rabbit	1:250
**355**	RSK	RSK	RPS6KA1, 2, 3	CST	9347	Rabbit	1:150
**356**	S100A4	S100A4	S100A4	CST	13018	Rabbit	1:1000
**357**	S6 (phospho S235/236)	S6_pS235_S236	RPS6	CST	2211	Rabbit	1:2500
**358**	S6 (phospho S240/244)	S6_pS240_S244	RPS6	CST	2215	Rabbit	1:1000
**359**	S6 Ribosomal Protein	S6	RPS6	CST	2317	Mouse	1:750
**360**	SCD	SCD	SCD	Santa Cruz	sc-58420	Mouse	1:20
**361**	SDHA	SDHA	SDHA	CST	11998	Rabbit	1:250
**362**	SFRP1	SFRP1	SFRP1	CST	4690	Rabbit	1:500
**363**	Shc_pY317	Shc_pY317	SHC1	CST	2431	Rabbit	01:25
**364**	SHP-2 (phospho Y542)	SHP-2_pY542	PTPN11	CST	3751	Rabbit	1:75
**365**	SHP2 / PTPN11	SHP2	PTPN11	CST	3397	Rabbit	1:250
**366**	SLC1A5	SLC1A5	SLC1A5	Sigma-Aldrich	HPA035240	Rabbit	1:150000
**367**	Slfn11	Slfn11	SLFN11	Santa Cruz	sc-374339	Mouse	1:150
**368**	Smac	Smac	DIABLO	CST	2954	Mouse	1:150
**369**	Smad1	Smad1	SMAD1	Epitomics/ Abcam	1649-1/ ab33902	Rabbit	1:500
**370**	Smad3	Smad3	SMAD3	Epitomics/ Abcam	1735-1/ ab40854	Rabbit	1:150
**371**	Smad4	Smad4	SMAD4	Santa Cruz	sc-7966	Mouse	1:30

**372**	Snail	Snail	SNAI1	CST	3895	Mouse	1:50
**373**	SOD1	SOD1	SOD1	CST	4266	Mouse	1:500
**374**	SOD2 (D9V9C)	SOD2	SOD2	CST	13194	Rabbit	1:200
**375**	Sox2	Sox2	SOX2	CST	2748	Rabbit	1:50
**376**	Src	Src	SRC	Millipore	05-184	Mouse	1:50
**377**	Src (phospho Y416)	Src_pY419	SRC	CST	2101	Rabbit	1:25
**378**	Src (phospho Y527)	Src_pY527	SRC	CST	2105	Rabbit	1:150
**379**	SRSF1/SF2	SF2	SRSF1	Invitrogen	324500	Mouse	1:75
**380**	Stat3	Stat3	STAT3	CST	4904	Rabbit	1:3000
**381**	Stat3 (phospho Y705)	Stat3_pY705	STAT3	CST	9145	Rabbit	1:100
**382**	Stat5a	Stat5a	STAT5A	Epitomics/ Abcam	1289-1/ ab32043	Rabbit	1:300
**383**	Stathmin-1	Stathmin-1	STMN1	Epitomics/ Abcam	1972-1/ ab52630	Rabbit	1:75
**384**	STING	STING	TMEM173	CST	13647	Rabbit	1:250
**385**	Syk	Syk	SYK	Santa Cruz	sc-1240	Mouse	1:500
**386**	Tau	Tau	MAPT	Millipore	05-348	Mouse	1:100
**387**	TAZ	TAZ	WWTR1	CST	4883	Rabbit	1:300
**388**	TFAM	TFAM	TFAM	CST	7495	Rabbit	1:300
**389**	Transferrin R	TFRC	TFRC	Novus	22500002	Rabbit	1:15000
**390**	TIGAR	TIGAR	TIGAR	Epitomics/ Abcam	S1711/ ab137573	Rabbit	1:100
**391**	Transglutaminase	Transglutaminase	TGM2	Lab Vision	MS-224-P1	Mouse	1:150
**392**	TRIM25	TRIM25	TRIM25	Abcam	ab167154	Rabbit	1:3000
**393**	TTF1	TTF1	NKX2-1	Epitomics/ Abcam	2044-1/ ab76013	Rabbit	1:150
**394**	Tuberin	Tuberin	TSC2	Epitomics/ Abcam	1613-1/ ab32554	Rabbit	1:2500
**395**	Tuberin/TSC2 (phospho T1462)	Tuberin_pT1462	TSC2	CST	3617	Rabbit	1:38
**396**	TUFM	TUFM	TUFM	Abcam	ab173300	Rabbit	1:38
**397**	TWEAK Receptor/FN14	FN14	TNFRSF12A	CST	4403	Rabbit	1:1000
**398**	TWIST	TWIST	TWIST1	Santa Cruz	sc-81417	Mouse	1:30
**399**	Tyro3	Tyro3	TYRO3	CST	5585	Rabbit	1:30
**400**	UBAC1	UBAC1	UBAC1	Sigma-Aldrich	HPA005651	Rabbit	1:250
**401**	Ubiquityl-Histone H2B	U-Histone-H2B	HIST1H2BB	CST	5546	Rabbit	1:500

**402**	UGT1A	UGT1A	UGT1A1, 3, 4, 5, 7, 8, 10	Santa Cruz	sc-271268	Mouse	1:75
**403**	ULK1 (phospho S757)	ULK1_pS757	ULK1	CST	6888	Rabbit	1:300
**404**	UQCRC2	UQCRC2	UQCRC2	MitoSciences / Abcam	MS304/ ab14745	Mouse	01:50
**405**	UVRAG	UVRAG	UVRAG	CST	13115	Rabbit	1:100
**406**	VASP	VASP	VASP	CST	3112	Rabbit	1:100
**407**	Vav1	Vav1	VAV1	CST	2502	Rabbit	1:500
**408**	VDAC1/Porin	Porin	VDAC1	Abcam	ab14734	Mouse	1:100
**409**	VEGF Receptor 2	VEGFR-2	KDR	CST	2479	Rabbit	1:3000
**410**	VHL/EPPK1	VHL-EPPK1	EPPK1	BD Biosciences	556347	Mouse	1:1500
**411**	Vimentin	Vimentin	VIM	Dako/Fisher	M0725/ MS-129-P	Mouse	1:250
**412**	Vinculin	Vinculin	VCL	Sigma-Aldrich	SAB4200080	Mouse	1:25000
**413**	Wee1	Wee1	WEE1	CST	4936	Rabbit	1:250
**414**	Wee1 (phospho S642)	Wee1_pS642	WEE1	CST	4910	Rabbit	1:50
**415**	WIPI1	WIPI1	WIPI1	CST	12124	Rabbit	1:150
**416**	WIPI2	WIPI2	WIPI2	CST	8567	Rabbit	1:150
**417**	XBP-1	XBP-1	XBP1	Santa Cruz	sc-32136	Goat	1:200
**418**	XIAP	XIAP	XIAP	CST	2042	Rabbit	1:100
**419**	XPA	XPA	XPA	Santa Cruz	sc-56813	Mouse	1:75
**420**	XPF	XPF	ERCC4	Abcam	ab73720	Rabbit	1:100
**421**	XPG	ERCC5	ERCC5	Proteintech Group	11331-1-AP	Rabbit	1:250
**422**	XRCC1	XRCC1	XRCC1	CST	2735	Rabbit	1:20
**423**	YAP	YAP	YAP1	Santa Cruz	sc-376830	Mouse	1:300
**424**	YAP (phospho S127)	YAP_pS127	YAP1	CST	4911	Rabbit	1:250
**425**	YB1 (phospho S102)	YB1_pS102	YBX1	CST	2900	Rabbit	1:50
**426**	ZAP-70	ZAP-70	ZAP70	CST	2705	Rabbit	1:500

Table S11: Cell cycle distribution of MGT11 treated cells - Statistical analysis was performed by two-way ANOVA followed by Tukey test.

		no	A11	R547	A11 + R547
**Sub G0**	no		ns	ns	*
	A11			ns	ns
	R547				ns
	A11 + R547				
**G0**	no		ns	ns	ns
	A11			ns	ns
	R547				ns
	A11 + R547				
**G1**	no		ns	ns	ns
	A11			ns	ns
	R547				ns
	A11 + R547				
**S**	no		ns	ns	ns
	A11			ns	ns
	R547				*
	A11 + R547				
**G2**	no		ns	**	ns
	A11			**	ns
	R547				***
	A11 + R547				
**M**	no		ns	ns	ns
	A11			ns	ns
	R547				ns
	A11 + R547				

Full unedited gels for Figures 5 and 6

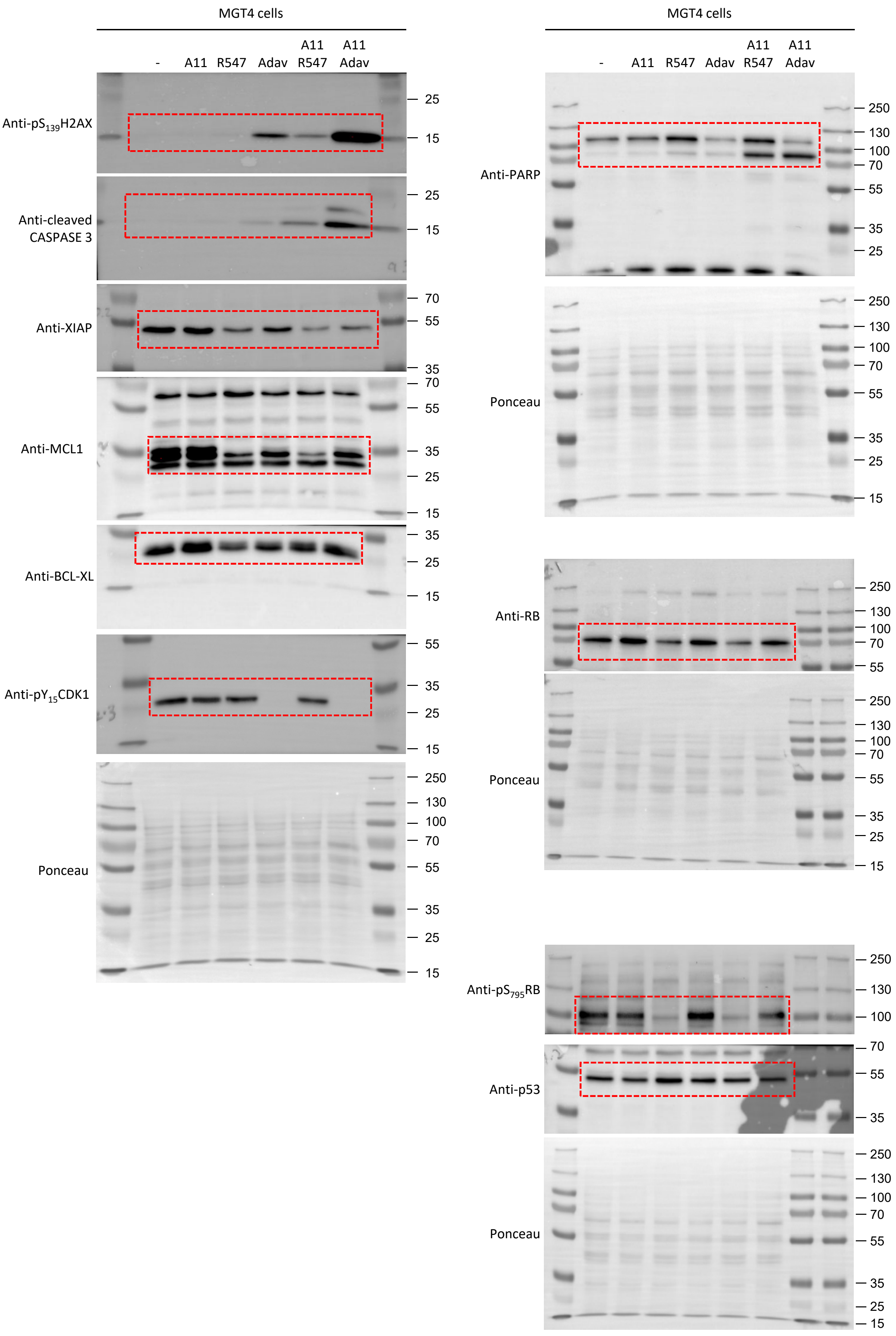

Full unedited gels for Figures 5 and 6

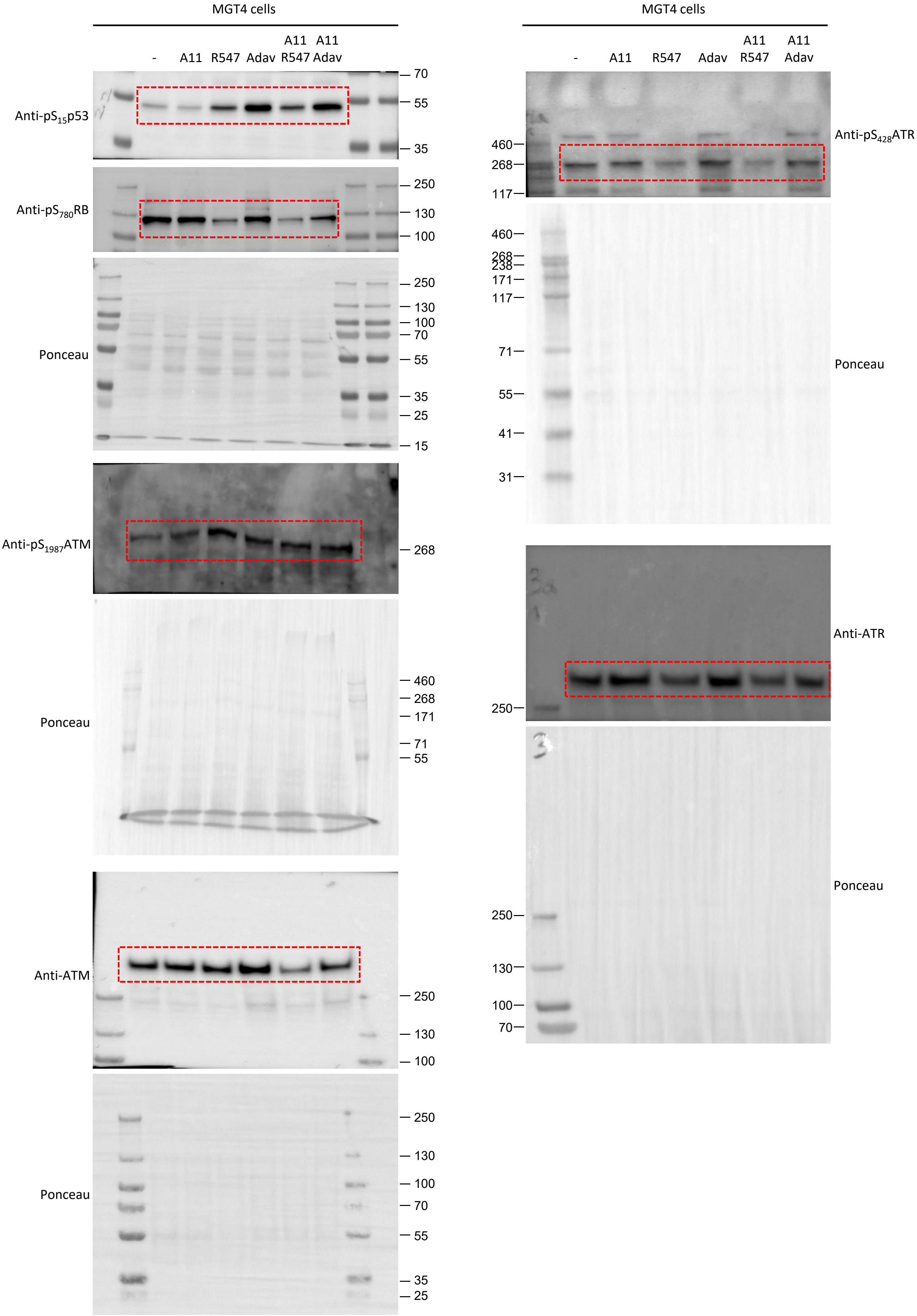

Full unedited gels for Figures 5 and 6

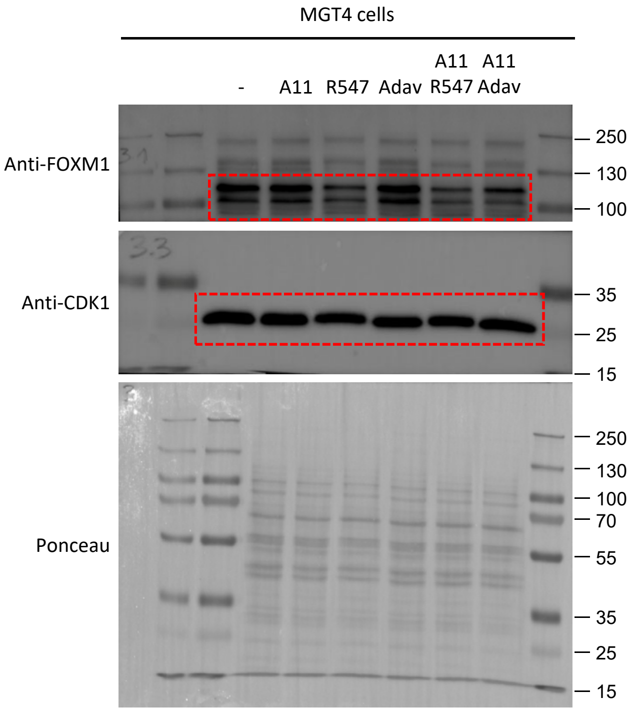

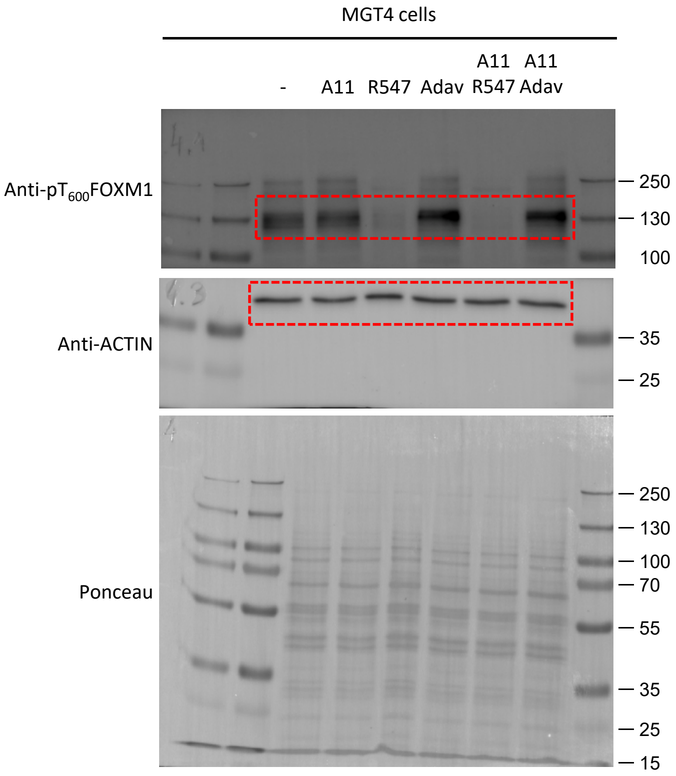

Full unedited gels for Figures 5 and 6

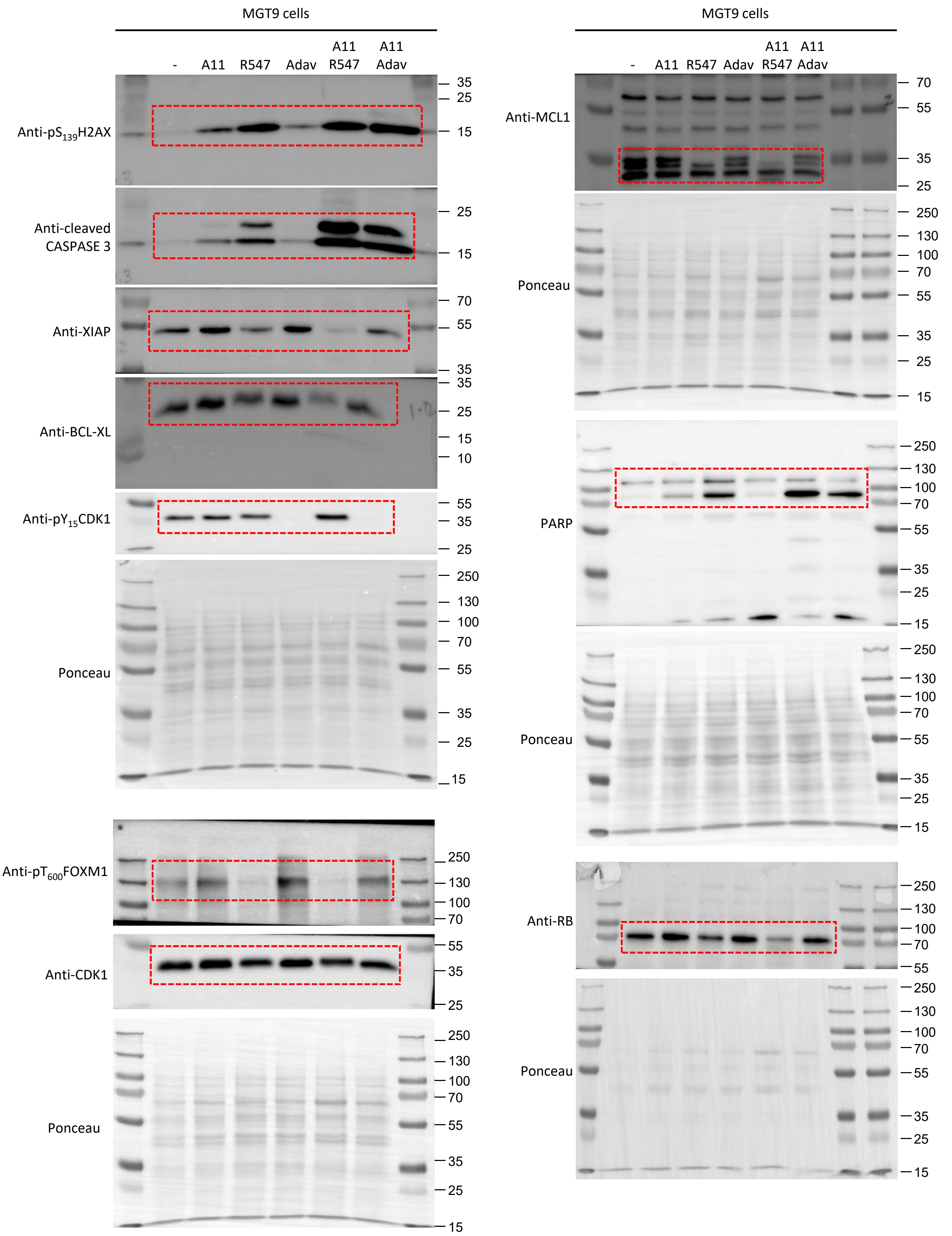

Full unedited gels for Figures 5 and 6

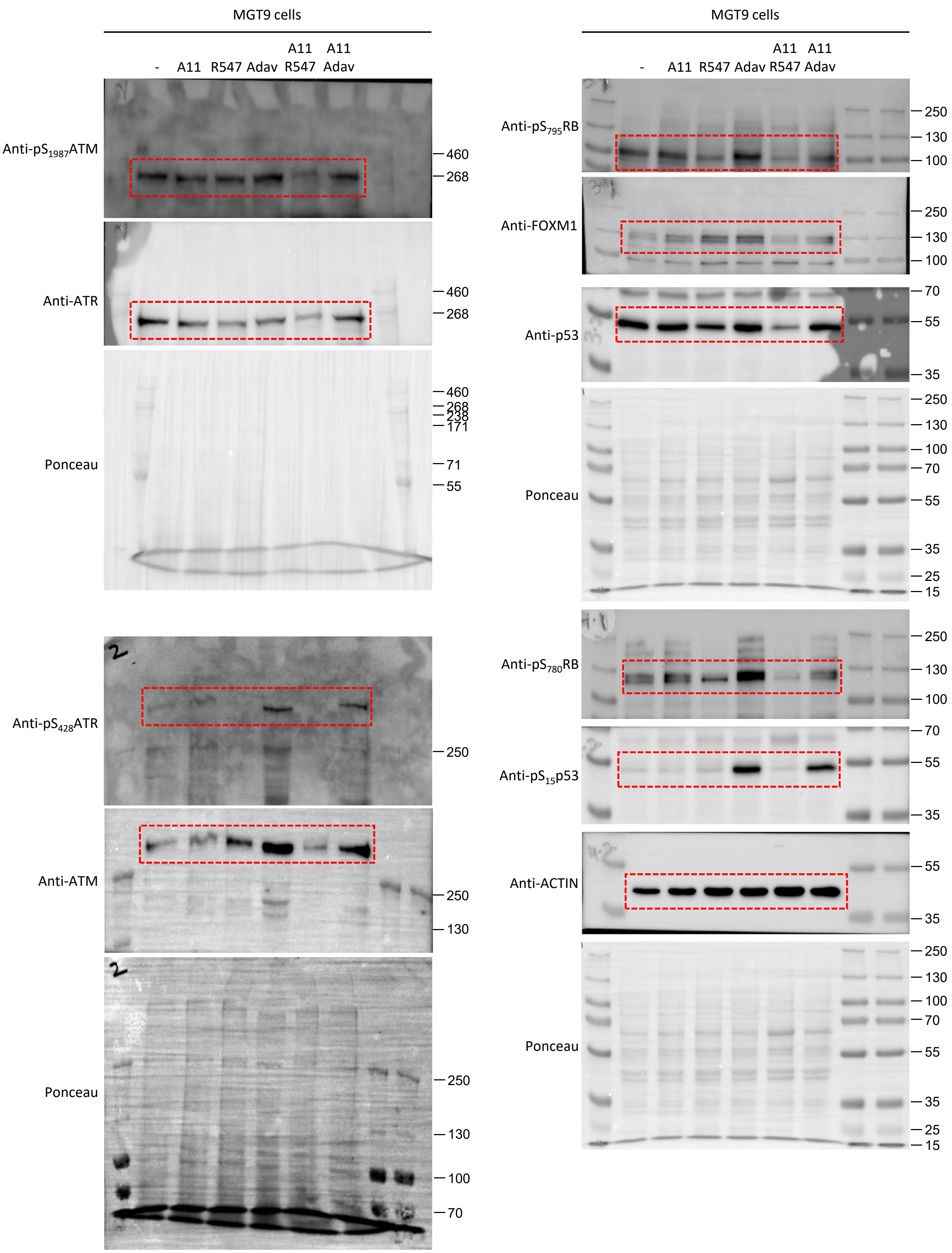

Full unedited gels for Figures 5 and 6

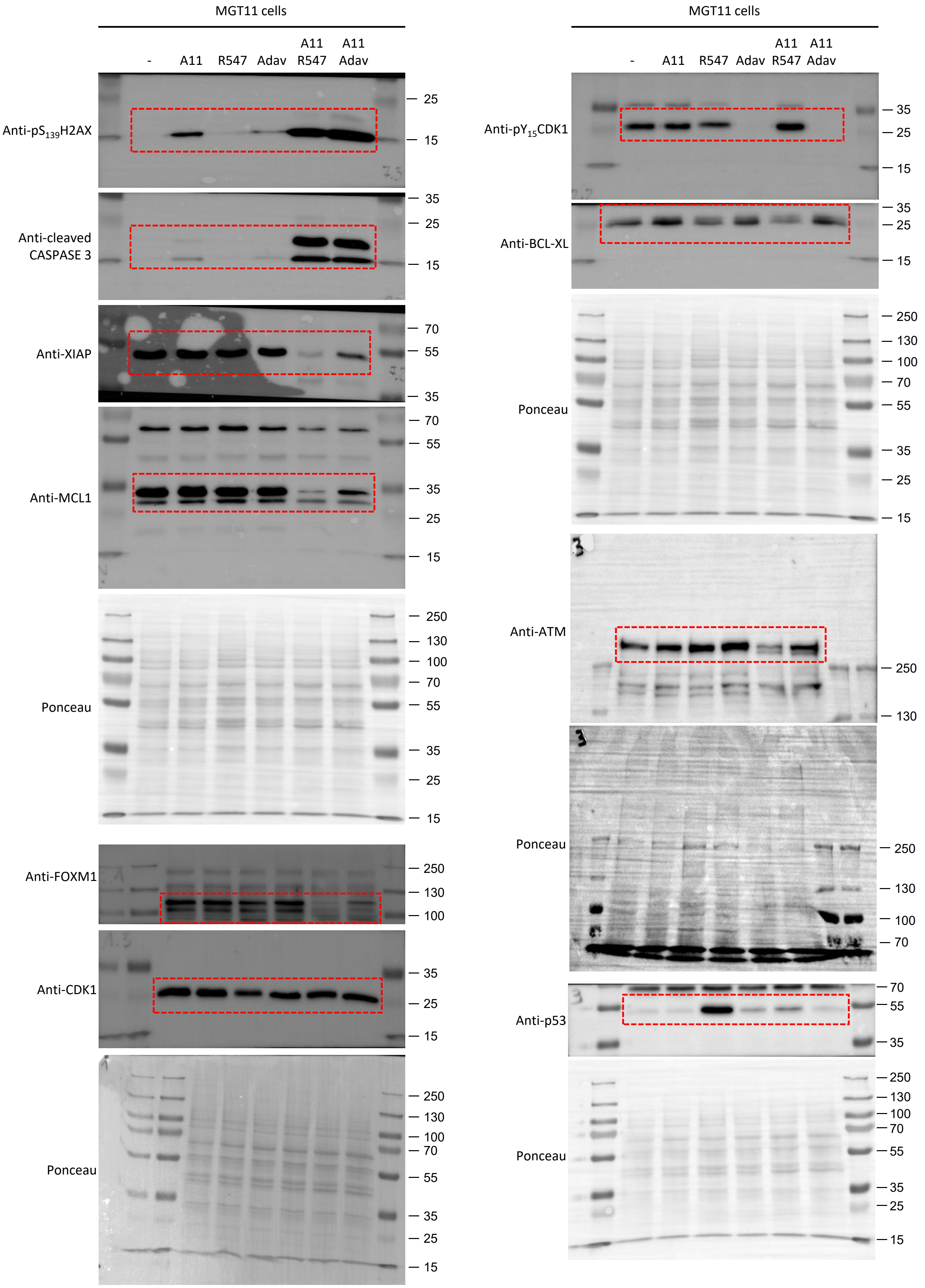

Full unedited gels for Figures 5 and 6

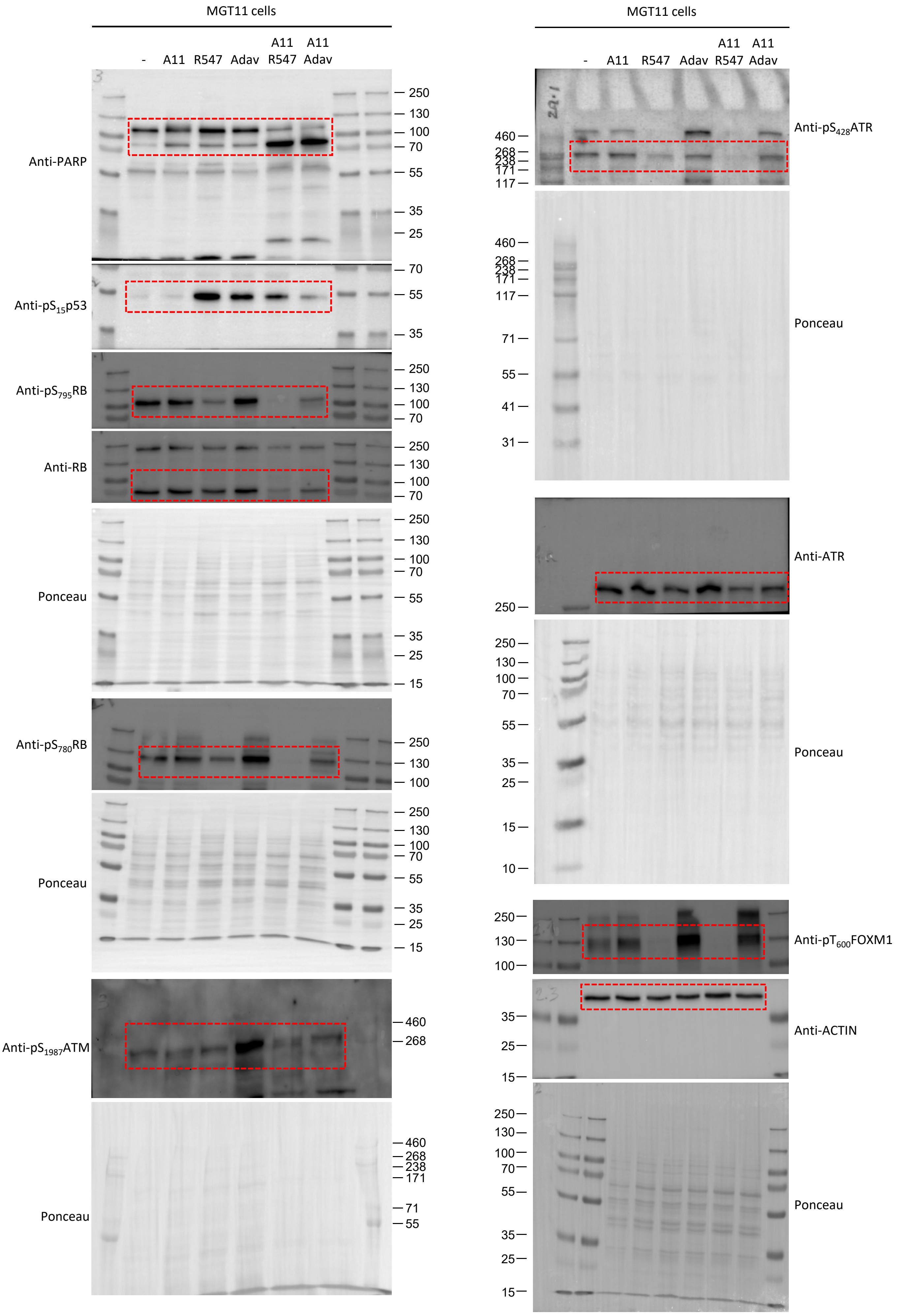

Full unedited gels for Figure S5

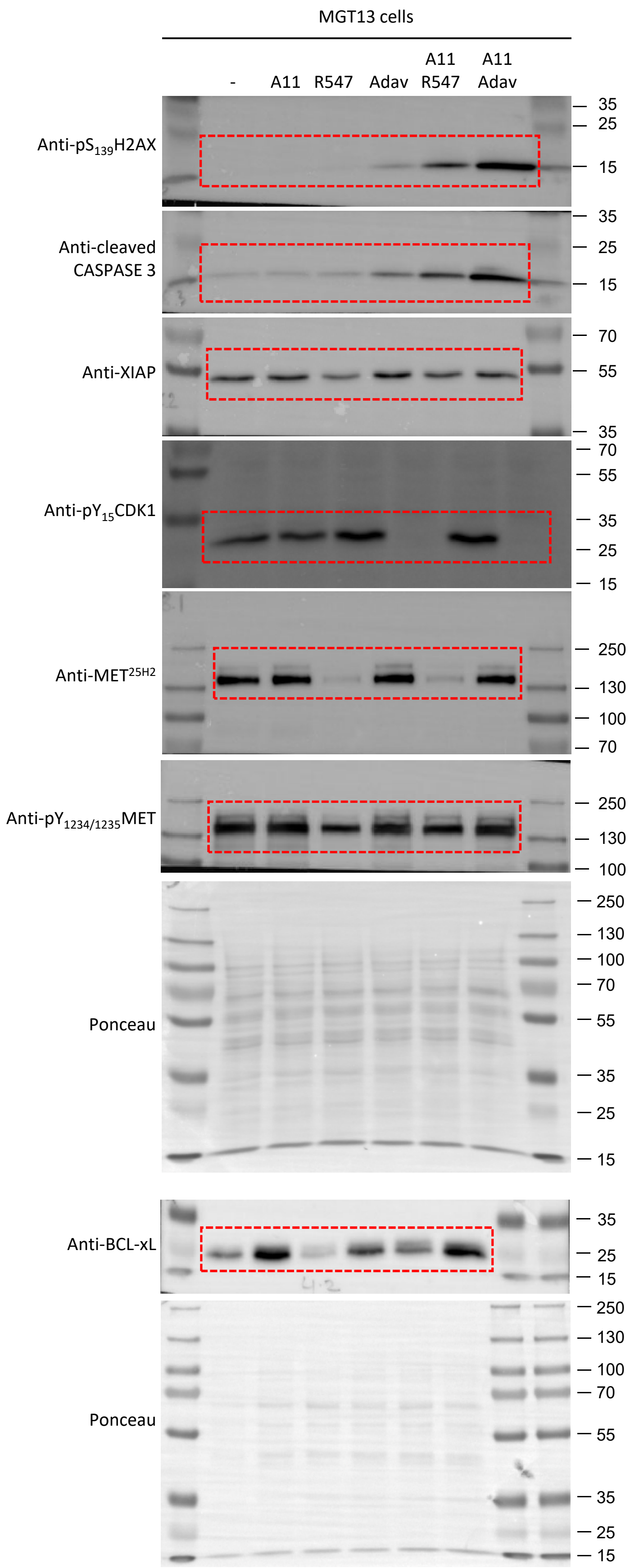

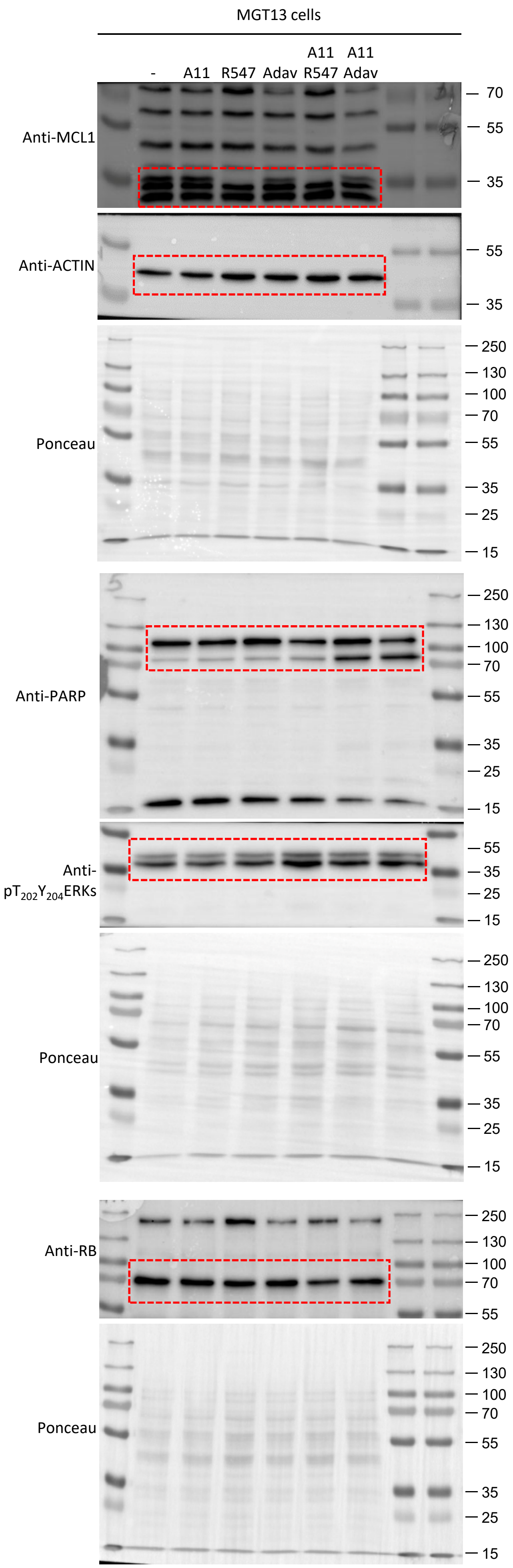

Full unedited gels for Figure S5

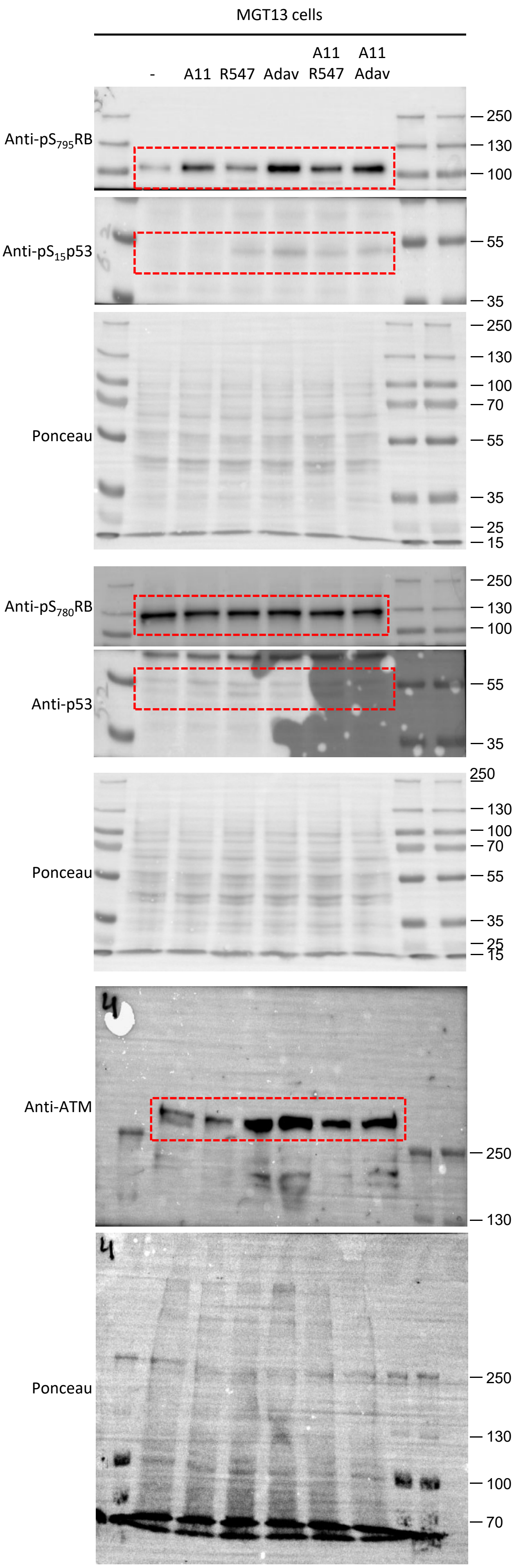

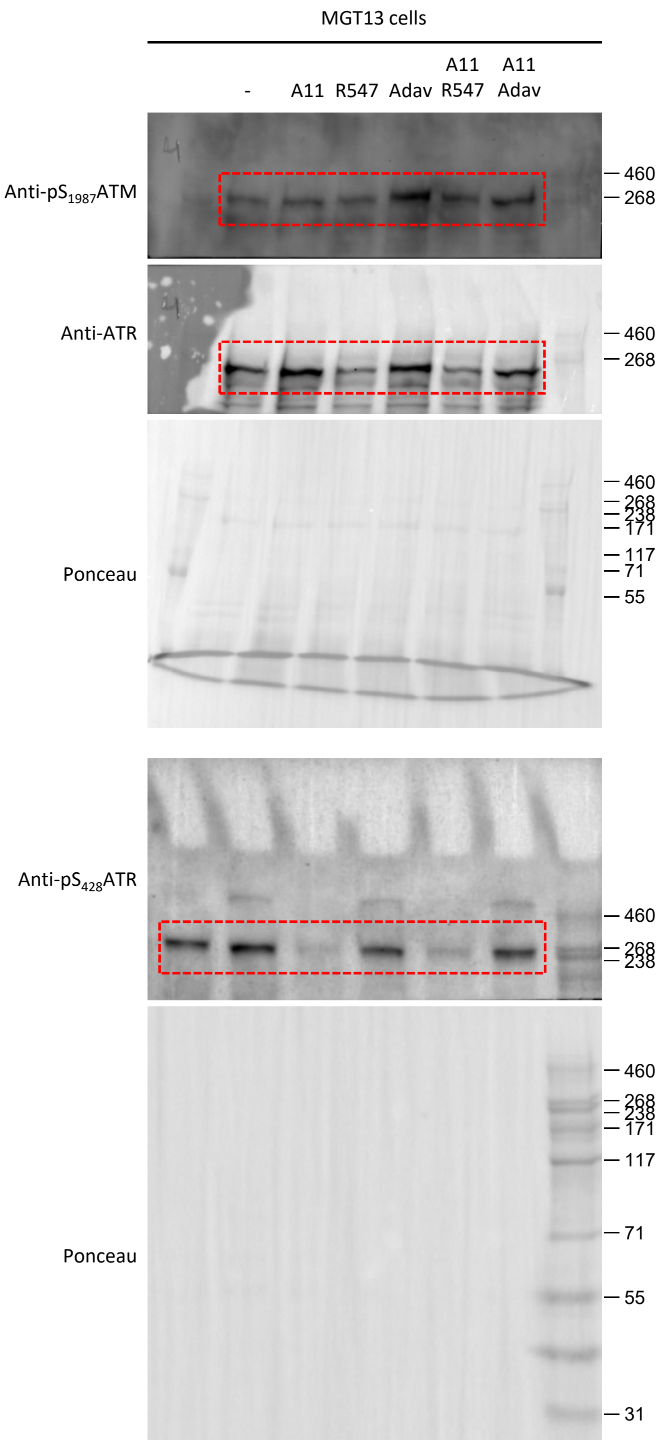

Full unedited gels for Figure 8A

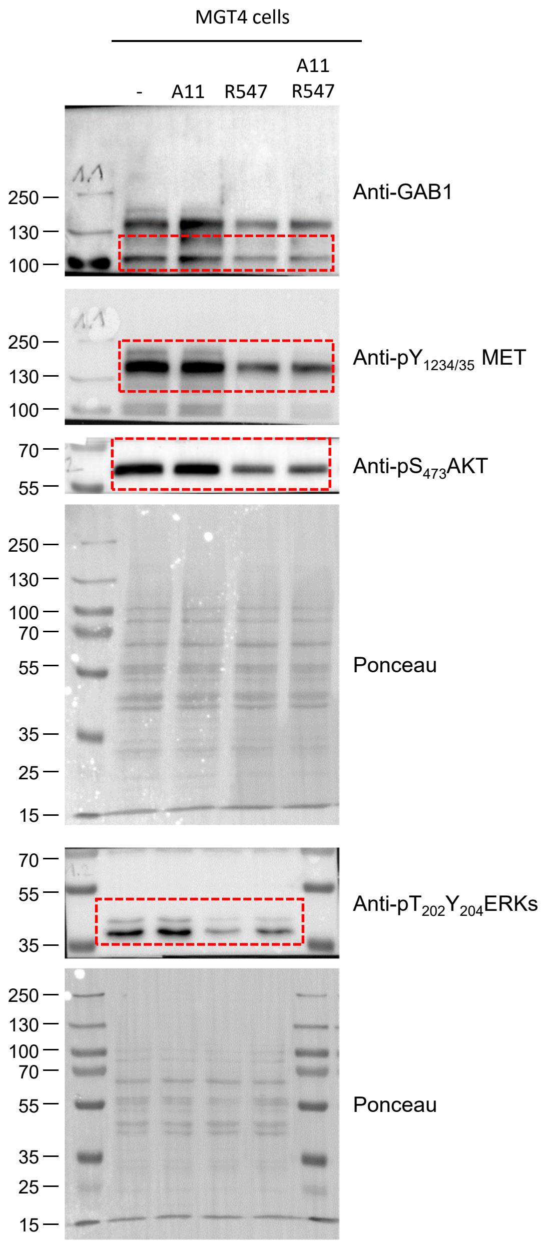

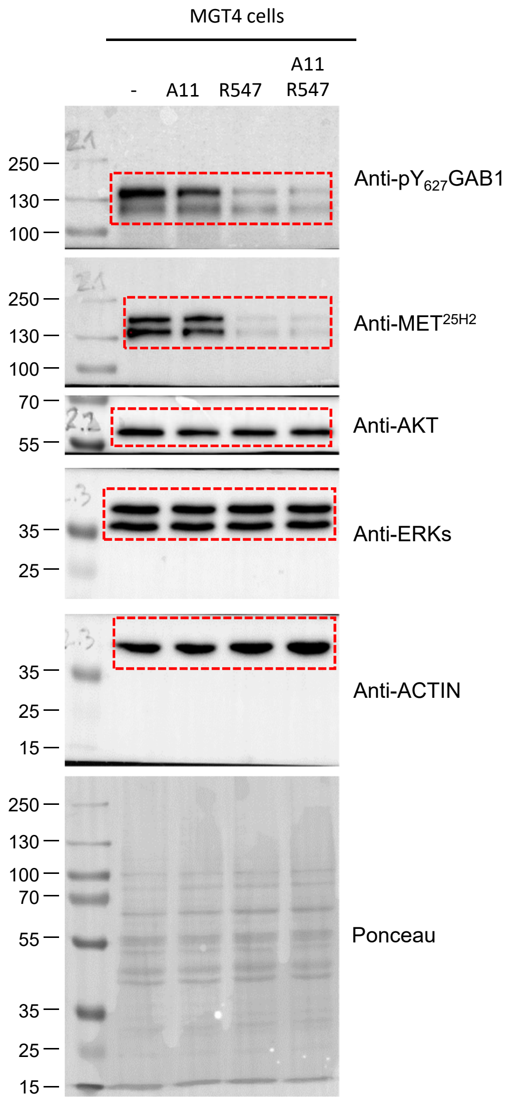

Full unedited gels for Figure 8A

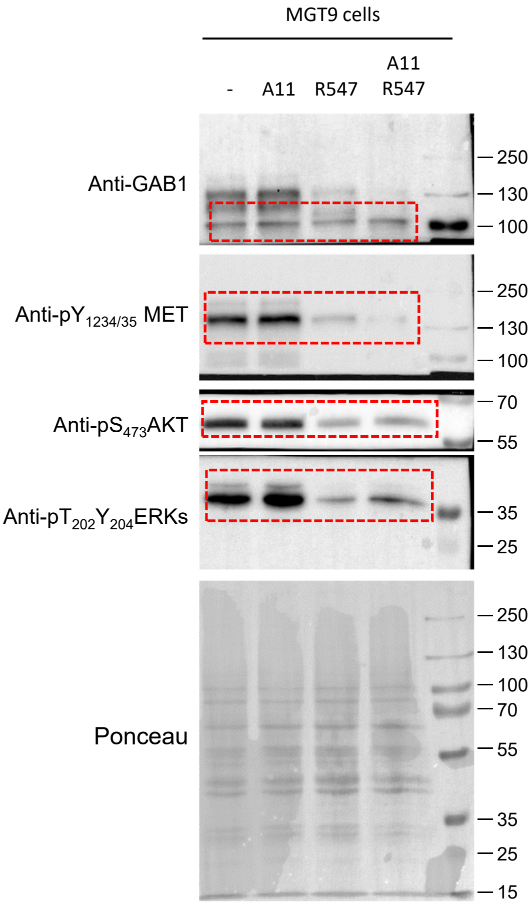

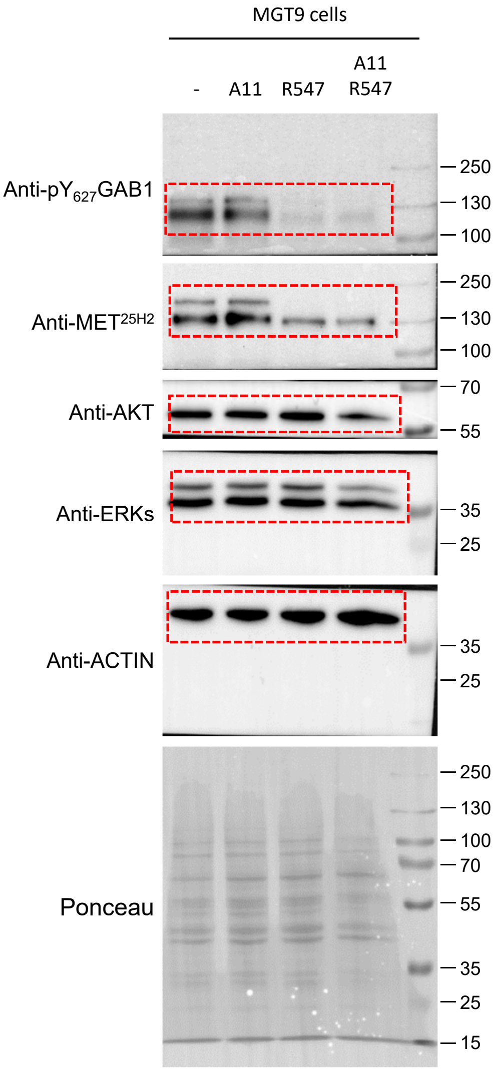

Full unedited gels for Figure 8A

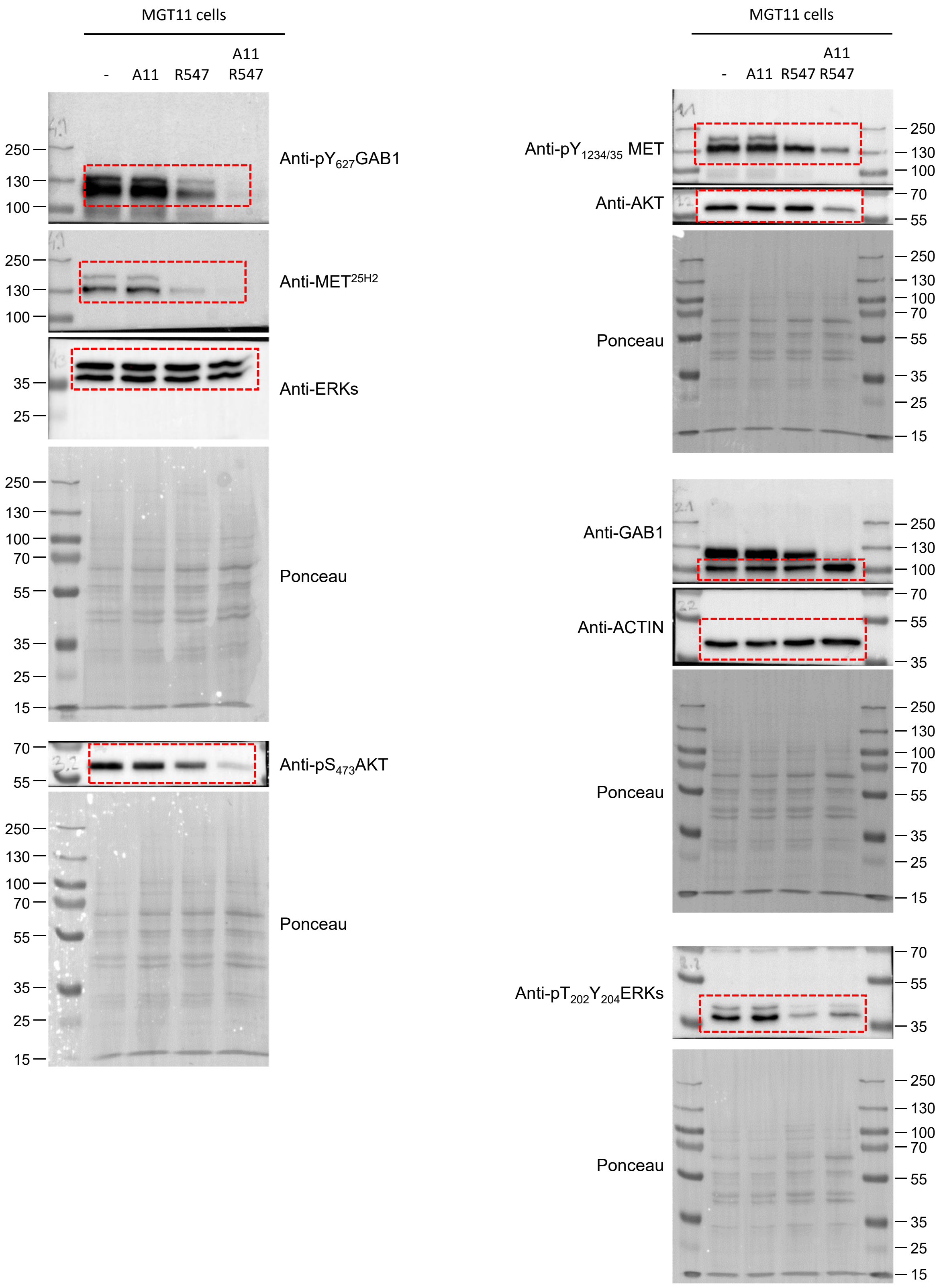

Full unedited gels for Figure 8B

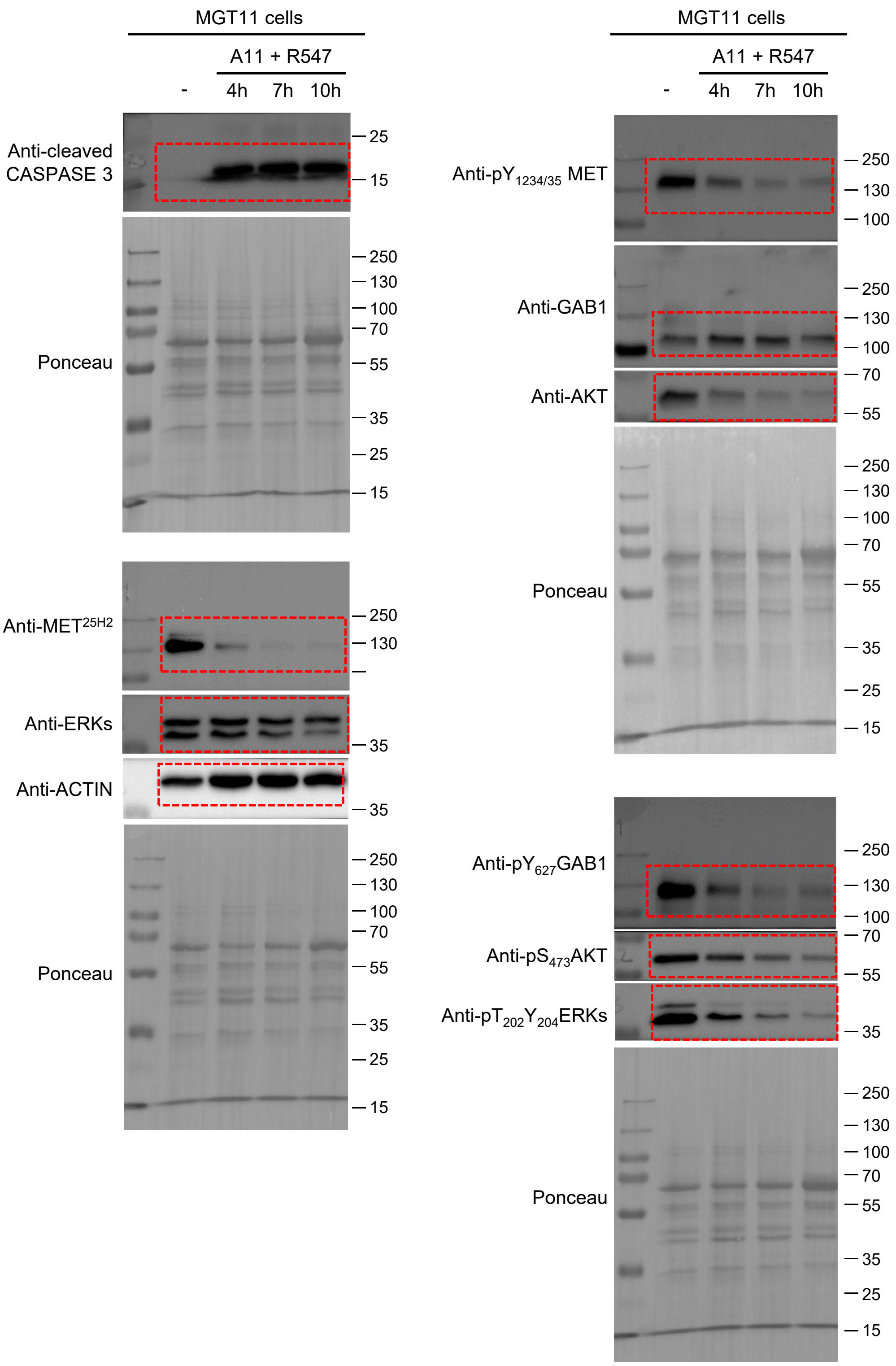

Full unedited gels for Figure S7

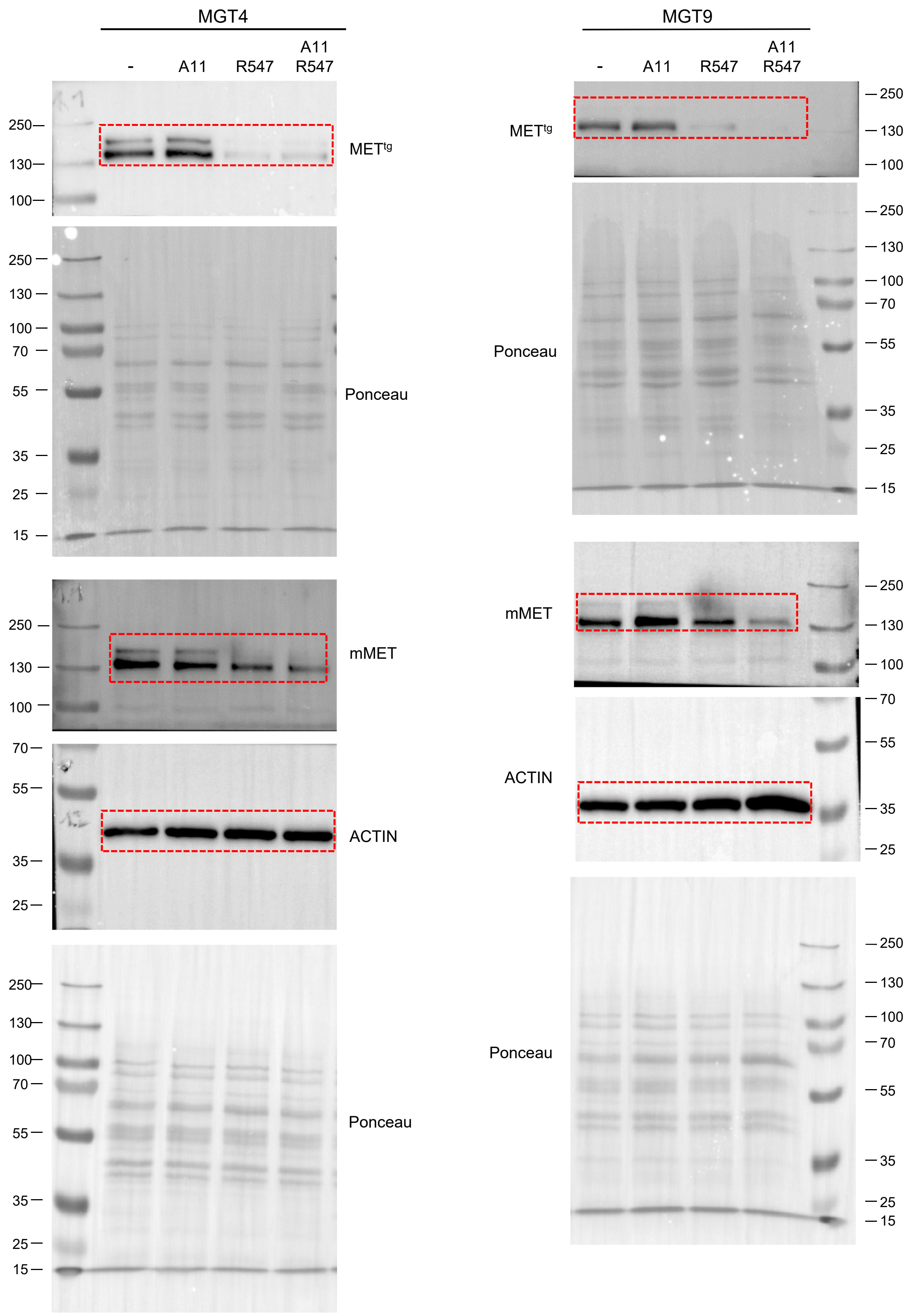

Full unedited gels for Figure S7

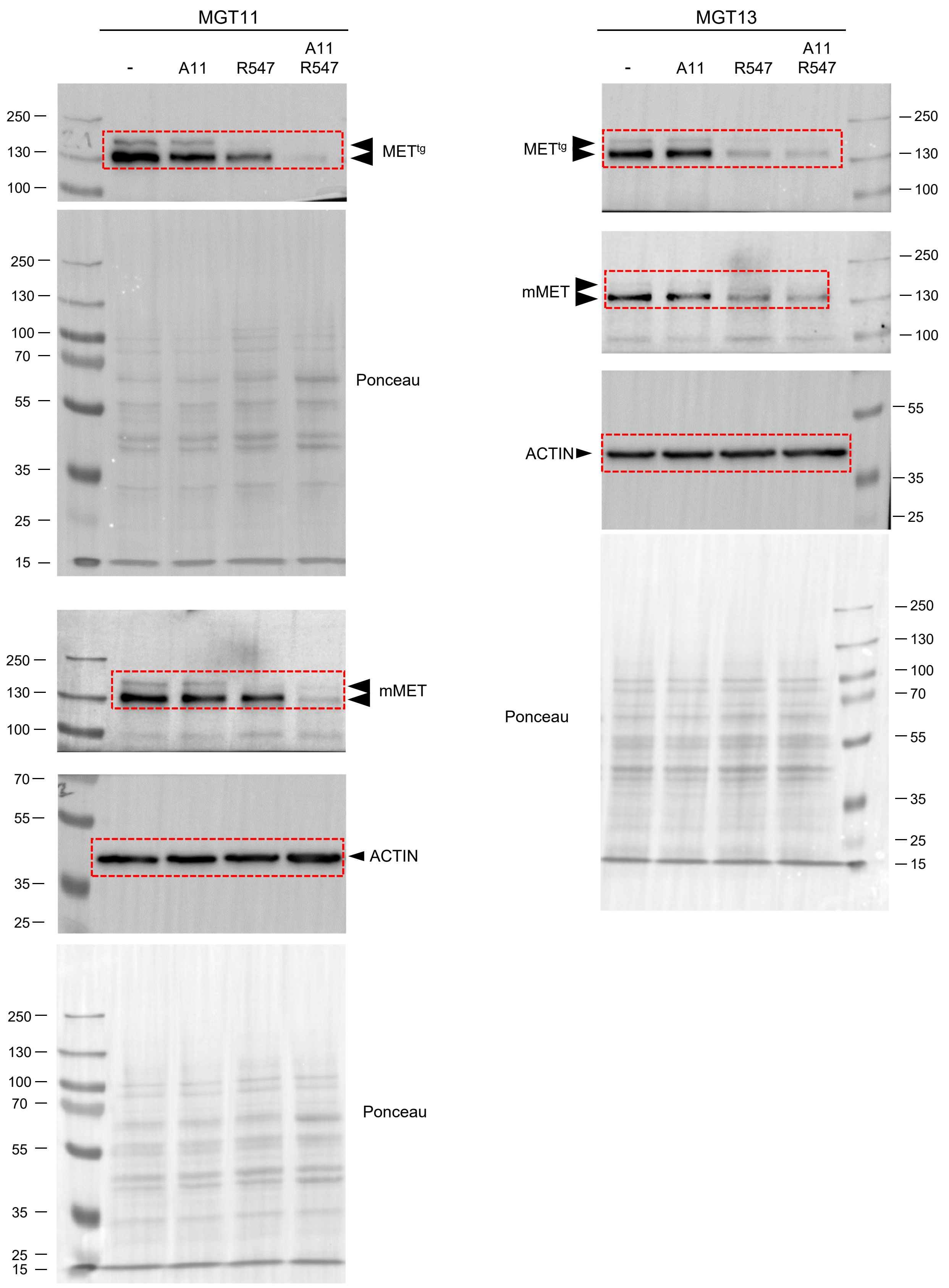

Full unedited gels for Figure 8F

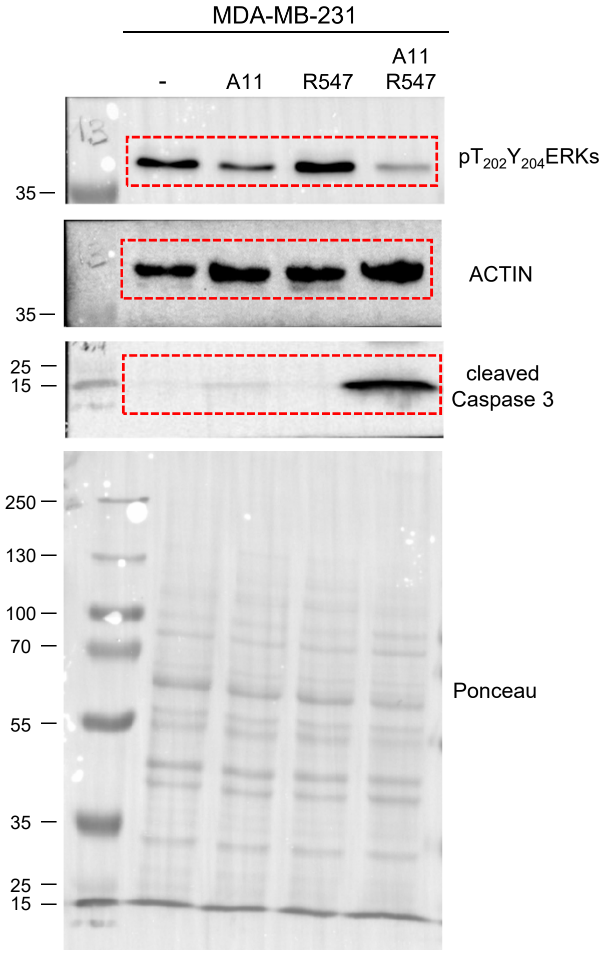

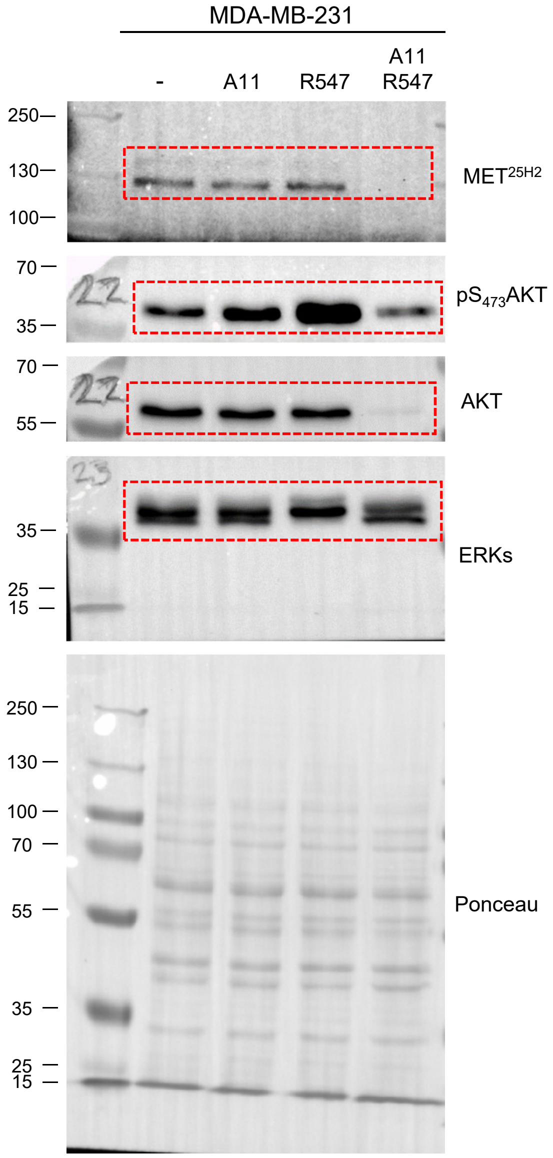

Full unedited gels for Figure 8F

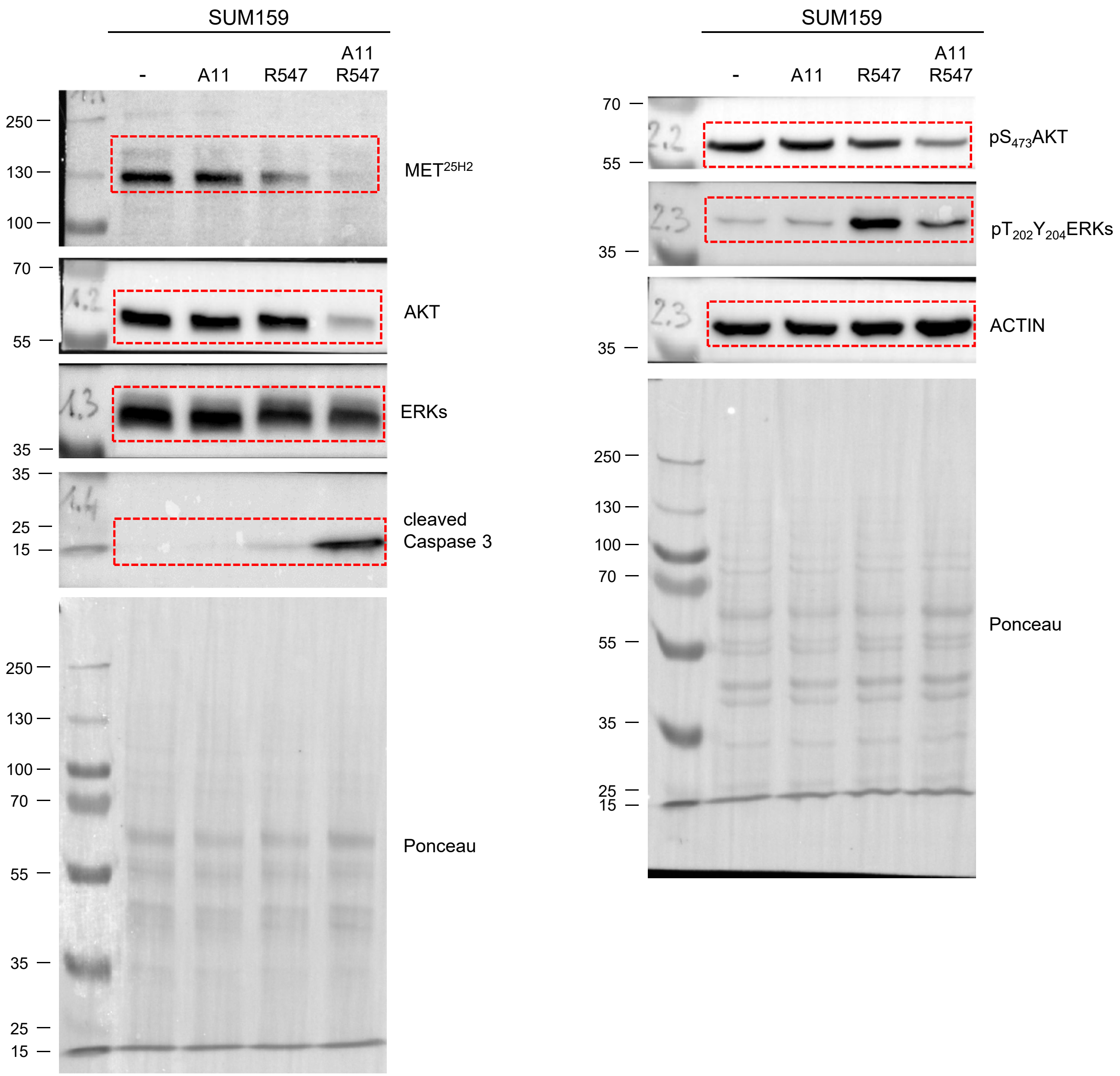

Full unedited gels for Figure 8F

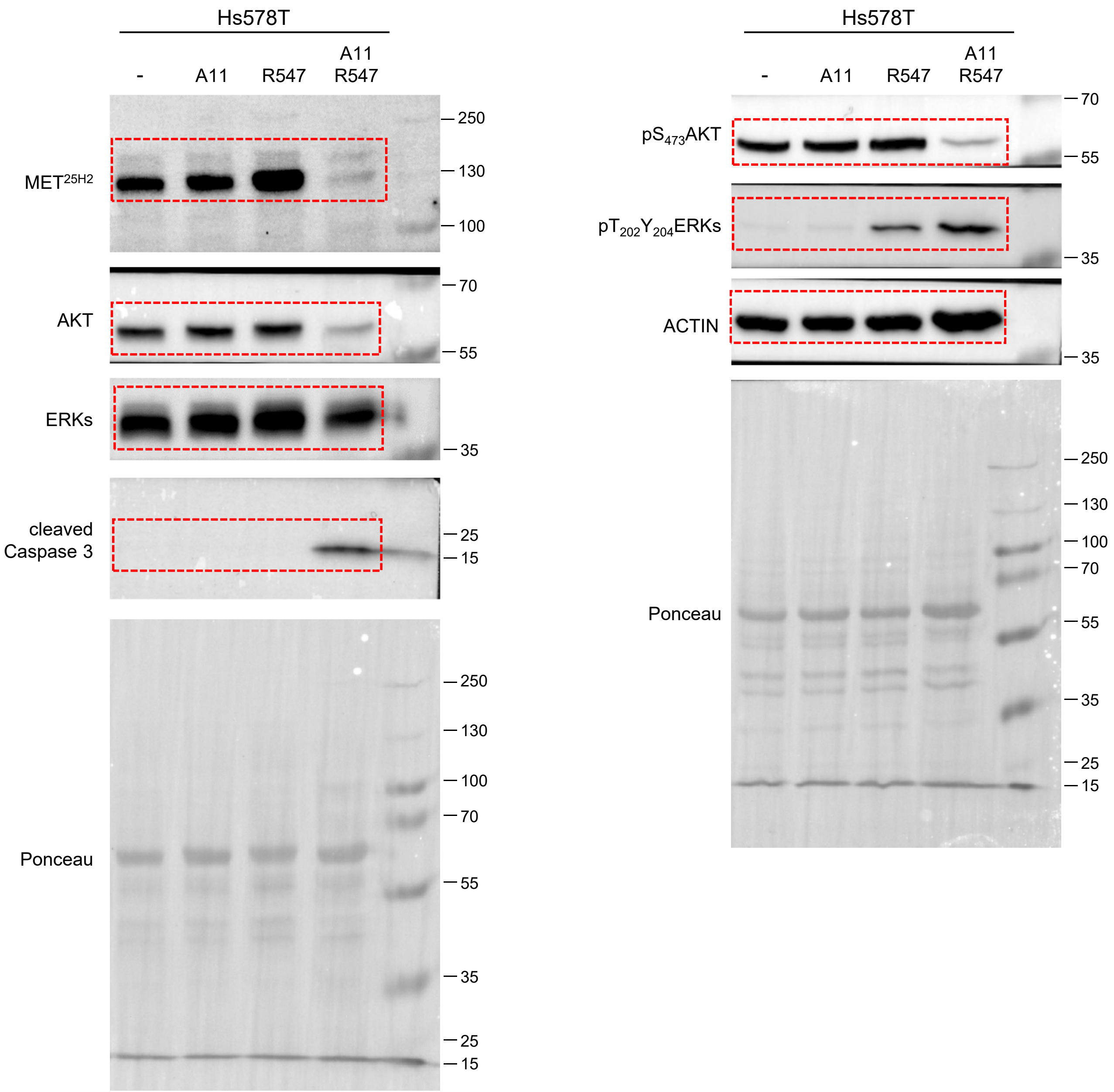

Full unedited gels for Figure 8D

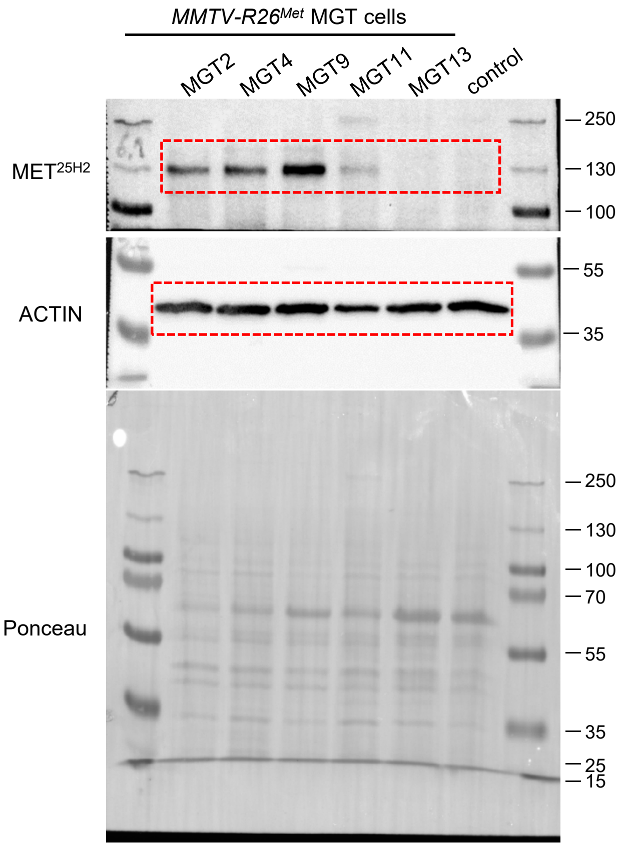

Supplement: Supplementary file 1 — Supplementary figures and tables. [file thnov11p9180s1.pdf]
